# Macroscopic resting-state brain dynamics are best described by linear models

**DOI:** 10.1038/s41551-023-01117-y

**Published:** 2023-12-11

**Authors:** Erfan Nozari, Maxwell A. Bertolero, Jennifer Stiso, Lorenzo Caciagli, Eli J. Cornblath, Xiaosong He, Arun S. Mahadevan, George J. Pappas, Dani S. Bassett

**Affiliations:** 1 Department of Mechanical Engineering, University of California, Riverside, CA USA; 2 Department of Electrical and Computer Engineering, University of California, Riverside, CA USA; 3 Department of Bioengineering, University of California, Riverside, CA USA; 4 Department of Bioengineering, University of Pennsylvania, Philadelphia, PA USA; 5 Department of Neuroscience, University of Pennsylvania, Philadelphia, PA USA; 6 Department of Electrical and Systems Engineering, University of Pennsylvania, Philadelphia, PA USA; 7 Department of Physics and Astronomy, University of Pennsylvania, Philadelphia, PA USA; 8 Department of Neurology, University of Pennsylvania, Philadelphia, PA USA; 9 Department of Psychiatry, University of Pennsylvania, Philadelphia, PA USA; 10 Santa Fe Institute, Santa Fe, NM USA

**Keywords:** Computational neuroscience, Electrical and electronic engineering

## Abstract

It is typically assumed that large networks of neurons exhibit a large repertoire of nonlinear behaviours. Here we challenge this assumption by leveraging mathematical models derived from measurements of local field potentials via intracranial electroencephalography and of whole-brain blood-oxygen-level-dependent brain activity via functional magnetic resonance imaging. We used state-of-the-art linear and nonlinear families of models to describe spontaneous resting-state activity of 700 participants in the Human Connectome Project and 122 participants in the Restoring Active Memory project. We found that linear autoregressive models provide the best fit across both data types and three performance metrics: predictive power, computational complexity and the extent of the residual dynamics unexplained by the model. To explain this observation, we show that microscopic nonlinear dynamics can be counteracted or masked by four factors associated with macroscopic dynamics: averaging over space and over time, which are inherent to aggregated macroscopic brain activity, and observation noise and limited data samples, which stem from technological limitations. We therefore argue that easier-to-interpret linear models can faithfully describe macroscopic brain dynamics during resting-state conditions.

## Abstract (web summary)

Linear mathematical models derived from measurements of local field potentials and of whole-brain blood-oxygen-level-dependent neural activity predict resting-state neural dynamics at least as accurately as nonlinear models.

## Main

Throughout the recent history of neuroscience, computational models have been developed and used ubiquitously to decompose the complex neural mechanisms underlying cognition and behaviour^1–5^. A dilemma that is inherent to computational modelling but particularly challenging in computational neuroscience is the trade-off between (cross-validated) accuracy and simplicity. Both finely detailed models^6^ and broadly simplified ones^7,8^ have their respective proponents. One of the many facets of this trade-off pertains to the use of linear vs nonlinear models. Nonlinearity of dynamics is inevitable at the microscale of individual neurons^9^ and their components^10^, and has been demonstrated, although less comprehensively, at the mesoscale of neuronal populations^11^. Further supported by theoretical derivations^12^ and motivated by the much larger repertoire of behaviours of nonlinear systems (including chaos, multistability and metastability), an assumption has thus formed^13–16^ that accurate models of neurodynamics at the macroscale (i.e., of brain regions) must inevitably be nonlinear.

This assumption begs the question of whether nonlinear models will in fact perform better than linear ones in accounting for the dynamics of neuroimaging and neurophysiological data. Specifically, can nonlinear models explain neuroimaging or neurophysiological data more accurately than linear ones? This pragmatic modelling question, importantly, is different from the general question of whether any signs of ‘nonlinearity’ can be found in neuroimaging time series^17–19^ (see Discussion).

Few investigations^20–22^ have indeed sought to answer the former question directly by comparing the ‘fit’ of linear and nonlinear models to neurophysiological (electroencephalography, EEG, and intracranial electroencephalography, iEEG) time series. However, even these few works are limited in that each provides a single comparison between a linear and a nonlinear family of models (linear autoregressive (AR) vs nonlinear manifold-based model in ref. ^20^, linear finite-impulse response vs nonlinear Volterra series model in ref. ^21^, and linear state space vs nonlinear AR with radial basis function nonlinearities in ref. ^22^), which need not be the best representatives of linear and nonlinear models in general. While the compared linear and nonlinear models were found to be as predictive of EEG data in ref. ^20^ and iEEG data in ref. ^22^, the results were mixed in ref. ^21^. Using scalp EEG data from patients with epilepsy, this paper finds mostly linear dynamics well within and outside of seizures, and mostly nonlinear dynamics (although varying across patients) in the periods around seizure onsets and offsets. While limited in their scope, these works beg for a deep and rigorous data-driven investigation into the nonlinearity of macroscopic brain dynamics, as pursued herein.

In the second part of the paper, we seek to answer the question of why nonlinear models do not provide more accurate predictions than linear ones even though neurodynamics are inevitably nonlinear at the microscale. Specifically, we numerically demonstrate, using a simple sigmoidal nonlinearity, that four properties of macroscopic brain dynamics can fundamentally ‘counteract’ or seemingly ‘mask’ nonlinear dynamics present at the microscale: averaging over the activity of large populations of neurons to obtain a single macroscopic time series (averaging over space), natural low-pass-filtering properties of brain processes (averaging over time), observation noise and limited data samples. While the effects of observation noise and limited data samples are technology dependent but otherwise independent from the form of nonlinearity, the effects of spatiotemporal averaging are fundamental to macroscopic neural dynamics and may depend on the functional form of the microscale nonlinearity. We thus also verify and demonstrate the effects of spatiotemporal averaging using a data-driven and biophysically grounded spiking neuron model^23^. Together, our results provide (1) important evidence that linear models can be as descriptive as nonlinear ones at the macroscale and (2) a methodology based on system identification theory to quantitatively define a ‘best’ model of whole-brain dynamics given a set of specified costs.

## Results

### System identification and data-driven computational modelling

**Fig. 1:**
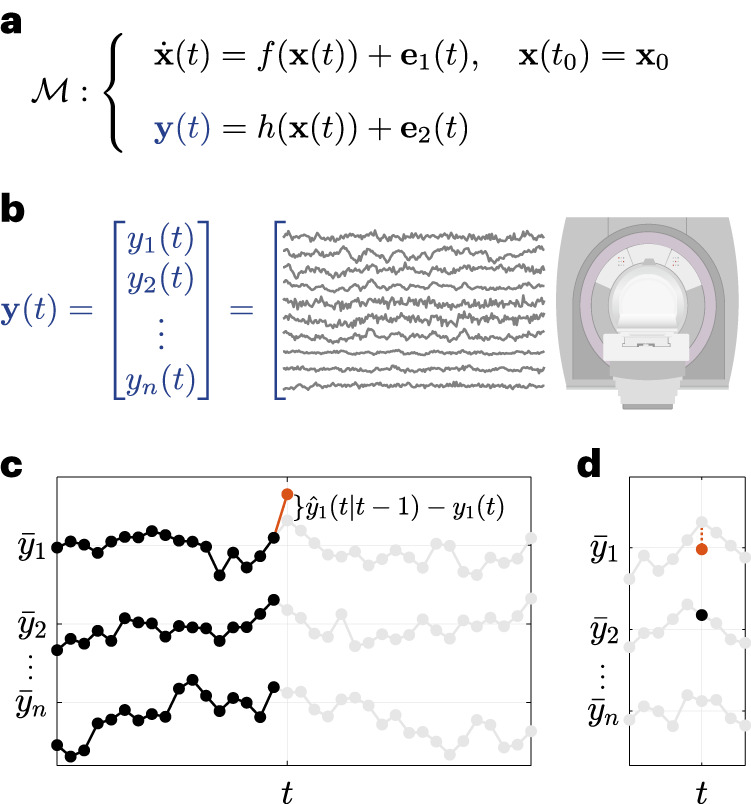
Prediction error method for system identification. **a**,**b**, The general category of computational models ${{{\mathcal{M}}}}$ studied in this work, represented by an ODE describing the resting-state evolution of internal states **x**(*t*) (**a**) and an output model that maps internal states to fMRI/iEEG time series **y**(*t*), as shown for fMRI (**b**). A total of *n*_fMRI_ = 116 regions were used throughout (see Methods) for fMRI, while 13 ≤ *n*_iEEG_ ≤ 175 channels were used for each iEEG patient. **c**, A schematic representation of the prediction error system identification framework used in this work. At each time *t*, all of {**y**(0),…,**y**(*t* − 1)} was used to predict **y**(*t*) simultaneously across all channels, denoted for simplicity by $\hat{{{{\bf{y}}}}}(t| t-1)$. ${\bar{y}}_{i}$ denotes the temporal average of *y*_i_(*t*) for each channel *i*. This estimate should not be confused with estimates of FC. **d**, FC measures the covariation between pairs of channels or, equivalently, how well each *y*_*i*_(*t*) (*y*_1_(*t*) in the figure) can be predicted from each other *y*_*j*_(*t*) (*y*_2_(*t*) in the figure), ‘at the same time’ *t*.

Among the diverse categories of computational models used in neuroscience, we focus on ordinary differential equation (ODE) models of the general form

1a $$\dot{{{{\bf{x}}}}}(t)=f({{{\bf{x}}}}(t))+{{{{\bf{e}}}}}_{1}(t),\qquad {{{\bf{x}}}}(0)={{{{\bf{x}}}}}_{0}$$

1b $${{{\bf{y}}}}(t)=h({{{\bf{x}}}}(t))+{{{{\bf{e}}}}}_{2}(t)$$

where **y**(*t*) is an *n*-dimensional time series of recorded brain activity, in this case via resting-state functional magnetic resonance imaging (fMRI) (rsfMRI) or iEEG (rsiEEG), **x**(*t*) is an *m*-dimensional time series of ‘internal’ brain states, *f* and *h* are generally nonlinear vector fields, and **e**_1_(*t*) and **e**_2_(*t*) are time series of process and measurement noise with arbitrary statistics (Fig. 1a,b). It is possible, although not necessary, that *m* = *n*. As with any differential equation, the description would not be complete without the initial condition **x**(0) = **x**_0_, determining the state of the brain at the initial recording time *t* = 0. Note that no external input **u**(*t*) is considered due to the resting-state condition of the experiments. Also, given that we can only sample **y**(*t*) in discrete time, we implement equation (1) by approximating the derivative $\dot{{{{\bf{x}}}}}(t)$ as a first difference **x**(*t*) − **x**(*t* − 1) (see Methods).

The model in equation (1) is ‘linear’ if the functions *f* and *h* are linear functions, that is, matrix operations of the form *f*(**x**) = *A***x** and *h*(**x**) = *C***x** where *A*_*m*×*m*_ and *C*_*n*×*m*_ are constant (or even time-varying, but state-independent) matrices. Throughout the field of computational neuroscience, numerous models of the form in equation (1) or its discretization (see Methods) are constructed and used, each with different functional forms and noise statistics^6,7,9,12,15,22,24^. The critical but fairly overlooked quest of system identification^25^ is then to find the ‘best’ model, among all the available options, against experimental data. This comparison indeed depends on one’s measure of a model’s goodness of fit.

A natural choice, referred to as the prediction error (PE) approach, is based on how well a given model can predict the ‘future’ of the measured time series from its ‘current and past’ values (Fig. 1c). Note that this prediction is precisely what an ODE such as equation (1a) defines: it models the ‘change’ $\dot{{{{\bf{x}}}}}(t)$, and thus the immediate future, of the system’s state (and therefore output) from its current state **x**(*t*). Since the state **x**(*t*) is not directly available, it should in turn be estimated from the current and past measurements of the output **y**(*t*). Therefore, the PE approach in its simplest form seeks to minimize, within any given parametric or non-parametric family of models, the magnitude of the one-step-ahead PE

2 $${{{\boldsymbol{\varepsilon }}}}(t)={{{\bf{y}}}}(t)-\hat{{{{\bf{y}}}}}(t| t-1)$$

where $\hat{{{{\bf{y}}}}}(t| t-1)$ is the Bayes-optimal, minimum variance estimate of **y**(*t*) given all of the history {**y**(0),…,**y**(*t* − 1)} of **y** up to time *t* − 1 (ref. ^25^) (Fig. 1c). Notably, the PE approach focuses on the prediction accuracy of the time series itself, rather than the prediction accuracy of functional connectivity (Fig. 1d) or other statistics of the time series (cf. Discussion and Supplementary Fig. 1). This approach can also be easily extended to multistep-ahead predictions (cf. ref. ^25^ and Supplementary Figs. 20 and 21).

The task of system identification does not end once the parameters of a given family of models are fit to the (training) data. The critical next step is to assess the quality of the fit, particularly to data withheld during the training (cross-validation). In the PE approach, the two most widely used measures are the variance and the whiteness of the PE (ref. ^25^), where the former is often measured by

3 $${R}_{i}^{2}=1-\frac{{\sum }_{t}{\varepsilon }_{i}{(t)}^{2}}{{\sum }_{t}{({y}_{i}(t)-{\bar{y}}_{i})}^{2}}$$

and the latter is often assessed via a *χ*^2^ test of whiteness (Methods), for each channel *i* = 1,…,*n*. In equation (3), *ε*(*t*) is the same one-step-ahead prediction error in equation (2) and ${\bar{y}}_{i}$ is the temporal average of *y*_*i*_(*t*) (often equal to zero due to mean centreing) and corresponds to a constant predictor which always predicts **y**(*t*) equal to its average $\bar{{{{\bf{y}}}}}$. Therefore, it is clear that ${R}_{i}^{2}$ is always ≤1 but ‘can be negative’. A value of ${R}_{i}^{2}=1$ indicates a perfect model (for channel/region *i*), ${R}_{i}^{2}=0$ indicates a model as good as the constant predictor, and ${R}_{i}^{2} < 0$ indicates a model worse than the constant predictor.

### Linear models provide maximum prediction accuracy with minimum computational complexity

In this work, we fit and compare several families of linear and nonlinear models, as described below (see Methods for details). We fit each family of models to the data for each participant, thereby finding the optimal model at the global or local level (if the corresponding optimization algorithm is convex or non-convex, respectively). We then compare the resulting best models in each family in terms of their cross-validated fit to held-out data of the same participant (see Methods). The most important ground for comparison is the accuracy of their fit, measured by ${R}_{i}^{2}$ according to equation (3).

**Fig. 2:**
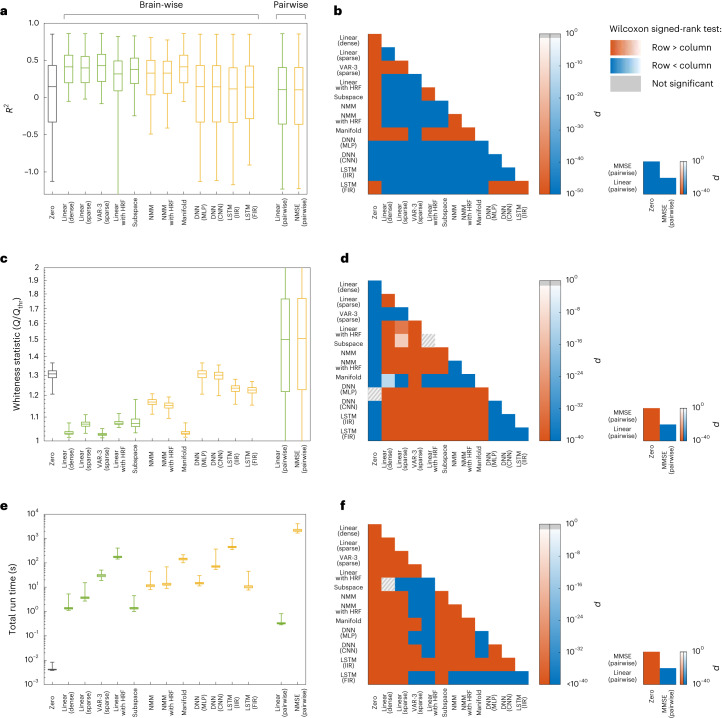
Linear vs nonlinear models of rsfMRI activity. **a**, The distribution of cross-validated regional ${R}_{i}^{2}$, combined across all 116 regions and 700 participants, for linear (green) and nonlinear (yellow) models. The grey box corresponds to the zero model used as a baseline (see Methods for an explanation of each model). Negative values of *R*^2^ indicate that models have a worse prediction performance than a constant predictor that always predicts the next value of a signal to be equal to the signal’s mean. Note the higher accuracy of the best linear model (‘VAR-3 (sparse)’) compared with all nonlinear ones. **b**, The *P* value of one-sided Wilcoxon signed-rank test performed between all pairs of brain-wise distributions (left) and all pairs of pairwise distributions (right) of *R*^2^ in **a**. Warm (cold) colours indicate that the distribution labelled on the row has significantly larger (smaller) samples than the distribution labelled on the column. Grey hatches indicate non-significant differences at an *α* = 0.05 with Benjamini-Hochberg False Discovery Rate (BH-FDR) correction for multiple comparisons. **c**,**d**, Similar to **a** and **b** but for the statistic *Q* of the multivariate test of whiteness relative to its rejection threshold *Q*_thr_ (cf. Methods). Smaller *Q*/*Q*_thr_ indicates whiter (better) residuals, with *Q*/*Q*_thr_ ≤ 1 required for the null hypothesis of whiteness not to be rejected. **e**,**f**, Similar to **a** and **b** but for the time that it took for the learning and out-of-sample prediction of each model to run, per participant per cross-validation (see Methods). In all boxplots, the centre line, box limits and whiskers represent the median, upper and lower quartiles, and the smallest and largest samples, respectively. VAR, vector autoregressive; HRF, haemodynamic response function; NMM, neural mass model; DNN, deep neural network; MLP, multilayer perceptron; CNN, convolutional neural network; LSTM, long short-term memory; IIR, infinite impulse response; FIR, finite-impulse response; MMSE, minimum mean squared error.

First, consider the results for the fMRI data (Fig. 2a,b). While we describe the results obtained using a relatively coarse parcellation here, similar results also hold for finely parcellated and unparcellated data (cf. Supplementary Figs. 12–14). Overall, linear models that directly fit the BOLD time series without (de)convolving with a haemodynamic response function (HRF), either with dense or sparse effective connectivity, and with or without higher-order AR lags, achieve the highest *R*^2^. Among nonlinear models, the manifold-based locally linear model achieves a comparable *R*^2^. Yet, upon closer inspection of this model, we observe that its window size (which is chosen optimally, see Methods and Supplementary Fig. 7) is very large, effectively making it a globally linear model. The lack of nonlinearity becomes even clearer when examining the pairwise models. Here we see that a simple linear model performs as well as the minimum mean squared error (MMSE) model, or even slightly better (Fig. 2b right panel) due to the numerical errors of distribution estimation. We thus infer that the former achieves the highest prediction accuracy achievable by ‘any’ generally nonlinear model, albeit for pairwise prediction.

The second ground for comparison is the whiteness of model residuals, also in held-out data, which indicates that all the dynamics in the data are captured by the model and have not leaked into the residuals (Fig. 2c,d). Here, linear models also score higher than nonlinear models, with autoregressive (AR) models clearly outperforming others. However, it is noteworthy that the null hypothesis of whiteness is rejected for the residuals of all methods (*Q*/*Q*_thr_ > 1), suggesting the presence of unexplained variance left by all models. Generally, the number of lags and sparsity patterns have little effect on the prediction accuracy of linear AR models for rsfMRI data, a positive but weak effect on the whiteness of the residual and a negative effect on the computational complexity (Supplementary Fig. 2). Similar to the comparison of *R*^2^ values, the only nonlinear model whose whiteness of residuals is comparable to the linear ones is the manifold-based locally linear model which, as explained above, is effectively linear at the global scale. Also as before, the pairwise linear models achieve a degree of whiteness that is almost identical to the pairwise MMSE estimator, ensuring their optimality among all linear and nonlinear pairwise predictors.

Third and finally, we can compare the models by considering the total time that it takes for their learning and prediction (Fig. 2e). When comparing the most efficient linear and nonlinear models, we find that linear models take at least one order of magnitude less time to fit than nonlinear models, as expected. However, linear methods can also be extremely complex to learn; linear models with states at the neural level (‘Linear with HRF’) require the most time to learn due to their high flexibility. Notably, this additional complexity of the ‘Linear with HRF’ or nonlinear methods is not counterbalanced by any benefits in their accuracy or whiteness of residuals, making the simplest linear models the preferred choice across all measures.

**Fig. 3:**
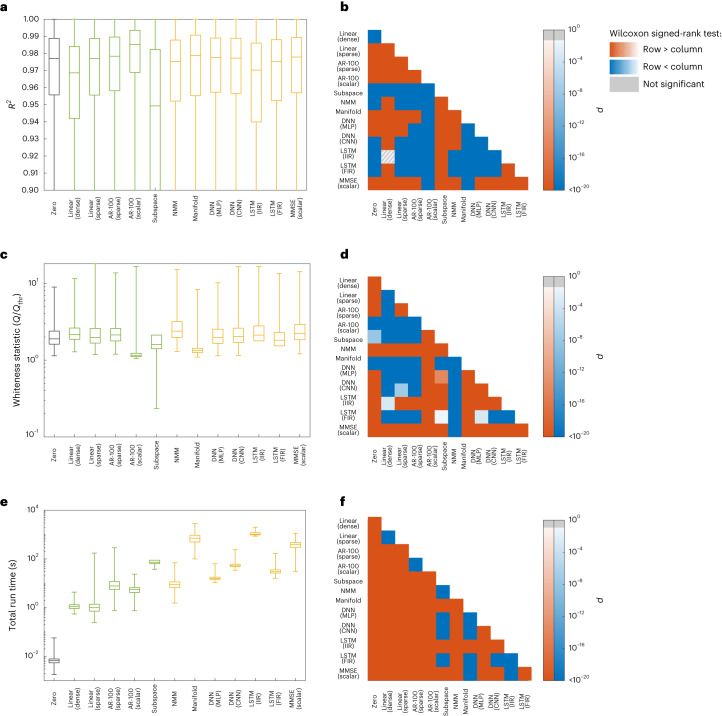
Linear vs nonlinear models of rsiEEG activity. Panels and acronyms parallel those in Fig. 2. **a**, The distribution of cross-validated regional ${R}_{i}^{2}$, combined across all electrodes (the number of which varies among participants) and all the recording sessions of the 122 participants (sample size = 776,484). Linear and nonlinear methods are depicted by green and yellow boxes, respectively (see Methods for an explanation of each model). Unlike data presented in Fig. 2, pairwise linear or pairwise MMSE models are not included due to the observation that between-electrode connections decrease the cross-validated accuracy of the top model (cf. the 4th and 5th boxplots). In contrast, including scalar autoregressive lags is highly beneficial in iEEG, whereas it is not so in rsfMRI. Therefore, the MMSE model here is scalar, conditioning on the past lags of each region itself. The lower whisker of the boxplots are trimmed to allow for better illustration of the interquartile ranges. **b**, The *P* value of the one-sided Wilcoxon signed-rank test performed between all pairs of distributions of *R*^2^ in **a**. Warm (cold) colours indicate that the distribution labelled on the row is significantly larger (smaller) than the distribution labelled on the column. Grey hatches indicate non-significant differences evaluated at *α* = 0.05 with BH-FDR correction for multiple comparisons. **c**,**d**, Similar to **a** and **b** but for the statistic *Q* of the multivariate test of whiteness relative to its rejection threshold *Q*_thr_ (cf. Methods). Smaller *Q*/*Q*_thr_ indicates whiter (better) residuals, with *Q*/*Q*_thr_ ≤ 1 required for the null hypothesis of whiteness not to be rejected. **e**,**f**, Similar to **a** and **b** but for the time that it took for the learning and out-of-sample prediction of each model. In all boxplots, the centre line, box limits and whiskers represent the median, upper and lower quartiles, and the smallest and largest samples, respectively.

Next, we perform the same comparisons between linear and nonlinear models, but now on the basis of their fit to resting-state iEEG field potential dynamics (Fig. 3). Similar to rsfMRI data, linear AR models provide the best fit to the data in terms of both the magnitude and whiteness of their cross-validated prediction error. These models also have lower computational complexity than nonlinear ones, with about an order of magnitude (or higher) advantage in computation time.

Alongside these similarities between the rsfMRI and rsiEEG data, two major distinctions are notable. First, the *R*^2^ values are generally much higher for iEEG, as evidenced by the *R*^2^ distributions of the zero model between the two cases. This difference is due to the fact that the iEEG time series has a much higher sampling rate and is therefore smoother. As a result, even predicting each sample equal to its previous sample (that is, the zero model) has a median accuracy of more than 97% (see Supplementary Figs. 17–19 for a more detailed assessment of the effects of sampling rate on models’ *R*^2^). This fact only highlights the importance of the zero model; without it, the *R*^2^ of all models might have seemed satisfactorily high. In comparison to the zero model, however, it becomes clear that a simple 1-lag linear model, for example, has in fact a very low predictive power.

The second major distinction between the two modalities is the amount of history and temporal dependency within them. fMRI data are almost Markovian, so that **y**(*t* − 1) contains almost all the information available for the prediction of **y**(*t*). Little information is also contained in **y**(*t* − 2), but almost no information is contained in timepoints further in the past (Fig. 2a). When considering iEEG data, in contrast, increasing the number of autoregressive lags up to ∼100 still improves the *R*^2^, although the exact optimal number of lags varies between data segments. In this comparison, it is also important to take into account the vast difference in the sampling frequencies between the modalities, where 2 lags in the fMRI dataset amount to 1.44 s, while 100 lags of the iEEG data sum to only 0.2 s. This greater ‘richness’ of iEEG dynamics from a modelling perspective is also responsible, at least in part, for the markedly lower whiteness of residuals of all model families with respect to fMRI (see Fig. 3c vs Fig. 2c). This greater richness of iEEG is also consistent with, although not necessarily a direct consequence of, the fact that iEEG data reflect neural signals more directly than fMRI.

### The linearizing effects of macroscopic neurodynamics and neuroimaging explain the observed linearity

The above results pose the natural question of why nonlinear models were not able to capture the dynamics in rsfMRI/rsiEEG data beyond linear ones, even though microscopic neuronal dynamics are fundamentally nonlinear. Here we focus on four properties of macroscopic neurodynamics and neuroimaging, and show that, in principle, they either fundamentally counteract or apparently mask nonlinearities. Due to its unique position in neural modelling^11,12^, we will use the sigmoidal nonlinearity to illustrate these effects; we note, however, that the effects are otherwise applicable to other forms of nonlinearity.

**Fig. 4:**
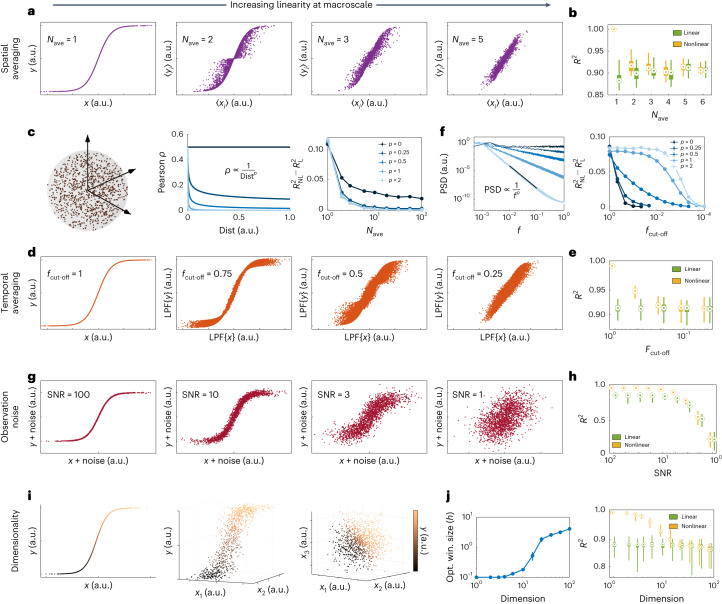
The linearizing properties of macroscopic brain dynamics and of neuroimaging measurements. **a**, The effect of spatial averaging. For each panel, *N*_ave_ pairs of signals *x*_*i*_(*t*), *t* = 1,…,2,000 were randomly and independently generated, ${y}_{i}(t)=\tanh ({x}_{i}(t))$ was calculated, and their averages 〈*x*_*i*_〉 and 〈*y*_*i*_〉 were computed. The quantities 〈*x*_*i*_〉 and 〈*y*_*i*_〉 possess a linear relationship as *N*_ave_ ≈ 5 or higher. **b**, The cross-validated *R*^2^ of the optimal nonlinear (MMSE) and linear predictors for the 〈*x*_*i*_〉–〈*y*_*i*_〉 relationships in **a**. **c**, The effect of spatial correlation on spatial averaging. Here we assign (*x*_*i*_(*t*),*y*_*i*_(*t*)) pairs to spatial locations in a unit sphere (left) and make each *x*_*i*_(*t*) and *x*_*j*_(*t*) correlated in a manner that depends on their spatial distance (middle). The difference between nonlinear and linear *R*^2^ always decays with *N*_ave_ and vanishes if the correlation decays, even slowly, with distance (right). **d**, The effect of temporal averaging. One pair of $x(t),y(t)=\tanh (x(t))$ was generated, independently over time and passed through a Gaussian low-pass filter (LPF) with a cut-off frequency *f*_cut-off_ that is normalized to the Nyquist frequency; thus, *f*_cut-off_ = 1 means no LPF. **e**, Same as **b** but for the LPF{*x*}–LPF{*y*} relationships in **d**. **f**, Similar to **c** but for temporal averaging. We varied the PSD decay rate of *x*(*t*) (left) and then low-pass filtered *x*(*t*) and $y(t)=\tanh (x(t))$ as in **d**. The difference between the optimal linear and nonlinear *R*^2^ eventually vanishes as *f*_cut-off_ decreases, but it happens at smaller *f*_cut-off_ for larger decay rates *p* (right). **g**, The effect of observation SNR. The quantities *x*(*t*) and and *y*(*t*) = tanh(*x*(*t*)) are as in **d** and their additive noises were generated independently. **h**, Same as **e** but for the (*x* + noise) − (*y* + noise) relationships shown in **g**. **i**, The effect of dimensionality. The values *x*_1_(*t*),…,*x*_*n*_(*t*) were generated as in **a** but here *y*(*t*) = tanh(*x*_1_(*t*)…+*x*_n_(*t*)) generates a one-dimensional nonlinearity in *n* + 1 dimensions. No noise is included; no spatial or temporal averaging is applied. **j**, Right: similar to **b**, **e** and **h** except that a manifold-based (locally linear) nonlinear predictor was used since the conditional density estimation required for MMSE loses accuracy in high dimensions with a fixed number of data points (see Methods). Left: the optimal window size of the manifold-based predictor as a function of dimension *n*. As *n* increases, the locally linear predictor automatically chooses larger windows to be able to make reliable predictions, thereby effectively degrading to a globally linear predictor (see also Supplementary Fig. 1). In all boxplots, the centre point, box limits and whiskers represent the median, upper and lower quartiles, and the smallest and largest samples, respectively. Error bars in **c**, **f** and **j** represent 1 s.e.m.

The first property that can fundamentally ‘counteract’ microscopic nonlinearities is spatial averaging. Imaging tools that are capable of measuring macroscopic brain dynamics detect a signal that reflects an average over the activity of hundreds, thousands or even millions of neurons. This spatial averaging can weaken, rather quickly, the nonlinear relationships in the dynamics of individual units (neurons or small-scale neuronal populations) as long as the units are not perfectly correlated, and can completely nullify nonlinearities when correlations decay with distance (Fig. 4a–c). Note that this distance can be the physical distance between the units, as assumed here, or in any relevant space such as that of neural codes and stimulus preference. The key factor in the linearizing effect of spatial averaging is the decay of pairwise correlations between neurons so that not all pairs of neurons in a region are highly correlated (a state of blanket global synchrony).

This linearizing effect of spatial averaging is similar to, but different from, stochastic linearization (also known as quasi-linearization)^26^. While the latter ‘approximates’ the relationship *y* = *σ*(*x*) using its expected slope *E*[∂*y*/∂*x*], spatial averaging as discussed here can result in a relationship that is truly linear. Also, the same effect can be observed when averaging other forms of nonlinearity than the sigmoid. Extended Data Fig. 2 shows the effect of spatial averaging on spiking neurons evolving according to the Izhikevic model^23^. This model has completely different nonlinearities than the sigmoid (polynomial and discontinuous) and shows a robust nonlinear phenomenon (limit cycle). Although more than a few (but still no more than 100–10^4^) neurons are required, spatial averaging still dissolves the nonlinear aspects of the dynamics while mostly sparing the linear ones.

The second property capable of completely counteracting microscale nonlinearities is temporal averaging. Macroscopic neural dynamics are often observed, or even defined, through signals that are low-pass-filtered versions of micro- and mesoscale variables. The most notable of these is perhaps the BOLD signal captured by fMRI, which can be seen as an observation of neural activity passed through the low-pass filter of the HRF. Similarly, although to a lesser extent, the local field potentials captured by iEEG most strongly reflect the aggregate pyramidal post-synaptic currents^27^, which are themselves low-pass-filtered observations of spiking activity through synaptic transmission and neuronal membranes’ resistive-capacitive circuit^28^. The effect of low-pass filtering, in essence, is temporal averaging, which impacts nonlinearities in a manner that is similar to that of spatial averaging (Fig. 4e–f). The parallel of spatial correlations here is the autocorrelation function or its frequency-domain representation, the power spectral density (PSD). Autocorrelation represents how the correlation between adjacent samples of a signal decay with the temporal distance between those samples. As expected, the smaller the bandwidth of the signal (that is, the faster their PSD decays with frequency before low-pass filtering), the weaker the linearizing effect of low-pass filtering. As a result, stronger low-pass filtering would also be required to completely nullify nonlinear relationships in signals with narrower bandwidth (Fig. 4d). The linearizing effect of temporal averaging also holds for deterministic dynamics, albeit with the resulting linear dynamics (post averaging) also being deterministic (Extended Data Fig. 3).

A third property that can counteract or mask nonlinearities is noise. Although both process noise and observation (scanner or electrode) noise may have linearizing effects, here we focus only on the latter. As with any neuroimaging time series, various sources of observation noise can affect the fMRI/iEEG time series^29,30^ and, in turn, ‘blur’ nonlinear relationships, even if they exist between the underlying noise-free BOLD/LFP time series (Fig. 4g,h). In fact, when the power of noise reaches the power of the signal (signal to noise ratio (SNR) ≈ 1), it can completely mask a nonlinear relationship in the absence of any spatial or temporal averaging. In reality, however, the linearizing effect of observation noise can combine with spatiotemporal averaging, making the 2 ≲ SNR ≲ 14 that we have in rsfMRI data (Supplementary Fig. 4) potentially more than enough to mask any remaining nonlinearities post-spatiotemporal averaging. Ironically, the use of linear filtering to ‘clean the data’ is more likely to further linearize the dynamics of the time series due to temporal averaging effects discussed above, instead of recovering nonlinearities lost due to noise (Supplementary Note 1). Nonlinear post-processing steps, on the other hand, may leave their own potentially nonlinear signatures in the data, but such signatures should not be confused with true nonlinear relationships in the original BOLD/LFP signal. Further, although we let the noise in Fig. 4g,h be independent of the signal, as is typically the case for measurement noise, this linearizing effect would still hold if the noise is linearly dependent on the signal.

The fourth and final property that we discuss is the number of samples required for detecting nonlinear relationships in large dimensions. Let us assume, despite our discussion so far, that a perfect noise-free nonlinear relationship exists between *n*-dimensional fMRI or iEEG time series and a noise-free sensor can capture it perfectly. When only *N* ≈ 1,000 data points are available, we find that the manifold-based predictor, which was our most predictive nonlinear method both for fMRI and iEEG, is still unable to predict the nonlinear relationship better than a linear model in *n* ≈ 40 dimensions or higher (Fig. 4i,j). This loss in the predictive power of this nonlinear predictor with increasing dimensionality can be easily seen from the fact that the smallest mesh, having two points per dimension, requires an exponentially large *N* = 2^*n*^ data points. Indeed, incorporating structural bias into the learning algorithm can arbitrarily reduce this sample complexity ‘if’ the incorporated bias is consistent with the underlying data^31^ (for example, if one looks for relationships of the form *y* = *σ*(*x*_1_ + ⋯ + *x*_*n*_) in Fig. 4i,j). However, using predictors with structural bias can also be arbitrarily misleading if their form of nonlinearity is not consistent with the given data^32^, which is one potential reason for the lower performance of most nonlinear methods in Figs. 2 and 3. This discussion also makes it clear that the inability of our nonlinear system identification methods to outperform linear ones in Figs. 2 and 3 over the entire brain is not a proof that no nonlinear method can possibly do so. We can, nevertheless, be certain about this for pairwise or scalar AR models (for fMRI and iEEG, respectively) where the optimal MMSE predictor was computable and performed as well as a linear one.

In conclusion, the process of averaging over space, the process of averaging over time, the existence of observation noise and the acquisition of limited data are each characteristic of macroscale brain dynamics or neuroimaging measurements and can transform microscopically nonlinear dynamics into macroscopically linear ones. In reality, their effects are probably all combined, rendering the optimality of linear models in our comparisons not as unexpected as it might originally seem. This linearity has major implications for computational neuroscience, as we discuss next.

## Discussion

### Summary

In this work, we set out to test the hypothesis that macroscopic neural dynamics are nonlinear, and using linear models for them results in an inevitable loss of accuracy in exchange for simplicity. We thus compared linear and nonlinear models in terms of how well they can predict rsfMRI and rsiEEG data in a cross-validated PE system identification framework, where the quality of each model’s fit was assessed by the variance and whiteness of its PE (residual). We found that linear models, and AR models in particular, achieve the lowest PE variance and highest PE whiteness, outperforming neural mass models (NMMs), deep neural networks (DNNs), manifold-based models and the optimal MMSE predictors. In the case of fMRI data, we further verified that the higher predictive power of AR models holds not only in aggregate but also in a strong majority of individual regions across the brain (Extended Data Fig. 4a). Interestingly, the spatial (regional) distribution of the *R*^2^ of the best model also shows significant differences across established cortical functional networks, a remarkably lower predictability of subcortical regions relative to cortical ones and a close alignment between most methods (all except for ‘Linear with HRF’) (Extended Data Fig. 4b–d). This distinction in predictability highlights significant differences in how spatio-temporally correlated the fMRI time series of different regions are, while the mechanistic physiological and technological reasons behind this distinction remain a warranted avenue for future research.

To further understand the possible causes of the optimality of linear models, we analysed the effects of common elements of macroscopic neural dynamics: averaging over space and time, observation noise and limited data samples. We showed that they can each counteract or mask the nonlinearities present at smaller scales. These linearizing effects add up when combined, suggesting that linear models provide a useful choice for macroscopic neural dynamics at rest; of course, in certain experimental conditions, rigorous system identification methods might still uncover nonlinear dynamics in future studies.

The observed optimality of linear models for the resting state is accompanied by both challenges and opportunities. Having a linear model for neuroscience investigations is computationally ideal, given the extent to which the behaviour of linear systems and their response to stimuli are mapped out. Nevertheless, to what extent these linearly interacting macroscopic signals are informative of and have a causal influence on the underlying microscopic activity remains unclear and represents an invaluable area for future investigation. Our observations also warrant the exploration and development of both linear and nonlinear models of macroscopic neural dynamics beyond those tested here and available in the literature.

### Connections to previous literature

It is important to distinguish the pragmatic modelling question that drove our analysis from the rather philosophical question of whether any signs of ‘nonlinearity’ can be found in neuroimaging time series. The latter question has been extensively investigated^17–19,33^ and often uses determinism or chaos as a proxy for nonlinearity. To answer our distinct modelling question, we used a system identification approach that allows for a direct, side-by-side comparison of linear and nonlinear models. In contrast, the aforementioned studies often resorted to indirect, surrogate-based comparisons that rely on strong (and debated) assumptions about the constructed surrogates^34^. Also related to, but different from, our work are studies that seek to determine whether the end-to-end input–output mapping between stimuli and neuroimaging signals (EEG or BOLD) (refs. ^35,36^) or between functional connectivity and individual phenotypes^37,38^ is (highly) nonlinear. Our focus here, however, is on the nonlinearity of the internal network dynamics of the brain. Finally, past studies have also examined the performance of linear models per se in fitting neuroimaging time series (for example, ref. ^39^) but without a comparison to nonlinear models.

Our analysis of the linearizing effect of spatial correlation is also related to the large body of work investigating the effect of spatial correlations on the information content and decoding accuracy of neural population codes (see for example, ref. ^40^). As expected, the stronger the correlation between neurons, the weaker the linearizing effects of spatial averaging. However, nonlinearities can in principle have two opposing effects on the neural code. On the one hand, nonlinearities can substantially increase the computational complexity and expressivity of a neural network, making correlations beneficial for the neuronal encoding. On the other hand, if the expressivity is too high, the decodability of one neural population by another may decrease, potentially making the linearizing effects of low correlations favourable. Determining which effect dominates and whether an optimal point exists at the levels of neural correlation observed remain areas of future research in vivo.

### Results and implications

The implications of the linearity of brain dynamics are far-reaching. Linear systems fundamentally have a more limited repertoire of dynamic behaviours than nonlinear ones, excluding the possibility of multistability, chaos, limit cycles or cross-frequency coupling, to name a few^41^. When driven by noise, linear systems act as linear filters that shape the power spectrum of their output (here, fMRI or iEEG time series) through their frequency response, essentially amplifying the frequency content near their resonance frequencies and dampening it elsewhere. Importantly, this effect of shaping the power spectrum of linear systems acts independently over different frequencies; in contrast, nonlinear systems can drive arbitrarily complex cross-frequency interactions^42^.

The linearity of brain dynamics has even greater implications for network control^43,44^. The design and analysis of optimal, robust, adaptive and many other forms of control are much better understood in the context of linear systems than nonlinear ones. This contrast in tractability only grows for large-scale systems such as the brain, thus motivating the recent surge of interest and advancements in using linear control theory in neuroscience^45–47^. Nonlinear models also present additional challenges beyond network control, including analytical and mechanistic understanding of their functionality, obtaining provable guarantees on their performance and even hardware requirements for their use in chronic implantable devices. In this context, the present work shows that the favourable tractability and simplicity of linear models do not necessarily come at the often-presumed cost of model inaccuracy and also provide the necessary tools for identifying the most accurate models for any datasets of interest.

In the analysis of fMRI data, we found that incorporating an HRF component in the model, instead of modelling the dynamics directly at the BOLD level, results in a loss of accuracy in linear models (see ‘Linear (sparse)’ vs ‘Linear with HRF’), and is almost ineffective in nonlinear models (see ‘NMM’ vs ‘NMM with HRF’). It was also in light of this observation that we did not include an HRF component in the majority of our models, such as the DNN or the manifold-based models. This lack of advantage of an explicit HRF component (within the specific context of modelling resting-state fMRI dynamics using ODEs) is understandable on a number of grounds. First, to include an HRF component in the model, one should either learn the HRF from the data, such as in our ‘linear with HRF’ model, which creates marked model flexibility and therefore increases the likelihood of overfitting, or use a typical HRF, such as in our ‘NMM with HRF’ model, which is a source of additional error. Second, by including the HRF in the model, we ultimately seek to recover neural information that is lost through the HRF. This task is difficult, if not impossible, without a high signal-to-noise ratio as well as more accurate HRF models than those currently available. Finally, a linear autoregressive model can automatically capture a linear approximation of the HRF dynamics^48^, precisely as present in the observed time series. Ultimately, our results encourage a side-by-side comparison of models with and without the inclusion of an HRF component to assess the costs and benefits of such inclusion for any datasets of interest.

A very similar argument also applies to including coloured noise in the model, both for fMRI and iEEG. Even though the so-called ‘noise floor’ in neural signals over which neural oscillations are detected^49^ has a clear power-law (1/f) PSD, its decay can be well modelled by white noise passing through a linear filter. This property of the 1/f noise is in fact why the AR models, which assume a white noise signal **e**(*t*), have prediction errors that are maximally white. This latter fact can be directly seen from Figs. 2c and 3c, when noting that any model’s prediction errors are, by construction, the model’s estimate of the noise **e**(*t*) (ref. ^25^).

In addition to considering linear and nonlinear models, we underscore the importance of the zero model. It is not uncommon in the modelling literature to assess the quality of a fitted model per se, without any grounds for comparison. For instance, our DNN multilayer perceptron ‘DNN (MLP)’ model for fMRI had a median *R*^2^ of ∼14% and for some participants it had a median *R*^2^ (among all regions) of over 50%. Even more notably, the ‘DNN (MLP)’ model for iEEG had a median *R*^2^ of over 97%. Without any comparisons, these numbers may suggest that the ‘DNN (MLP)’ models are quite accurate; yet, as seen in Figs. 2b and 3b, the predictive accuracy of these models is in fact lower than that of the zero model in fMRI and indistinguishable from that of the zero model in iEEG. The act of comparing to a baseline model is therefore an essential step in the assessment of any model’s goodness of fit.

We restricted our analyses here in the main text to certain spatiotemporal resolutions for both fMRI (a coarse parcellation) and iEEG (a high sampling rate), naturally raising the question of how robust our findings are to our choices of resolutions. As shown in Supplementary Figs. 12–19, our main finding (higher predictive power of linear autoregressive models over all other model families) holds across all resolutions tested. We do, however, observe certain differences between resolutions. In iEEG data, we observe that using lower sampling rates (and therefore longer time intervals) increases the benefit of modelling network interactions, even while lowering the *R*^2^ values across all models. In fMRI data, we interestingly see that as we move towards more fine-grained parcellations and ultimately unparcellated data, (1) the simpler ‘Linear (sparse)’ model with less parameters gains advantage over the more populated ‘VAR-3 (sparse)’ model and (2) the overall *R*^2^ values of all models are reduced, potentially due to the improvement in signal-to-noise ratio resulting from averaging in coarser parcellations.

### Methodological considerations

Despite the solid theoretical foundations of the PE method for system identification, our results may still beg a practical question: would the same system identification and side-by-side comparison procedure be able to identify nonlinear dynamics, should they actually exist in the time series? A direct answer to this question can be given, for example, by applying the same procedure to simulated time series generated from a nonlinear model whose ground-truth functional form we know. The result of such an analysis is provided directly in Extended Data Figs. 2 and 3 but also indirectly in Fig. 4. Note that in the latter, we compared the cross-validated predictive power of linear and nonlinear (MMSE or manifold-based) models in identifying the sigmoidal relationship *y* = *σ*(*x*) and its variants after averaging or noise addition. This relationship can be equally viewed as a nonlinear dynamical system $\dot{x}=\sigma (x)$, the nonlinearity of which was only identified until counteracted or masked by the four macroscopic effects we discussed therein.

In Supplementary Fig. 9, we take this validation one step further and compare the ‘DNN (MLP)’ model with linear autoregressive and subspace models while also tuning their respective hyperparameters using simulated data from the Izhikevic model and the same stochastic gradient descent (SGD) algorithm used for hyperparameter tuning in the main results. The resulting higher predictive performance of the ‘DNN (MLP)’ model is another testament to the validity of the end-to-end process we used for model training and comparison. Note also that the amount of improvement that nonlinear models can offer over linear ones can vary widely on the basis of the true underlying dynamics and the ability of either model to capture them. In Supplementary Fig. 10, for example, we show a similar side-by-side comparison between the ‘DNN (MLP)’ and ‘Linear (dense)’ models on one of the simplest nonlinear systems exhibiting a nonlinear behaviour (chaos in this case). Despite the extreme simplicity of the true underlying model, the neural network can achieve near-perfect accuracy with only one hidden layer and 10 hidden units, while the linear model can achieve ∼50% *R*^2^. Finally, we also trained both the parameters and hyperparameters of the ‘DNN (MLP)’ model on fMRI data while replacing its rectified linear unit (ReLU) activation functions with linear ones using the same codes and routines used for the actual ‘DNN (MLP)’ model. As seen from Supplementary Fig. 11, the resulting model achieves indistinguishable *R*^2^ distributions from the (theoretically equivalent) ‘Linear (dense)’ model, except for implementation differences between our hard-coded linear regression solver and that provided by MATLAB’s ‘trainNetwork’ function.

As for the comparison of linear and nonlinear models, one might expect nonlinear models to perform at least as well as linear ones, but not worse, given that the space of nonlinear models includes all linear models as a special case. In our comparison, however, we saw that most of our nonlinear methods actually have a worse prediction performance than linear ones. This behaviour can be understood in light of at least two facts. First, many nonlinear models, such as NMMs, do not include linear models as a special case and have structural biases that can be a source of error if not consistent with the data^32^. Second, even nonlinear models that do not have structural biases and/or contain linear models as a special case, such as DNN, MMSE or manifold-based models, still have a marked flexibility relative to a linear model. This immense flexibility makes the training of these models and finding their global optimum challenging. As a result, training algorithms are quite likely to return suboptimal models which, in this case, show worse accuracy and generalization than their linear special cases.

**Extended Data Fig. 1:**
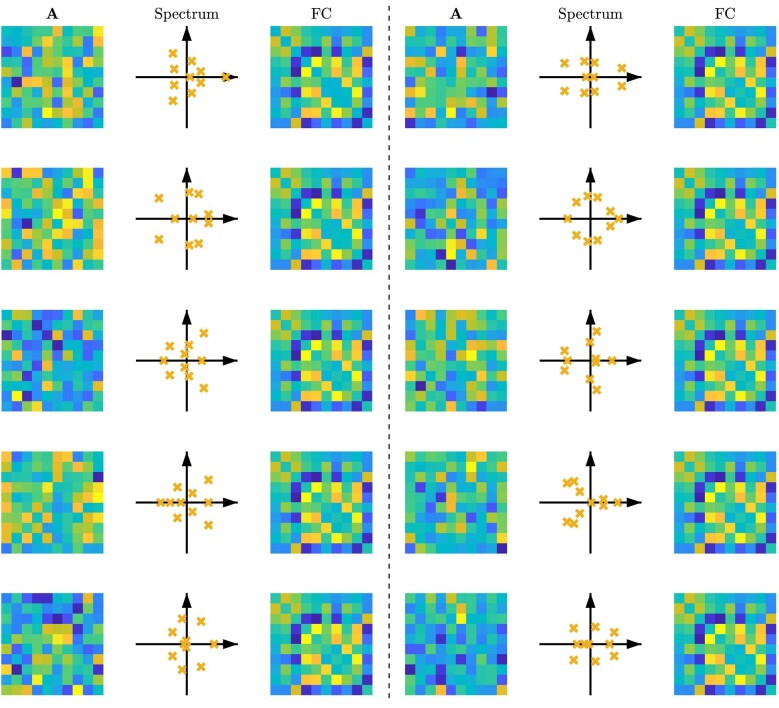
Different systems can produce almost identical functional connectivity (FC). Ten different systems of the form **y**(*t*) = **Ay**(*t* − 1) + **e**(*t*) are shown with completely different **A** matrices and spectra (eigenvalues of **A**), but almost identical FC matrices. The Pearson correlation coefficient between all pairs of FCs is 1.0000 while the absolute value of the Pearson correlation coefficients between the A matrices range from a minimum of 0.0023 to a maximum of 0.36. All systems were driven by independent and randomly generated noise sequences **e**(*t*). In the figures, the diagonal entries of the FC matrices (which are by definition equal to 1) are set 0 to increase color resolution.

**Extended Data Fig. 2:**
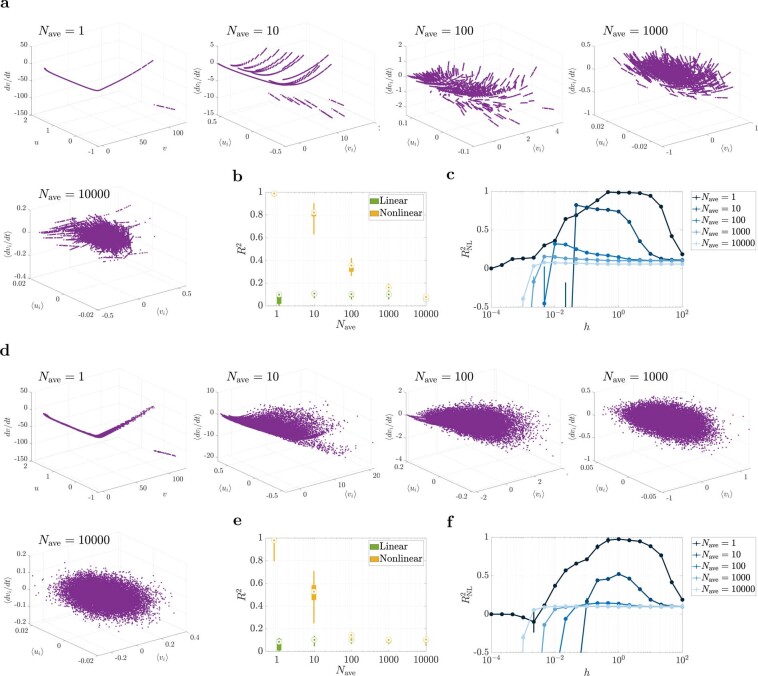
The linearizing effect of spatial averaging on the Izhikevic spiking model. (a) The Izhikevic model^23^ was simulated for *N*_ave_ neurons and their *v*_*i*_ and *u*_*i*_ variables were averaged before being ‘observed’. The neurons had the same parameters (and hence the same spiking frequency) but were initialized at random phases of their limit cycles. The average of *dv*_*i*_/*dt* is plotted against the average of *v*_*i*_ and *u*_*i*_, showing the loss of nonlinearity at about *N*_ave_ ~ 10^3^ − 10^4^. *dv*_*i*_/*dt* is chosen since it has a more nonlinear dependence on *v*_*i*_ and *u*_*i*_ than *du*_*i*_/*dt* for one neuron, but the same (even stronger) linearizing effect holds for (〈*v*_*i*_〉, 〈*u*_*i*_〉, 〈*du*_*i*_/*dt*〉). (b) The *R*^2^ distributions of a linear (simple linear regression) and nonlinear (manifold-based locally linear regression) model for the relationships in panel (a) and 100 random repetitions. The manifold-based model was chosen over the MMSE model as it consistently gave significantly higher *R*^2^ values (due to the high sample complexity of the MMSE model). Note that the *R*^2^ distribution of the linear model is mostly flat (less affected by spatial averaging), while that of the nonlinear model decays rapidly with *N*_ave_ until it reaches the linear level. (c) Nonlinear *R*^2^ as a function of the manifold method’s window sizes *h* for varying *N*_ave_ values. The circles and error bars show median and inter-quartile ranges, respectively. While an optimal, mid-range *h* exists for small values of *N*_ave_, the curve plateaus for large *h* as *N*_ave_ increases, showing the loss of nonlinearity with increasing *N*_ave_. (d-f) Same as panels (a-c) but for a model where process noise is added to the *dv*_*i*_/*dt* equations. The noise has a very low power (SNR = 100) and is only meant to destroy the artificial micro-nonlinear relationships that are visible in panel (a) and can inflate the power of nonlinear regression for *N*_ave_ ~ 10 − 10^3^. Note that this is different from the addition of observation noise discussed in the main text, even though adding observation noise could serve this purpose as well. In all box plots, the center point, box limits, and whiskers represent the median, upper and lower quartiles, and the smallest and largest samples, respectively.

**Extended Data Fig. 3:**
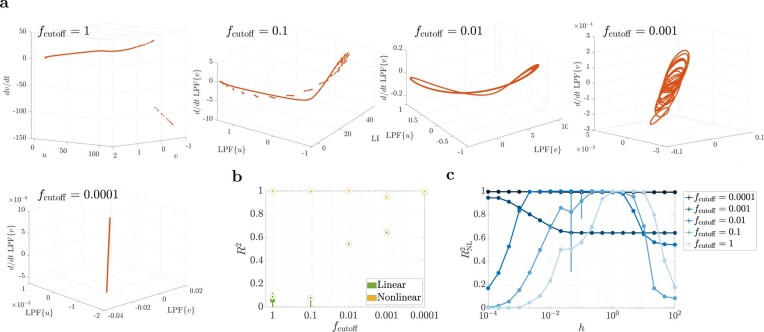
The linearizing effect of temporal averaging on the Izhikevic spiking model. Panels parallel those in Extended Data Fig. 2. (a) One Izhikevic model^23^ was simulated, starting from a random initial condition on its limit cycle, and its *v* and *u* variables were low-pass filtered before being ‘observed’. *d*/*dt* LPF{*v*} is plotted against LPF{*v*} and LPF{*u*}, showing the loss of nonlinearity at about *f*_cutoff_ ~ 10^−4^. (b,c) Similar to panels (b,c) in Extended Data Fig. 2, except that here the *R*^2^ of the linear model reaches that of the nonlinear one as temporal averaging is intensified. The nonlinear model always has a near-perfect prediction power given the fully deterministic nature of this simulation (that is, a sufficiently small *h* was always sufficient to perfectly match a locally-linear model to the curves in panel (a). Note that addition of process noise here does not have the same ‘blurring’ effect of Extended Data Fig. 2(d–f) since it lies before the low-pass filter. Observation noise can nevertheless be added with the same effect (not shown here). In all box plots, the center point, box limits, and whiskers represent the median, upper and lower quartiles, and the smallest and largest samples, respectively.

**Extended Data Fig. 4:**
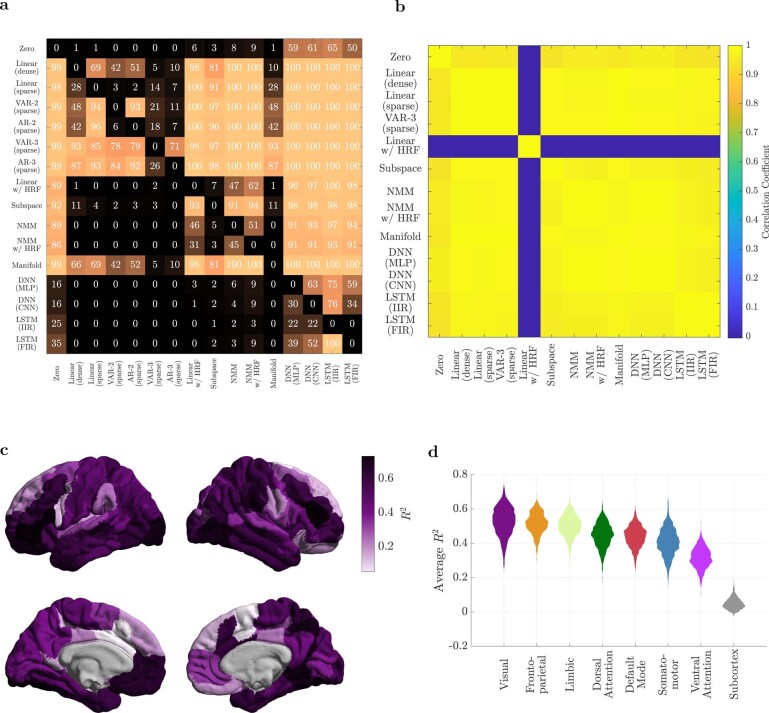
The spatial distribution of predictability in fMRI data. (a) Regionwise comparisons between all pairs of models. The color and number in each cell indicates the percentage of brain regions where the median of the *R*^2^ values of the model on the row is statistically significantly greater that the median of the *R*^2^ values of the model on the column (*α* = 0.05, Wilcoxon signed rank test, and Bonferroni correction for multiple comparisons). (b) The correlation coefficient between the *R*^2^ values of all the (brain-wise) methods. All methods produce almost the same cortical distributions of *R*^2^ (albeit with different absolute values of *R*^2^, cf. Fig. 2a in the main text), except for ‘Linear w/ HRF’ which has an un-correlated *R*^2^ distribution relative to the rest of the methods. (c) The cortical distribution of the *R*^2^ of our best model (‘VAR-3 (sparse)’), averaged over the 700 subjects. (d) Violin plots of the distribution of *R*^2^, averaged over all the regions of each resting state network, for all subjects. Note that each distribution in panel (b) is thus composed of 700 samples. All pairs of distributions have significantly different medians in the order plotted (one-sided Mann-Whitney U-test at *α* = 0.05 with BH-FDR correction for multiple comparisons). The most striking difference is between the cortical and subcortical regions, where the dynamics of the latter are remarkably less predictable than the former. This lower predictability can be viewed as having ‘more noisy’ dynamics or, more precisely, less spatially and temporally correlated (that is, more white) fMRI time series in these regions.

A further noteworthy aspect of our study specifically, and the prediction error framework more generally, is the focus on fitting the time series rather than its derivative statistics, such as the functional connectivity (FC) (refs. ^50–52^) or power spectral density^53^. While the choice of one approach over the other ultimately depends upon the anticipated use of the learned model, it is important to note that the mapping from dynamical systems to FC (or any other such statistic) is not a one-to-one mapping^54^. In fact, linear systems of the form **y**(*t*) − **y**(*t* − 1) = *W***y**(*t* − 1) + **e**(*t*) with completely different *W* matrices can give rise to almost identical FC matrices (see Extended Data Fig. 1). Therefore, when considering the accuracy of a general-purpose model of the brain, the time series contains the maximum amount of information and thus provides the best target for model fitting.

One modelling approach that we did not employ in this study is dynamic causal modelling (DCM, ref. ^55^). The reason is that none of the current variants of DCM are feasible due to their computational complexity at the scale of our analysis: whole-brain fMRI with *n* = 116 parcellations or large-scale iEEG with up to 175 and a median of 98 electrodes. The most efficient variant, spectral DCM for fMRI, for instance, is applicable to ∼30–40 nodes, whereas stochastic DCM (the most relevant to our study) is only applicable to much smaller systems. However, in light of our results thus far, the great computational complexity of the DCM approach and thus its potential for overfitting, we would not expect its cross-validated *R*^2^ to reach that of a linear model, although this comparison remains unknown at present.

In this work, we demonstrated four properties of macroscopic neurodynamics that can counteract or mask microscopic nonlinearity. In doing so, we purposefully kept the discussion at a conceptual level and generally abstained from tying it to specific micro- or mesoscopic neural models, as doing so would require building on assumptions that our study explicitly seeks to avoid. For instance, it is currently unclear whether and to what extent the dynamics of the ‘mesoscopic’ local field potentials or population firing rates that seem to be the main neural drivers of fMRI or iEEG are nonlinear and, if so, what the precise form of their nonlinearity is at each brain region. A warranted avenue for future research would be the re-analysis of the effects of spatial and temporal averaging, observation noise and limited data samples on precise, data-driven models of mesoscopic brain dynamics, should they possess nonlinear interactions.

### Limitations

Finally, we highlight some of the limitations of the present study. First, it is important to note that the space of all nonlinear models in tens to hundreds of dimensions is intractably large, and the fact that our tested nonlinear models did not outperform linear ones is not a proof that no nonlinear model may ever do so.

In particular, some nonlinear models (for example, ‘DNN (MLP)’) have the capacity to implement the ‘Zero’ or even linear AR models as a special case (for example, by setting the output weights of the ‘DNN (MLP)’ model to zero to replicate the ‘Zero’ model or to adjust its weights and biases so that all ReLU activation functions operate in their linear range to replicate the ‘Linear (dense)’ model). Therefore, despite our best efforts in training these models and validating our training algorithms in alternative simulated settings (cf. Supplementary Figs. 7–9), the fact that their predictive power does not reach the performance of the zero/linear models in some datasets suggests suboptimal parameter and/or hyperparameter fitting due, for example, to local minima or a lack of enough training data. Therefore, we should highlight that our achieved predictive power for those nonlinear methods does not necessarily reflect their best performance and it is possible that future work with more training data and/or alternative optimization approaches may get better results from these nonlinear models that match or outperform linear models in iEEG/fMRI.

Our work thus seeks to provide rigorous evidence and methodology towards resolving the linear vs nonlinear modelling dilemma in computational neuroscience, rather than a final resolution thereof. Hybrid models consisting of various combinations of different nonlinear structures (LSTMs and CNNs, for example) may have substantial potential for obtaining more accurate nonlinear models and warrant future research. We can be confident, nevertheless, about the optimality of linear models at the pairwise level for fMRI or scalar AR level for iEEG given the equal or higher prediction power of linear regression relative to the optimal MMSE predictor. Moreover, our modelling framework is currently only applicable to resting-state dynamics with no inputs and has been tested on the two modalities of fMRI and iEEG. Inclusion of input signals for system identification of task fMRI/iEEG data requires accurate data-driven ‘input models’ of how experimental stimuli, as well as participants’ voluntary responses, influence the BOLD or LFP signals in each brain region and is a highly warranted avenue for future research^56,57^. Under intensive task conditions, moreover, it is more likely, or perhaps certain, to observe nonlinearities at least in the form of saturation effects in the BOLD/LFP signal. However, the precise form and extent of this nonlinearity need to be determined using rigorous system identification routines.

In conclusion, our work sought to ask the often-unasked question of whether the brain is macroscopically linear. Our findings show that simple linear models explain the rsfMRI and rsiEEG data as well as, or even better than, an array of nonlinear ones, thus challenging the commonly held yet untested assumption of higher accuracy of nonlinear models. However, the costs and benefits of nonlinear models are ultimately case-specific. Therefore, instead of offering a universal recommendation on the preferable choices for the modelling of neural dynamics, we rather provide the groundwork for rigorous investigation and informed decision-making in the context of rsfMRI/rsiEEG. When feasible, following a similar system identification routine is always recommended for computational modelling of any datasets of interest to ensure the optimal fit of the models used for subsequent analysis or design.

## Methods

### Data and preprocessing

For the fMRI analysis, we used ICA-FIX resting-state data from the S1200 Human Connectome Project (HCP) release^58,59^. The HCP experiments were carried out by the WU-Minn consortium and its adherence to ethical standards was approved by the Internal Review Board of the respective institutions. Explicit informed consent was acquired from all participants involved in the study. rsfMRI images were collected with the following parameters: TR = 20 ms, TE = 33.1 ms, flip angle = 52°, FOV = 208 × 108 mm, matrix = 104 × 90, slice thickness = 2.0 mm, number of slices = 72 (2.0 mm isotropic), multifactor band = 8 and echo spacing = 0.58 ms. Brains were normalized to fslr32k via the MSM-AII registration and global signal was removed. No bandpass filtering was performed (see Supplementary Note 1). Finally, we removed participants from further analysis if any of their four resting-state scans had excessively large head motion, defined by having frames with >0.2 mm frame-wise displacement or a derivative root mean square above 75. Also, participants listed in ref. ^60^ under ‘3T Functional Preprocessing Error of all 3T RL fMRI runs in 25 Subjects’ or ‘Subjects without Field Maps for Structural scans’ were removed, leaving a total of 700 participants that were used for all the analyses. We parcellated the brain into 100 cortical regions (Schaefer 100 × 7 atlas^61^) and 16 subcortical ones (Melbourne Scale I atlas^62^).

For iEEG preprocessing, raw data from the Restoring Active Memory (RAM) dataset we have previously published^63–65^ were segmented into task-free epochs from either before or after task completion that were at least 5 min in length. This process resulted in a total of 283 recordings from 122 participants. Data were then downsampled to the lowest sampling rate used across recording sites (500 Hz). Electric line noise and its harmonics at 60, 120 and 180 Hz were filtered out using a zero-phase distortion 4th order stop-band Butterworth filter with a 1 Hz width. This procedure was implemented using the ‘butter()’ and ‘filtfilt()’ functions in MATLAB. We then rejected noisy channels that were either (1) marked as noisy in the RAM dataset notes, (2) had a line length greater than three times the mean, (3) had *z*-scored kurtosis >1.5 or (4) had a *z*-scored power spectral density dissimilarity measure >1.5. The dissimilarity measure used was the average of one minus the Spearman’s rank correlation with all channels. Data were then demeaned and detrended. Channels were grouped according to whether they were grid or depth electrodes and then common average referenced within each group. Following the common average referencing step, plots of raw data and power spectral densities were visually inspected by an expert researcher with 6 yr of experience working with electrocorticography data to ensure that data were relatively clean.

Finally, while the aforementioned channel removal criteria are consistent with the standard practice in iEEG preprocessing (where we remove channels with exceptional line length, kurtosis and power spectral densities to target high-frequency noise and ictal activity^66,67^, electrode drift and ictal spikes^68–70^, or line noise and flat power spectral densities^65,71^, respectively) and they are essential from a data quality perspective, see also Supplementary Fig. 22. Here we reproduced our main finding of the higher predictive power of linear models on data without channel removal as a validation that channel removal had not confounded our finding by potentially providing an edge for the linear models.

### Computing and run-time calculations

All the computations whose run times were measured and reported in Figs. 2e and 3e were performed on the CUBIC cluster at the University of Pennsylvania, using a 1 CPU core and 16 or 64 GB of memory per fMRI or iEEG computing jobs, respectively. For each method, fMRI participant/iEEG segment and cross-validation fold, one training time, one test time and one total time were computed, where the latter is simply the sum of the former two. Note that for the ‘Zero’, ‘Manifold’ and ‘MMSE’ models, no training time could be defined. This should be clear for the ‘Zero’ model, but it was also the case for the ‘Manifold’ and the ‘MMSE’ models due to their ‘model on demand’ nature, that is, that all training data were directly used in computing the prediction of each test point, instead of the usual process of learning one model from the training data and then using that model for test predictions. Therefore, the training time was set to zero for these models. For the remaining models, the training time equalled the CPU time taken for all the computations, from the point when data had been broken into training and test sets until the point when the model was learned. The test time, well-defined and non-zero for all the methods, started immediately after the end of the training period and ran until the point when the output time series (one-step-ahead predictions) were computed. Note that computations of the *R*^2^ and whiteness statistics were optional post-hoc analyses and were done in the same way for all methods, hence were not included in any of these run-time calculations. A breakdown of the run time into separate training and test times is also shown in Supplementary Figs. 5 and 6.

### Linear and nonlinear families of models

A complete list of the models used in this study are provided in Supplementary Table 1. For all models, the continuous-time dynamics in equation (1) was first discretized. With a slight abuse of notation, we also represent the discretized dynamics as

4a $${{{\bf{x}}}}(t)-{{{\bf{x}}}}(t-1)=f({{{\bf{x}}}}(t-1))+{{{{\bf{e}}}}}_{1}(t),$$

4b $$\begin{array}{rcl}{{{\bf{x}}}}(0)&=&{{{{\bf{x}}}}}_{0},\qquad t=1,\ldots ,N\\ {{{\bf{y}}}}(t)&=&h({{{\bf{x}}}}(t))+{{{{\bf{e}}}}}_{2}(t),\qquad t=0,\ldots ,N\end{array}$$

where the time index *t* is now an integer, for simplicity of notation, but the discretization step size is always equal to 720 ms for the HCP data (equivalent to 1 TR) and 2 ms for the RAM data. This choice means that, for example, the map *f* in equation (4a) equals 1 time step multiplied by the map *f* in equation (1a), and *T* = 864 s in equation (1a) corresponds to *N* = 1,200 in equation (4a) for the fMRI data. Recall that in this general form, the noise signals **e**_1_(*t*) and **e**_2_(*t*) can have arbitrary statistics, including white or coloured PSD. We then learned the dynamics in equation (4) using the following families of models. The hyperparameters used for each model are listed in Supplementary Table 1 (see ‘Hyperparameter selection’ below and Supplementary Figs. 7 and 8 for details).

#### Linear models with states at the BOLD/LFP level (‘Linear (dense)’, ‘Linear (sparse)’)

This model is our simplest. In it, we let **y**(*t*) = **x**(*t*), modelling the dynamics directly at the BOLD/LFP level. This also allows for combining the noise signals **e**_1_(*t*) and **e**_2_(*t*) into a single noise signal **e**(*t*), which was then taken to be white. These simplify equation (4) to

5 $${{{\bf{y}}}}(t)-{{{\bf{y}}}}(t-1)=f({{{\bf{y}}}}(t-1))+{{{\bf{e}}}}(t).$$

If we further let *f*(**y**(*t*)) = *W***y**(*t*) be linear, then we get

6 $${{{\bf{y}}}}(t)-{{{\bf{y}}}}(t-1)={{{{W}}}}{{{\bf{y}}}}(t-1)+{{{\bf{e}}}}(t)$$

where *W* is an *n*-by-*n* matrix of effective connectivity between brain regions. We fit and compared this model both when *W* is dense and when it is sparse. The latter was motivated by the facts that (1) from a mechanistic perspective, an important property of brain networks and other large-scale complex networks is their sparsity; and (2) from a machine learning perspective, regularization and reducing the number of free parameters in a model can prevent overfitting and improve generalization. To promote sparsity, we used standard 1-norm (LASSO) regularization with a *λ* hyperparameter that is tuned separately for fMRI and iEEG.

#### Linear AR models (‘AR-2 (sparse)’, ‘VAR-2 (sparse)’, ‘AR-3 (sparse)’, ‘VAR-3 (sparse)’, ‘AR-100 (sparse)’, ‘AR-100 (scalar)’)

Motivated by the long history of AR models in neuroscience^24,72,73^, here we extend equation (6) to

7 $$\begin{array}{rcl}{{{\bf{y}}}}(t)-{{{\bf{y}}}}(t-1)&=&{{{{W}}}}{{{\bf{y}}}}(t-1)+{{{{{D}}}}}_{2}{{{\bf{y}}}}(t-2)\\ &&+{{{{{D}}}}}_{3}{{{\bf{y}}}}(t-3)+\cdots \\ &&+{{{{{D}}}}}_{d}{{{\bf{y}}}}(t-d)+{{{\bf{e}}}}(t)\end{array}$$

for an ‘AR-*d*’ model. The number of lags *d* was tuned separately for fMRI and iEEG, and the matrix *W* is either made sparse using LASSO or enforced to be diagonal. Note that the latter results in *n* scalar AR models at each node, which are completely decoupled from each other. We restricted the matrices *D*_2_, *D*_3_,… to be diagonal in ‘AR’ models but not so in full vector autoregressive (‘VAR’) models. In both cases, we used LASSO regularization to promote sparsity in the regressors, signified by the ‘(sparse)’ suffix in method identifiers, with the regularization hyperparameter *λ* chosen optimally and separately for each model (cf. ‘Hyperparameter selection’ below). In general, we found that *λ* is a moderately sensitive parameter, more so for the whiteness of residuals than for *R*^2^ (see Supplementary Fig. 3 for an example).

#### Linear models with states at the neural level (‘Linear with HRF’, only applicable to fMRI data)

A standard step in the computational modelling of fMRI dynamics is to incorporate a model of the HRF and to separate the underlying neuronal variables from the observed BOLD signals. In this family of models, we thus separated the states **x** from the outputs **y** while keeping a one-to-one relationship between the two (*m* = *n*). We then let the latter be a filtered version of the former through the HRF. For generality and given the natural and important variability of HRF across the brain^74,75^, we allowed the HRF to vary regionally and learned it from the data for all regions in addition to the effective connectivity matrix *W*. Furthermore, for the sake of generality, we allowed both **e**_1_(*t*) and **e**_2_(*t*) to be coloured, with power spectral densities that could also be different between regions and were learned from data. Note that this choice included, as a special case, white **e**_1_(*t*) and **e**_2_(*t*). The result was a highly flexible linear model given by

8a $${{{\bf{x}}}}(t)-{{{\bf{x}}}}(t-1)={{{{W}}}}{{{\bf{x}}}}(t-1)+{{{{\mathcal{G}}}}}_{1}(q){\hat{{{{\bf{e}}}}}}_{1}(t)$$

8b $$\begin{array}{rcl}{{{\bf{y}}}}(t)&=&{{{\mathcal{H}}}}(q){{{\bf{x}}}}(t)+{{{{\mathcal{G}}}}}_{2}(q){\hat{{{{\bf{e}}}}}}_{2}(t)\\ {{{\mathcal{H}}}}(q)&=&\mathop{\sum }\limits_{p=1}^{{n}_{h}}{{{\rm{diag}}}}({{{{{H}}}}}_{:,p}){q}^{-p}\end{array}$$

8c $${{{{\mathcal{F}}}}}_{1}(q)={{{{I}}}}-{{{{\mathcal{G}}}}}_{1}^{-1}(q)=\mathop{\sum }\limits_{p=1}^{{n}_{\phi }}{{{\rm{diag}}}}({{{{{\Phi }}}}}_{:,p}){q}^{-p}$$

8d $${{{{\mathcal{F}}}}}_{2}(q)={{{{I}}}}-{{{{\mathcal{G}}}}}_{2}^{-1}(q)=\mathop{\sum }\limits_{p=1}^{{n}_{\psi }}{{{\rm{diag}}}}({{{{{\Psi }}}}}_{:,p}){q}^{-p}$$

Since LASSO regression produced the best results in our BOLD-level linear models, we used LASSO (with the regularization weight *λ*) to promote sparsity in here. ${{{\mathcal{H}}}}(q)$ is a diagonal matrix whose (*i*, *i*) entry is a linear finite-impulse response (FIR) approximation of the HRF in region *i*, parameterized as in equation (8b) (*q*^−1^ is the standard delay operator, such that *q*^−1^*x*(*t*) = *x*(*t* − 1), see ref. ^25^). Similarly, ${{{{\mathcal{G}}}}}_{1}$ and ${{{{\mathcal{G}}}}}_{2}$ are diagonal filters, parameterized by the inverse FIR forms in equations (8c) and (8d). The matrices ${{{{{H}}}}}_{n\times {n}_{h}}$, ${{{{{\Phi }}}}}_{n\times {n}_{\phi }}$ and ${{{{{\Psi }}}}}_{n\times {n}_{\psi }}$ included learnable FIR parameters of ${{{\mathcal{H}}}}(q)$, ${{{{\mathcal{G}}}}}_{1}^{-1}(q)$ and ${{{{\mathcal{G}}}}}_{2}^{-1}(q)$, respectively. Since the state vector **x**(*t*) was not measured, we learned this model by iterating between state estimation and parameter estimation in an expectation-maximization (EM)-like manner. Note that the presence of filters increases the effective state dimension of the system to $n \max \{{n}_{\phi }+1,{n}_{h}+{n}_{\psi }\}$, considerably increasing the computational complexity of the state estimation step. The final model was taken from the EM iteration with the highest (training) *R*^2^.

#### Linear models with abstract data-driven states (‘Subspace’)

The previous model, despite and because of its extreme generality and flexibility, has a very large state dimension and is extremely difficult to fit. If we forgo the physiological interpretability of the states, then simpler and lower-dimensional models of the form

$$\begin{array}{rcl}{{{\bf{x}}}}(t)-{{{\bf{x}}}}(t-1)&=&{{{{W}}}}{{{\bf{x}}}}(t-1)+{{{{\bf{e}}}}}_{1}(t)\\ {{{\bf{y}}}}(t)&=&{{{{C}}}}{{{\bf{x}}}}(t)+{{{{\bf{e}}}}}_{2}(t)\\ {{{\rm{Cov}}}}\left(\left[\begin{array}{c}{{{{\bf{e}}}}}_{1}(t)\\ {{{{\bf{e}}}}}_{2}(t)\end{array}\right]\right)&=&\left[\begin{array}{cc}{{{{Q}}}}&{{{{M}}}}\\ {{{{{M}}}}}^{T}&{{{{R}}}}\end{array}\right]\end{array}$$

can be learned via subspace identification method^25^. Unlike the model above, states represent abstract low-dimensional regularities within the data, with a dimension *n*_*x*_ that was chosen optimally for each data type. The hyperparameters *r* and *s* represent the amount of output and input–output history used, respectively, in the output Hankel matrix construction and subspace projection steps of the algorithm, respectively (see ref. ^25^ Ch. 10 for details). The noise sequences **e**_1_(*t*) and **e**_2_(*t*) were assumed to be white but could be correlated, and the covariance matrices **Q**, **M** and **R** were also learned from data. Note that this whiteness assumption on noise sequences is without loss of generality in this case due to the subspace method’s ability to learn any non-white dynamics (that is, colour) of noise as part of the abstract state dynamics.

#### Nonlinear NMMs (‘NMM’, ‘NMM with HRF’)

Learning of the models above, except for the ‘Linear with HRF’, involves a convex optimization that can be efficiently solved to find its unique global optimum. In contrast, the learning of nonlinear models is less straightforward. Recently, ref. ^76^ developed an algorithm called MINDy that uses state-of-the-art optimization techniques for learning an NMM of the form

$$\begin{array}{rcl}{{{\bf{x}}}}(t)-{{{\bf{x}}}}(t-1)&=&({{{{W}}}}{\psi }_{{{{\boldsymbol{\alpha }}}}}({{{\bf{x}}}}(t-1))-{{{{D}}}}{{{\bf{x}}}}(t-1)){\Delta }_{T}\\ &&+{{{{\bf{e}}}}}_{1}(t)\\ {{{\bf{y}}}}(t)&=&{{{\mathcal{H}}}}(q){{{\bf{x}}}}(t)+{{{{\bf{e}}}}}_{2}(t)\end{array}$$

using rsfMRI data. In this model, **x**(*t*) has the same dimension as **y**(*t*) (one neural mass per brain region), Δ_*T*_ is the sampling time, *W* is a sparse connectivity matrix, *D* is a diagonal self-decay matrix, *ψ*_***α***_(⋅) is an element-wise sigmoidal nonlinearity whose steepness is determined by each element of the vector ***α*** (which is also the same size as **x**), and ${{{\mathcal{H}}}}(q)$ is a scalar linear HRF that is the same and fixed a priori for all regions. The associated toolbox that we used allows the user to either deconvolve **y**(*t*) using a canonical HRF to obtain the state **x**(*t*) (‘NMM with HRF’), or set ${{{\mathcal{H}}}}(q)=1$ and directly fit the model to **y**(*t*) (‘NMM’). We used both methods for fMRI data but only the latter for iEEG. Since the MINDy algorithm was originally tuned for fMRI, we re-tuned its regularization hyperparameters *λ*_1_,…,*λ*_4_ for use with iEEG data (see ‘Hyperparameter selection’ below).

#### Nonlinear models via MLP DNNs (‘DNN (MLP)’)

Here we used a model of the form in equation (5) for fMRI and trained a ReLU MLP DNN to approximate the function *f*( ⋅ ). The structure of the DNN consists of an input layer, *D* ReLU layers, each preceded by fully connected and batch normalization layers and succeeded by a 50% dropout layer, a final fully connected layer and the output layer. Given the importance of AR lags in the modelling of iEEG, for this modality we generalized equation (5) as

9 $${{{\bf{y}}}}(t)-{{{\bf{y}}}}(t-1)=f({{{\bf{y}}}}(t-1),\ldots ,{{{\bf{y}}}}(t-d))+{{{\bf{e}}}}(t)$$

and similarly approximate *f*(⋅) using an MLP DNN. We used MATLAB’s Deep Learning Toolbox for the training and evaluation of the DNN and tuned the depth *D* and width *W* of the DNN separately for fMRI and iEEG (see ‘Hyperparameter selection’ below).

#### Nonlinear models via convolutional DNNs (‘DNN (CNN)’)

Given the recent success of CNNs in complex learning problems, we also included a model similar to ‘DNN (MLP)’ but with a CNN to approximate the function *f*(⋅). The network consists of an input layer, *D* one-dimensional convolutional layers (convolving over time using *n*_filt_ filters of size *l*_filt_) each succeeded by a batch normalization layer, a ReLU layer and an average pooling layer with a pool size of *n*_pool_, a final dropout layer with probability *p*_drop_, a fully connected layer and the output layer. Spatial convolution was not included in the model, as is the standard in modelling dynamical systems with CNNs, due to the arbitrary nature of channel numbering. Temporal convolution is nevertheless the basis of this model and we thus considered *d* > 1 autoregressive lags for both fMRI and iEEG.

#### Nonlinear models via long short-term memory neural networks (‘LSTM (IIR)’, ‘LSTM (FIR)’)

The above DNN models are inherently static (that is, feedforward), whereas various recurrent neural network architectures have also been proposed for directly modelling dynamical systems. One of the most successful of such architectures are LSTMs which we implemented here in two forms: infinite impulse response (IIR) and finite-impulse response (FIR). These two forms respectively correspond to the two common sequence-to-sequence and sequence-to-one forms of modelling time series using LSTMs. In both cases, the network consists of an input layer, a layer of *W* LSTM units, a fully connected layer and an output layer. The difference is that in the IIR model, the network is initialized once at time 0 and run forward, continuously receiving **y**(*t* − 1) as input and generating **y**(*t*) − **y**(*t* − 1) as output. Each output, therefore, depends on the entire history of the inputs. In the FIR model, on the other hand, the model is initialized and run forward once for each timepoint *t*, receiving only **y**(*t* − *d*),…,**y**(*t* − 1) as input and predicting **y**(*t*) − **y**(*t* − 1) as output.

#### Nonlinear manifold-based models (‘Manifold’)

Consider equations (5) or (9) and assume, for simplicity, that *f* is differentiable. Each of these systems of equations consists of *n* scalar equations, each of which defines a manifold (surface) in *n* + 1 (for equation (5)) or *nd* + 1 (for equation (9)) dimensional space. Various methods have been developed in the machine learning and system identification literature^77,78^ and used in computational neuroscience^20,79^ to capitalize on the fact that in the small vicinity of a point, the manifold can be approximated by a linear hyperplane tangential to it at that point. Here we used the simple method of local polynomial modelling of order 1 (ref. ^77^). To explain this method, first consider the simpler model in equation (5). For each test time *t*^*ℓ*^, we approximate the function *f*(⋅) as a linear function in the vicinity of **y**(*t*^*ℓ*^ − 1), that is,

10 $$f({{{\bf{z}}}})\simeq {{{{\bf{c}}}}}^{\ell }+{{{{{W}}}}}^{\ell }\left[{{{\bf{z}}}}-{{{\bf{y}}}}({t}^{\,\ell }-1)\right]$$

The constant vector **c**^*ℓ*^ and matrix *W*^*ℓ*^ were learned from training data (separately for each test point), as follows. Each training point **y**(*t*^*m*^ − 1) was weighted according to its distance to **y**(*t*^*ℓ*^ − 1), that is,

11 $${k}^{\ell ,m}=\exp \left(-\frac{\parallel {{{\bf{y}}}}({t}^{m}-1)-{{{\bf{y}}}}({t}^{\,\ell }-1){\parallel }^{2}}{2{h}^{2}}\right)$$

where the hyperparameter *h* controls how local or global the model is. These weights were then used in a weighted least squares estimation,

$$\begin{array}{rcl}\left[\begin{array}{cc}{{{{\bf{c}}}}}^{\ell }&{{{{{W}}}}}^{\ell }\end{array}\right]&\simeq &\left(\mathop{\sum}\limits_{{t}^{m}}[{{{\bf{y}}}}({t}^{m})-{{{\bf{y}}}}({t}^{m}-1)]{k}^{\,\ell ,m}{{{{{\boldsymbol{\varphi }}}}}^{\ell ,m}}^{T}\right)\\ &&\cdot {\left(\mathop{\sum}\limits_{{t}^{m}}{{{{\boldsymbol{\varphi }}}}}^{\ell ,m}{k}^{\,\ell ,m}{{{{{\boldsymbol{\varphi }}}}}^{\ell ,m}}^{T}\right)}^{{\dagger} }\end{array}$$

where ^†^ denotes pseudo-inverse and

12 $${{{{\boldsymbol{\varphi }}}}}^{\ell ,m}=\left[\begin{array}{c}1\\ {{{\bf{y}}}}({t}^{m}-1)-{{{\bf{y}}}}({t}^{\ell }-1)\end{array}\right].$$

Note that only the computed **c**^*ℓ*^ was ultimately used, since substituting **z** = **y**(*t*^*ℓ*^ − 1) in equation (10) gives

$$f({{{\bf{y}}}}({t}^{\,\ell }-1))\simeq {{{{\bf{c}}}}}^{\ell }$$

which was used for computing the one-step-ahead prediction at time *t*^*ℓ*^. All the details remain the same when applying this method to equation (9) for iEEG data, except that **y**(*t*^*ℓ*^ − 1) and **y**(*t*^*m*^ − 1) in equations (10), (11) and (12) are replaced with ${[{{{\bf{y}}}}({t}^{\,\ell }-1)^{T}\cdots {{{\bf{y}}}}({t}^{\,\ell }-d)^{T}]^{T}}$ and ${[{{{\bf{y}}}}{({t}^{m}-1)}^{T}\cdots {{{\bf{y}}}}({t}^{m}-d)^{T}]}^{T}$, respectively. We tuned *h* separately for fMRI and iEEG (see ‘Hyperparameter selection’ below), giving rise to values that are so large that they essentially result in a globally linear model (Supplementary Fig. 1). The value of *h* was independently optimized for the computations reported in Fig. 4i,j, as described below.

#### Nonlinear MMSE models (optimal) (‘MMSE (pairwise)’, ‘MMSE (scalar)’)

The models in equation (5) (for fMRI) or equation (9) (for iEEG) ultimately define a stochastic mapping from **y**(*t* − 1) or (**y**(*t* − 1),…,**y**(*t* − *d*)) to **y**(*t*) − **y**(*t* − 1) such that observing the values of the former provides information to predict the latter. It is not hard to show that for two random variables *U* and *V*, the optimal (that is, minimum variance) prediction of *U* given *V* = *v* is given by its conditional expectation $\hat{u}={{{\rm{E}}}}[U| V=v]$ known as the MMSE prediction^80^. Therefore, the optimal prediction of **y**(*t*) − **y**(*t* − 1) given **y**(*t* − 1) or (**y**(*t* − 1),…,**y**(*t* − *d*)) is given by E[**y**(*t*) − **y**(*t* − 1)∣**y**(*t* − 1)] and E[**y**(*t*) − **y**(*t* − 1)∣**y**(*t* − 1),…,**y**(*t* − *d*)], respectively. Due to its optimality, it provides a theoretical upper bound on the achievable accuracy of ‘any’ nonlinear model. The difficulty in calculating this estimate, however, is the estimation of the conditional distribution of **y**(*t*) − **y**(*t* − 1) given an observation of **y**(*t* − 1) or (**y**(*t* − 1),…,**y**(*t* − *d*)). Without imposing additional assumptions (for example, linearity or Gaussianity), this task is not feasible in *n* ≈ 100 dimensions with our limited data points per recording segment. However, this distribution is indeed feasible (1) on a pairwise basis, giving us the optimal predictions E[*y*_*i*_(*t*) − *y*_*i*_(*t* − 1)∣*y*_*j*_(*t* − 1)] for all pairs *i*, *j* = 1,…,*n*, or (2) on a scalar AR basis, yielding the optimal predictions E[*y*_*i*_(*t*) − *y*_*i*_(*t* − 1)∣*y*_*i*_(*t* − 1),…,*y*_*i*_(*t* − *d*)] separately for each *i* = 1,…,*n*. We used the former for fMRI and the latter for iEEG. To estimate this conditional distribution for fMRI, we used a Gaussian window with a standard deviation equal to *β* times the range of *y*_*j*_(*t*) in the training data to detect the training points close to each test *y*_*j*_(*t* − 1) and then used an *N*-point weighted histogram to estimate the (conditional) distribution. More precisely, for any pair of *i* and *j* and any given *test* time *t*^*ℓ*^, we first computed the Gaussian weight

$$\begin{array}{rcl}{w}_{j}^{\ell ,m}&=&\exp \left(-\frac{{[{\,y}_{j}({t}^{m}-1)-{y}_{j}({t}^{\ell }-1)]}^{2}}{2{\sigma }^{2}}\right)\\ \sigma &=&\beta \left(\mathop{\max }\limits_{j,{t}^{m}}{y}_{j}({t}^{m})-\mathop{\min }\limits_{j,{t}^{m}}{y}_{j}({t}^{m})\right)\end{array}$$

for all ‘training’ times *t*^*m*^. Note that *σ* was obtained using only training data. Then, we divided the interval

$$\left[\mathop{\min }\limits_{i,{t}^{m}}{y}_{i}({t}^{m})-{y}_{i}({t}^{m}-1),\,\mathop{\max }\limits_{i,{t}^{m}}{y}_{i}({t}^{m})-{y}_{i}({t}^{m}-1)\right]$$

into *N* equal bins and constructed a weighted histogram of all the training *y*_*i*_(*t*^*m*^) − *y*_*i*_(*t*^*m*^ − 1) where we counted each *y*_*i*_(*t*^*m*^) − *y*_*i*_(*t*^*m*^ − 1) as much as ${w}_{j}^{\ell ,m}$. We then normalized this histogram by dividing all bin values by their sum so that we obtained a well-defined (conditional) probability distribution ${p}_{i,\,j}^{\ell }(\Delta {y}_{i}^{k})$ and estimated the expected value of this probability distribution as

$${{{\rm{E}}}}[{\,y}_{i}(t)-{y}_{i}(t-1)| {\,y}_{j}(t-1)]\simeq \mathop{\sum }\limits_{k=1}^{N}\Delta {y}_{i}^{k} {p}_{i,\,j}^{\ell }(\Delta {y}_{i}^{k})$$

where $\Delta {y}_{i}^{k}$ denotes the centre of the *k*th histogram bin. The case for iEEG is similar, except that the Gaussian weights were computed on the basis of *d*-dimensional Euclidean distances given that we conditioned on *d*-dimensional vectors. In both cases, *β* and *N* are hyperparameters that were tuned separately for fMRI and iEEG (see ‘Hyperparameter selection’ below).

#### Zero model (‘Zero’)

So far, we have discussed several families of models. Comparisons among them will provide a clear picture of which family provides the best fit to the data ‘relative to’ the others. Note however that this process does not necessarily imply that the best model is good in any absolute sense. In other words, all models may be estimating $\hat{{{{\bf{y}}}}}(t| t-1)$ at chance level or lower. Therefore, we also considered the zero model (also known as the zero-order hold, naive model or random walk)

$${{{\bf{y}}}}(t)-{{{\bf{y}}}}(t-1)={{{\bf{e}}}}(t)$$

with the trivial estimate $\hat{{{{\bf{y}}}}}(t| t-1)={{{\bf{y}}}}(t-1)$. Note that this expression corresponds to equation (5) with *f*(**y**(*t* − 1)) = **0** and is only meant to provide a baseline for comparison, not to act as a formal model itself. Also note that this model is different from and often performs better than the constant predictor $\hat{{{{\bf{y}}}}}(t| t-1)=\bar{{{{\bf{y}}}}}$ which constitutes the denominator of *R*^2^.

### Hyperparameter selection

For all models that involve the choice of a design hyperparameter, we simultaneously optimized over all the hyperparameters using SGD with minibatch, separately for fMRI and iEEG. Let *N*_param_ denote the number of hyperparameters in any of the models. Starting from an initial estimate of the hyperparameter vector, in each iteration, ${3}^{{N}_{{{{\rm{param}}}}}}$ hyperparameter vectors were generated, constituting a hypercubic mesh around the current hyperparameter estimate. For integer-valued hyperparameters, we moved 1 point in each direction, while for real-valued hyperparameters, we moved 10^−6^ units. Using a minibatch of randomly selected data segments, the mean-over-minibatch of the median-over-regions of the model *R*^2^ was computed and maximized over the mesh. The random minibatch selection was independent between mesh points and between iterations. For integer-valued hyperparameters, their value was updated to that of the maximizing mesh point. For real-value hyperparameters, a gradient-ascent step was taken in the direction of the largest *R*^2^. The process was repeated until the hyperparameters stopped having a consistent decrease/increase and hovered around a steady-state value (which always happens due to the stochastic nature of SGD) and/or the *R*^2^ stopped having a consistent increase.

For the ‘DNN (CNN)’ and ‘Subspace’ methods (the latter only in iEEG data), the aforementioned procedure was infeasibly slow. This was the case due to a high number of hyperparameters for the CNN model and due to cluster jobs becoming frequently hung and needing to be killed and restarted for the subspace method. As such, we slightly modified the above procedure as follows. In each iteration of the SGD, instead of generating ${3}^{{N}_{{{{\rm{param}}}}}}$ search directions at all the points of a hypercubic mesh, we generated 2*N*_param_ + 1 search directions, one at the current optimum estimate and 2 at the current estimate ±1**e**_*k*_ (for integer-valued) or ±10^−6^**e**_*k*_ (for real-valued) for each *k*th hyperparameter, where **e**_*k*_ is the *k*th canonical unit vector (all zeros except one 1 at the *k*th location). The remaining details were similar to the general case above. Finally, in all cases, we used 100 participants for fMRI (out of the total of 700) and 1,500 segments for iEEG (out of the total of 8,490) for hyperparameter tuning and then removed them from the subsequent model fitting and validation experiments to ensure a lack of overfitting to hyperparameters.

The hyperparameter and *R*^2^ values throughout the process are shown in Supplementary Figs. 7 and 8 for fMRI and iEEG data, respectively, and the final values of the hyperparameters selected for each model are reported in Supplementary Table 1. Note that the initial hyperparameter estimates were chosen on the basis of previous experience, not randomly, which is why they are often very close to or the same as the final values.

### Cross-validation

For the comparisons of HCP data in Fig. 2, we performed the cross-validation as follows, with slightly different procedures for brain-wide and regional methods. For the brain-wide methods, for each of the 700 participants, we split each of the 4 resting scans of that participant into 2 halves, giving a total of 8 segments, each of length 600 samples. All of our methods were then applied using an 8-fold cross-validation where each time, 1 of the 8 segments was used for testing and the remaining 7 were used for training. For pairwise methods, we were forced to lower the sample size due to the extremely high computational complexity of the MMSE predictor. Therefore, instead of each of the above 8 segments (per participant), we used the second quarter of that segment, giving us still an 8-fold cross-validation but on segments of length 150 samples each.

For the comparisons of RAM data in Fig. 3, we first split each of the 283, 5 min recordings into 8,490, 10 s segments. Even though having longer segments would in principle benefit model fitting, 10 s segments ensured that all of our methods could run using the 64 GB available memory per node on the CUBIC cluster. From the 8,490 segments, those that contained any not-a-number (NaN) entries (316 segments) or for which the subspace method produced NaN predictions (30 segments, due to the bad conditioning of the **Φ** matrix therein) were removed from further analysis. Since each recording was already split into 30 segments, and due to the large number of segments, we performed only a single-fold cross-validation on each segment, with the first 8 s used for training and the final 2 s used for cross-validation.

### Multivariate test of whiteness

A standard measure of the goodness of fit in the prediction error method is the whiteness of residuals, measuring the extent to which all temporal structure (that is, dynamics) in the data has been captured by the model. Note that a multivariate time series **e**(*t*) is ‘white’ if it has no statistical dependence across time (that is, **e**(*s*) and **e**(*t*) are independent if *s* ≠ *t*) even though it can have arbitrary statistical dependence across channels (that is, *e*_*i*_(*t*) and *e*_*j*_(*t*) can be dependent at the same time *t*). Parametric (*χ*^2^) statistical tests have been devised for multivariate whiteness, such as the classical Box–Pierce portmanteau test^81^ and its modifications^82,83^. Under strong assumptions, all of these tests have a statistic *Q* (defined slightly differently between them) that is asymptotically (at infinite samples) *χ*^2^ distributed. In our datasets, however, we found that *Q* was not *χ*^2^ distributed, and therefore we used randomization to generate the true null distribution of *Q* by shuffling the time indices of **e**(*t*) 100 times, computing *Q* for each of them and computing the 95th percentile of the randomized *Q* values as the threshold *Q*_thr_ for significance. We used the original definition of *Q* (ref. ^81^),

$$Q=(N-M\,)\mathop{\sum }\limits_{i=1}^{M}{{{\rm{tr}}}}\left({\hat{{{{{R}}}}}}_{{{{\bf{e}}}}}{(i)}^{T}{\hat{{{{{R}}}}}}_{{{{\bf{e}}}}}{(0)}^{-1}{\hat{{{{{R}}}}}}_{{{{\bf{e}}}}}(i){\hat{{{{{R}}}}}}_{{{{\bf{e}}}}}{(0)}^{-1}\right)$$

where *N* is the number of (test) samples, *M* is the number of cross-correlation lags, and

$${\hat{{{{{R}}}}}}_{e}(i)=\frac{1}{N-M}\mathop{\sum }\limits_{t=0}^{N-M-1}{{{\bf{e}}}}(t+i){{{\bf{e}}}}{(t)}^{T},i=0,1,\ldots ,M$$

is a finite-sample estimate of the cross-correlation matrix between channels of **e**(*t*) at lag *i*. Since in practice ${\hat{{{{{R}}}}}}_{{{{\bf{e}}}}}(0)$ may be singular or near-singular, we used the pseudo-inverse of ${\hat{{{{{R}}}}}}_{{{{\bf{e}}}}}(0)$ instead of its inverse in computing *Q*. Finally, only in the case of ‘pairwise’ fMRI models where the residuals are inherently univariate did we use the simpler *χ*^2^ test of whiteness for univariate time series (ref. ^25^ sec. 16.6).

### Nonlinear predictors used for the analysis of the linearizing effects of macroscopic dynamics

Our discussions of linear and nonlinear models and their hyperparameters so far applies to the comparisons shown in Figs. 2 and 3 on neuroimaging time series. In our numerical analysis of the linearizing effects of macroscopic brain dynamics in Fig. 4, we also constructed linear and nonlinear predictors and computed their *R*^2^. The linear predictor was always a simple linear regression model, while the nonlinear predictor was the MMSE predictor for two-dimensional predictions (Fig. 4a–h) and the manifold-based predictor for higher-dimensional predictions (Fig. 4i,j). The MMSE predictor was as described above, except that *β* was adjusted as 0.02 + 0.02/SNR for Fig. 4g,h. For the manifold-based predictor, we used a Gaussian window and swept logarithmically over its hyperparameter *h* from 0.1 to 10 in every iteration and chose the value of *h* that gave the largest *R*^2^. Fig. 4j (left panel) shows the average of the resulting optimal *h* for 100 iterations.

### Simulations involving the Izhikevic model

To show the linearizing effects spatiotemporal averaging on a model whose ground truth is known to be nonlinear, we generated simulated time series from the Izhikevic model^23^

$$\begin{array}{rcl}\dot{v}(t)&=&0.04v{(t)}^{2}+5v(t)+140-u(t)+I\\ \dot{u}(t)&=&a(bv(t)-u(t))\\ (v(t),u(t))&\leftarrow &(c,u(t)+d)\quad \,{{\mbox{if}}}\,\quad v(t)\ge 30\end{array}$$

with *a* = 0.02, *b* = 0.2, *c* = −65 and *d* = 2, *I* = 7. We discretized the model using Euler discretization with 0.1 ms sampling.

### Estimation of rsfMRI SNR

Here we describe our method for the estimation of rsfMRI time series scanner noise and the resulting SNR reported in Supplementary Fig. 4. From the 700 participants used for the study, 50 were selected uniformly at random, and for each selected participant, 1 of their 4 rest scans was selected also uniformly at random. The following was then performed for each of the 50 participant-scans. The rest scan was motion corrected using intramodal linear registration with 6 degrees of freedom (in general, we kept the amount of preprocessing as minimal as possible throughout the SNR estimation algorithm since each preprocessing step often involved averaging and/or interpolation steps that could bias SNR estimates). The first volume of the motion-corrected rest scan was visually inspected and 10 voxels outside of the head were selected. Due to the unavailability of phantom scans, we used these voxels to estimate the scanner noise, while the two (phantom scans and outside voxels) have been shown to yield consistent noise estimates^84^. For each of the 10 voxels, we calculated the temporal variance of the corresponding time series and averaged the results, providing an estimate of scanner noise variance ${\sigma }_{N}^{2}$. To estimate the signal power, a grey matter mask was extracted using each participant’s T1 scan and linearly registered back to the participant’s motion-corrected rest scan. We then computed the temporal variance of each grey matter voxel and averaged the results, yielding an estimate of the combined signal and noise variance. Assuming statistical independence between scanner noise and the participants’ BOLD activity, this combined variance is precisely the sum ${\sigma }_{S}^{2}+{\sigma }_{N}^{2}$ of signal variance and noise variance. The SNR was then calculated as *σ*_*S*_/*σ*_*N*_. Note that this process is inherently conservative and provides an upper bound on the SNR, as it, for instance, does not include any physiological signals into ‘noise’. Therefore, the ratio between the power of signals of neural origin over all other signals contributing to rsfMRI time series may be much lower than 6.5. An SNR of ∼6.5, however, is still low enough to yield a notable linearizing effect, highlighting the importance of measurement noise in downstream computational modelling.

## Citation diversity statement

Recent work in several fields of science has identified a bias in citation practices such that papers from women and other minority scholars are undercited relative to the number of such papers in the field^85–89^. Here we sought to proactively consider choosing references that reflect the diversity of the field in thought, form of contribution, gender, race, ethnicity and other factors. First, we obtained the predicted gender of the first and last author of each reference by using databases that store the probability of a first name being carried by a woman^89,90^. By this measure (and excluding self-citations to the first and last authors of our current paper), our references contained 8.73% woman(first)/woman(last), 18.87% man/woman, 18.34% woman/man and 54.06% man/man. This method is limited in that (1) names, pronouns and social media profiles used to construct the databases may not, in every case, be indicative of gender identity and (2) it cannot account for intersex, non-binary or transgender people. Second, we obtained predicted racial/ethnic category of the first and last author of each reference using databases that store the probability of a first and last name being carried by an author of colour^91,92^. By this measure (and excluding self-citations), our references contained 14.39% author of colour (first)/author of colour(last), 15.47% white author/author of colour, 23.76% author of colour/white author and 46.37% white author/white author. This method is limited in that (1) names and Florida Voter Data used to make the predictions may not be indicative of racial/ethnic identity and (2) it cannot account for Indigenous and mixed-race authors, or those who may face differential biases due to the ambiguous racialization or ethnicization of their names. We look forward to future work that could help us to better understand how to support equitable practices in science.

### Reporting summary

Further information on research design is available in the Nature Portfolio Reporting Summary linked to this article.

### Supplementary information

Supplementary Information Supplementary note, table and figures.

Reporting Summary

## Data Availability

The fMRI and iEEG data supporting the findings of this study are publicly available from the HCP S1200 Release at https://www.humanconnectome.org/study/hcp-young-adult/document/1200-subjects-data-release and the RAM Public Data Release at http://memory.psych.upenn.edu/RAM, respectively.

## Code availability

All the computations of this study (after data preprocessing) were performed in MATLAB, and the corresponding codes are publicly available at https://github.com/enozari/rest-system-id.

## References

[1] Kriegeskorte, N. & Douglas, P. K. Cognitive computational neuroscience. *Nat. Neurosci.* **21**, 1148–1160 (2018). 30127428 10.1038/s41593-018-0210-5 PMC6706072

[2] Wilson, R. C. & Niv, Y. Is model fitting necessary for model-based fMRI? *PLoS Comput. Biol.* **11**, e1004237 (2015). 26086934 10.1371/journal.pcbi.1004237 PMC4472514

[3] Ruff, D. A., Ni, A. M. & Cohen, M. R. Cognition as a window into neuronal population space. *Annu. Rev. Neurosci.* **41**, 77–97 (2018). 29799773 10.1146/annurev-neuro-080317-061936 PMC6571103

[4] Hurwitz, C., Kudryashova, N., Onken, A. & Hennig, M. H. Building population models for large-scale neural recordings: opportunities and pitfalls. *Curr. Opin. Neurobiol.* **70**, 64–73 (2021). 34411907 10.1016/j.conb.2021.07.003

[5] Vyas, S., Golub, M. D., Sussillo, D. & Shenoy, K. V. Computation through neural population dynamics. *Annu. Rev. Neurosci.* **43**, 249 (2020). 32640928 10.1146/annurev-neuro-092619-094115 PMC7402639

[6] Amunts, K. et al. The human brain project: creating a European research infrastructure to decode the human brain. *Neuron* **92**, 574–581 (2016). 27809997 10.1016/j.neuron.2016.10.046

[7] Gu, S. et al. Controllability of structural brain networks. *Nat. Commun.* **6**, 1–10 (2015). 10.1038/ncomms9414 PMC4600713 26423222

[8] Sani, O. G. et al. Mood variations decoded from multi-site intracranial human brain activity. *Nat. Biotechnol.* **36**, 954–961 (2018). 30199076 10.1038/nbt.4200

[9] Izhikevich, E. M. *Dynamical Systems in Neuroscience* (MIT Press, 2007).

[10] Booth, V. & Rinzel, J. A minimal, compartmental model for a dendritic origin of bistability of motoneuron firing patterns. *J Comput. Neurosci.* **2**, 299–312 (1995). 8746404 10.1007/BF00961442

[11] Freeman, W. J. Nonlinear gain mediating cortical stimulus–response relations. *Biol. Cybern.* **33**, 237–247 (1979). 497266 10.1007/BF00337412

[12] Wilson, H. R. & Cowan, J. D. Excitatory and inhibitory interactions in localized populations of model neurons. *Biophys. J.* **12**, 1–24 (1972). 4332108 10.1016/S0006-3495(72)86068-5 PMC1484078

[13] Li, X., Coyle, D., Maguire, L., McGinnity, T. M. & Benali, H. A model selection method for nonlinear system identification based fmri effective connectivity analysis. *IEEE Trans. Med. Imaging* **30**, 1365–1380 (2011). 21335308 10.1109/TMI.2011.2116034

[14] Wang, Y. M., Schultz, R. T., Constable, R. T. & Staib, L. H. Nonlinear Estimation and Modeling of fMRI Data Using Spatio*-*temporal Support Vector Regression. In: Taylor, C., Noble, J.A. (eds) *Information Processing in Medical Imaging. IPMI 2003. Lecture Notes in Computer Science*, vol 2732. 647–659 (Springer, 2003). 10.1007/978-3-540-45087-0_54 15344495

[15] Stephan, K. E. et al. Nonlinear dynamic causal models for fMRI. *Neuroimage* **42**, 649–662 (2008). 18565765 10.1016/j.neuroimage.2008.04.262 PMC2636907

[16] Ritter, P., Schirner, M., McIntosh, A. R. & Jirsa, V. K. The virtual brain integrates computational modeling and multimodal neuroimaging. *Brain Connect*. **3**, 121–145 (2013). 23442172 10.1089/brain.2012.0120 PMC3696923

[17] Stam, C. J. Nonlinear dynamical analysis of EEG and MEG: review of an emerging field. *Clin. Neurophysiol.* **116**, 2266–2301 (2005). 16115797 10.1016/j.clinph.2005.06.011

[18] Ehlers, C. L., Havstad, J., Prichard, D. & Theiler, J. Low doses of ethanol reduce evidence for nonlinear structure in brain activity. *J. Neurosci.* **18**, 7474–7486 (1998). 9736666 10.1523/JNEUROSCI.18-18-07474.1998 PMC6793232

[19] Gultepe, E. & He, B. A linear/nonlinear characterization of resting state brain networks in fMRI time series. *Brain Topogr.* **26**, 39–49 (2013). 22941499 10.1007/s10548-012-0249-7 PMC3537854

[20] Blinowska, K. J. & Malinowski, M. Non-linear and linear forecasting of the EEG time series. *Biol. Cybern.* **66**, 159–165 (1991). 1768720 10.1007/BF00243291

[21] Zhao, Y., Billings, S. A., Wei, H.-L. & Sarrigiannis, P. G. A parametric method to measure time-varying linear and nonlinear causality with applications to EEG data. *IEEE Trans. Biomed. Eng.* **60**, 3141–3148 (2013). 23797214 10.1109/TBME.2013.2269766

[22] Yang, Y., Sani, O. G., Chang, E. F. & Shanechi, M. M. Dynamic network modeling and dimensionality reduction for human ECoG activity. *J. Neural Eng.* **16**, 056014 (2019). 31096206 10.1088/1741-2552/ab2214

[23] Izhikevich, E. M. Simple model of spiking neurons. *IEEE Trans. Neural Netw.* **14**, 1569–1572 (2003). 18244602 10.1109/TNN.2003.820440

[24] Gorrostieta, C., Fiecas, M., Ombao, H., Burke, E. & Cramer, S. Hierarchical vector auto-regressive models and their applications to multi-subject effective connectivity. *Front. Comput. Neurosci.* **7**, 159 (2013). 24282401 10.3389/fncom.2013.00159 PMC3825259

[25] Ljung, L. *System Identification: Theory for the User* (Prentice Hall, 1999).

[26] Kim, S. A. & Ching, S. Quasilinearization-based Controllability Analysis of Neuronal Rate Networks. *2016 American Control Conference (ACC)*, Boston, MA*.* 7371–7376 (IEEE, 2016).

[27] Buzsáki, G., Anastassiou, C. A. & Koch, C. The origin of extracellular fields and currents—EEG, ECoG, LFP and spikes. *Nat. Rev. Neurosci.* **13**, 407–420 (2012). 22595786 10.1038/nrn3241 PMC4907333

[28] Lindén, H., Pettersen, K. H. & Einevoll, G. T. Intrinsic dendritic filtering gives low-pass power spectra of local field potentials. *J. Comput. Neurosci.* **29**, 423–444 (2010). 20502952 10.1007/s10827-010-0245-4

[29] Greve, D. N., Brown, G. G., Mueller, B. A., Glover, G. & Liu, T. T. A survey of the sources of noise in fMRI. *Psychometrika* **78**, 396–416 (2013). 25106392 10.1007/s11336-012-9294-0

[30] Liu, Y., Coon, W., de Pesters, A., Brunner, P. & Schalk, G. The effects of spatial filtering and artifacts on electrocorticographic signals. *J. Neural Eng.* **12**, 056008 (2015). 26268446 10.1088/1741-2560/12/5/056008 PMC5485665

[31] Yang, Z. *Incorporating Structural Bias into Neural Networks for Natural Language Processing*. Ph.D. thesis, Carnegie Mellon Univ. (2019).

[32] Kononova, A. V., Corne, D. W., De Wilde, P., Shneer, V. & Caraffini, F. Structural bias in population-based algorithms. *Inf. Sci.* **298**, 468–490 (2015). 10.1016/j.ins.2014.11.035

[33] Mehta, R., Chung, J. Shen, C., Xu, T. & Vogelstein, J. T. A consistent independence test for multivariate time-series. Preprint at 10.48550/arXiv.1908.06486 (2019).

[34] Dafilis, M. P., Sinclair, N. C., Cadusch, P. J. & Liley, D. T. Re-evaluating the performance of the nonlinear prediction error for the detection of deterministic dynamics. *Physica D* **240**, 695–700 (2011). 10.1016/j.physd.2010.12.001

[35] Deneux, T. & Faugeras, O. Using nonlinear models in fMRI data analysis: model selection and activation detection. *Neuroimage* **32**, 1669–1689 (2006). 16844388 10.1016/j.neuroimage.2006.03.006

[36] Liu, Z. et al. Linear and nonlinear relationships between visual stimuli, EEG and bold fMRI signals. *Neuroimage* **50**, 1054–1066 (2010). 20079854 10.1016/j.neuroimage.2010.01.017 PMC2841568

[37] Schulz, M.-A. et al. Different scaling of linear models and deep learning in UKBiobank brain images versus machine-learning datasets. *Nat. Commun.* **11**, 1–15 (2020). 32843633 10.1038/s41467-020-18037-z PMC7447816

[38] He, T. et al. Deep neural networks and kernel regression achieve comparable accuracies for functional connectivity prediction of behavior and demographics. *NeuroImage* **206**, 116276 (2020). 31610298 10.1016/j.neuroimage.2019.116276 PMC6984975

[39] Wobst, P., Wenzel, R., Kohl, M., Obrig, H. & Villringer, A. Linear aspects of changes in deoxygenated hemoglobin concentration and cytochrome oxidase oxidation during brain activation. *Neuroimage* **13**, 520–530 (2001). 11170817 10.1006/nimg.2000.0706

[40] Rumyantsev, O. I. et al. Fundamental bounds on the fidelity of sensory cortical coding. *Nature* **580**, 100–105 (2020). 32238928 10.1038/s41586-020-2130-2

[41] Khalil, H. K. *Nonlinear Systems* (Prentice Hall, 2002).https://books.google.com/books?id=t_d1QgAACAAJ

[42] Palva, J. M. & Palva, S. Functional integration across oscillation frequencies by cross-frequency phase synchronization. *Eur. J. Neurosci.* **48**, 2399–2406 (2018). 29094462 10.1111/ejn.13767

[43] Zañudo, J. G. T., Yang, G. & Albert, R. Structure-based control of complex networks with nonlinear dynamics. *Proc. Natl Acad. Sci. USA* **114**, 7234–7239 (2017). 28655847 10.1073/pnas.1617387114 PMC5514702

[44] Rozum, J. C. & Albert, R. Identifying (un)controllable dynamical behavior in complex networks. *PLoS Comput. Biol.* **14**, e1006630 (2018). 30532150 10.1371/journal.pcbi.1006630 PMC6301693

[45] Tang, E. & Bassett, D. S. Colloquium: control of dynamics in brain networks. *Rev. Mod. Phys.* **90**, 031003 (2018). 10.1103/RevModPhys.90.031003

[46] Towlson, E. K. et al. *Caenorhabditis elegans* and the network control framework–FAQs. *Phil. Trans. R. Soc. Lond. B* **373**, 20170372 (2018). 30201837 10.1098/rstb.2017.0372 PMC6158218

[47] Karrer, T. M. et al. A practical guide to methodological considerations in the controllability of structural brain networks. *J. Neural Eng.* **17**, 026031 (2020). 31968320 10.1088/1741-2552/ab6e8b PMC7734595

[48] Goutte, C., Nielsen, F. A. & Hansen, L. K. Modeling the hemodynamic response in fMRI using smooth FIR filters. *IEEE Trans. Med. Imaging* **19**, 1188–1201 (2000). 11212367 10.1109/42.897811

[49] Donoghue, T. et al. Parameterizing neural power spectra into periodic and aperiodic components. *Nat. Neurosci.* **23**, 1655–1665 (2020). 10.1038/s41593-020-00744-x PMC8106550 33230329

[50] Bansal, K., Nakuci, J. & Muldoon, S. F. Personalized brain network models for assessing structure–function relationships. *Curr. Opin. Neurobiol.* **52**, 42–47 (2018). 29704749 10.1016/j.conb.2018.04.014

[51] Schirner, M., Rothmeier, S., Jirsa, V. K., McIntosh, A. R. & Ritter, P. An automated pipeline for constructing personalized virtual brains from multimodal neuroimaging data. *Neuroimage* **117**, 343–357 (2015). 25837600 10.1016/j.neuroimage.2015.03.055

[52] Bayrak, S., Hövel, P. & Vuksanović, V. in *Modeling Functional Connectivity on Empirical and Randomized Structural Brain Networks* (Springer, 2017).

[53] Saenger, V. M. et al. Uncovering the underlying mechanisms and whole-brain dynamics of deep brain stimulation for Parkinson’s disease. *Sci. Rep.* **7**, 1–14 (2017). 28851996 10.1038/s41598-017-10003-y PMC5574998

[54] Zarghami, T. S. & Friston, K. J. Dynamic effective connectivity. *Neuroimage* **207**, 116453 (2020). 31821868 10.1016/j.neuroimage.2019.116453

[55] Friston, K. J. et al. DCM for complex-valued data: cross-spectra, coherence and phase-delays. *Neuroimage* **59**, 439–455 (2012). 21820062 10.1016/j.neuroimage.2011.07.048 PMC3200431

[56] Becker, C. O., Bassett, D. S. & Preciado, V. M. Large-scale dynamic modeling of task-fMRI signals via subspace system identification. *J. Neural Eng.* **15**, 066016 (2018). 30088476 10.1088/1741-2552/aad8c7

[57] Yang, Y., Connolly, A. T. & Shanechi, M. M. A control-theoretic system identification framework and a real-time closed-loop clinical simulation testbed for electrical brain stimulation. *J. Neural Eng.* **15**, 066007 (2018). 30221624 10.1088/1741-2552/aad1a8

[58] Barch, D. M. Resting-state functional connectivity in the human connectome project: current status and relevance to understanding psychopathology. *Harv. Rev. Psychiatry* **25**, 209–217 (2017). 28816791 10.1097/HRP.0000000000000166 PMC5644502

[59] Burgess, G. C. et al. Evaluation of denoising strategies to address motion-correlated artifacts in resting-state functional magnetic resonance imaging data from the human connectome project. *Brain Connect*. **6**, 669–680 (2016). 27571276 10.1089/brain.2016.0435 PMC5105353

[60] Elam, J. HCP data release updates: known issues and planned fixes. https://wiki.humanconnectome.org/display/PublicData/HCP+Data+Release+Updates%3A+Known+Issues+and+Planned+fixes (2020).

[61] Schaefer, A. et al. Local–global parcellation of the human cerebral cortex from intrinsic functional connectivity MRI. *Cereb. Cortex* **28**, 3095–3114 (2018). 28981612 10.1093/cercor/bhx179 PMC6095216

[62] Tian, Y., Margulies, D.S., Breakspear, M. & Zalesky, A. Topographic organization of the human subcortex unveiled with functional connectivity gradients. *Nat. Neurosci.* **23**, 1421–1432 (2020). 32989295 10.1038/s41593-020-00711-6

[63] Stiso, J. et al. White matter network architecture guides direct electrical stimulation through optimal state transitions. *Cell Rep.* **28**, 2554–2566.e7 (2019). 31484068 10.1016/j.celrep.2019.08.008 PMC6849479

[64] Khambhati, A. N. et al. Functional control of electrophysiological network architecture using direct neurostimulation in humans. *Netw. Neurosci.* **3**, 848–877 (2019). 31410383 10.1162/netn_a_00089 PMC6663306

[65] Betzel, R. F. et al. Structural, geometric and genetic factors predict interregional brain connectivity patterns probed by electrocorticography. *Nat. Biomed. Eng.* **3**, 902–916 (2019). 31133741 10.1038/s41551-019-0404-5

[66] Ung, H. et al. Interictal epileptiform activity outside the seizure onset zone impacts cognition. *Brain* **140**, 2157–2168 (2017). 28666338 10.1093/brain/awx143 PMC6167607

[67] Ren, S., Gliske, S. V., Brang, D. & Stacey, W. C. Redaction of false high frequency oscillations due to muscle artifact improves specificity to epileptic tissue. *Clin. Neurophysiol.* **130**, 976–985 (2019). 31003116 10.1016/j.clinph.2019.03.028 PMC6551620

[68] Owen, L. L. et al. A Gaussian process model of human electrocorticographic data. *Cereb. Cortex* **30**, 5333–5345 (2020). 32495832 10.1093/cercor/bhaa115 PMC7472198

[69] Prime, D., Rowlands, D., O’Keefe, S. & Dionisio, S. Considerations in performing and analyzing the responses of cortico-cortical evoked potentials in stereo-EEG. *Epilepsia* **59**, 16–26 (2018). 29143307 10.1111/epi.13939

[70] Delorme, A., Sejnowski, T. & Makeig, S. Enhanced detection of artifacts in EEG data using higher-order statistics and independent component analysis. *Neuroimage* **34**, 1443–1449 (2007). 17188898 10.1016/j.neuroimage.2006.11.004 PMC2895624

[71] Stiso, J. et al. Fluctuations in functional connectivity associated with interictal epileptiform discharges (IEDS) in intracranial EEG. Preprint at *bioRxiv*10.1101/2021.05.14.444176 (2022).

[72] Lawhern, V., Hairston, W. D., McDowell, K., Westerfield, M. & Robbins, K. Detection and classification of subject-generated artifacts in EEG signals using autoregressive models. *J. Neurosci. Methods* **208**, 181–189 (2012). 22634706 10.1016/j.jneumeth.2012.05.017

[73] Bressler, S. L., Richter, C. G., Chen, Y. & Ding, M. Cortical functional network organization from autoregressive modeling of local field potential oscillations. *Stat. Med.* **26**, 3875–3885 (2007). 17551946 10.1002/sim.2935

[74] Deshpande, R., Wu, G.-R., Marinazzo, D., Hu, X. & Deshpande, G. Hemodynamic response function (HRF) variability confounds resting-state fMRI functional connectivity. *Magn. Reson. Med.* **80**, 1697–1713 (2018). 29656446 10.1002/mrm.27146

[75] Taylor, A. J., Kim, J. H. & Ress, D. Characterization of the hemodynamic response function across the majority of human cerebral cortex. *Neuroimage* **173**, 322–331 (2018). 29501554 10.1016/j.neuroimage.2018.02.061 PMC5911213

[76] Singh, M., Braver, T., Cole, M. & Ching, S. Estimation and validation of individualized dynamic brain models with resting state fMRI. *Neuroimage* **221**, 117046 (2019). 10.1016/j.neuroimage.2020.117046 PMC7875185 32603858

[77] Roll, J. *Local and Piecewise Affine Approaches to System Identification*. Ph.D. thesis, Linkoping Univ. (2003).

[78] Ljung, L. Approaches to Identification of Nonlinear Systems. *Proceedings of the 29th Chinese Control Conference*, Beijing, China. 1–5 (IEEE, 2010).

[79] Popivanov, D., Dushanova, J., Mineva, A. & Krekule, I. Detection of Successive Changes in Dynamics of EEG Time Series: Linear and Nonlinear Approach. *Proceedings of 18th Annual International Conference of the IEEE Engineering in Medicine and Biology Society*, Amsterdam, Netherlands. Vol. 4 1590–1591 (IEEE, 1996).

[80] Poor, H. V. *An introduction to signal detection and estimation* (Springer, 2013).

[81] Box, G. E. & Pierce, D. A. Distribution of residual autocorrelations in autoregressive-integrated moving average time series models. *J. Am. Stat. Assoc.* **65**, 1509–1526 (1970). 10.1080/01621459.1970.10481180

[82] Ljung, G. M. & Box, G. E. P. On a measure of lack of fit in time series models. *Biometrika* **65**, 297–303 (1978). 10.1093/biomet/65.2.297

[83] Li, W. K. & McLeod, A. I. Distribution of the residual autocorrelations in multivariate ARMA time series models. *J. R. Stat. Soc. B* **43**, 231–239 (1981). 10.1111/j.2517-6161.1981.tb01175.x

[84] Chen, C.-C. & Tyler, C. W. Spectral Analysis of fMRI Signal and Noise. In: Onozuka, M., Yen, CT. (eds) *Novel Trends in Brain Science*. 63–76 (Springer, 2008).

[85] Mitchell, S. M., Lange, S. & Brus, H. Gendered citation patterns in international relations journals. *Int. Stud. Perspect.* **14**, 485–492 (2013). 10.1111/insp.12026

[86] Dion, M. L., Sumner, J. L. & Mitchell, S. M. Gendered citation patterns across political science and social science methodology fields. *Polit. Anal.* **26**, 312–327 (2018). 10.1017/pan.2018.12

[87] Caplar, N., Tacchella, S. & Birrer, S. Quantitative evaluation of gender bias in astronomical publications from citation counts. *Nat. Astron.* **1**, 0141 (2017). 10.1038/s41550-017-0141

[88] Maliniak, D., Powers, R. & Walter, B. F. The gender citation gap in international relations. *Int. Organ.* **67**, 889–922 (2013). 10.1017/S0020818313000209

[89] Dworkin, J. D. et al. The extent and drivers of gender imbalance in neuroscience reference lists. *Nat. Neurosci.* **23**, 918–926 (2020). 10.1038/s41593-020-0658-y 32561883

[90] Zhou, D. et al. Gender diversity statement and code notebook v1.0. *Zenodo*10.5281/zenodo.3672110 (2020).

[91] Ambekar, A., Ward, C., Mohammed, J., Male, S. & Skiena, S. Name-ethnicity classification from open sources. In in *Proc. 15th ACM SIGKDD International Conference on Knowledge Discovery and Data Mining* 49–58 (ACM, 2009).

[92] Laohaprapanon, S. & Sood, G. Predicting race and ethnicity from the sequence of characters in a name. Preprint at https://arxiv.org/abs/1805.02109v1 (2018).

