# Supplementary material for: Macroscopic resting-state brain dynamics are best described by linear models
Source: Nat Biomed Eng. 2023 Dec 11;8(1):68–84. doi: 10.1038/s41551-023-01117-y (PMC11357987; doi:10.1038/s41551-023-01117-y)
Supplement: Supplementary file 1 — Supplementary note, table and figures. [file 41551_2023_1117_MOESM1_ESM.pdf]

nature biomedical engineering

Article

https://doi.org/10.1038/s41551-023-01117-y

Macroscopic resting-state brain dynamics are best described by linear models

In the format provided by the authors and unedited

Contents

1 Supplementary Note 1: Effect of Bandpass Filtering on System Identification and the Detection of Nonlinearity 2

2 Supplementary Table 1: List of linear and nonlinear families of models 4

3 Supplementary Figures 5

List of Supplementary Figures

1 Effect of window size h on the accuracy of the manifold-based locally linear ('Manifold') model in fMRI data. 5
2 The effects of the number of lags and sparsity patterns on the prediction accuracy and computational complexity of linear AR models of rsfMRI. 6
3 The effect of the LASSO parameter λ on the accuracy of the 'VAR-3 (sparse)' model. 7
4 Histogram of scanner SNR estimates for rsfMRI data. 8
5 Separate training and test times for system identification methods of rsfMRI. 9
6 Separate training and test times for system identification methods of rsiEEG. 10
7 Hyper-parameter tuning of linear and nonlinear model families for fMRI. 12
8 Hyper-parameter tuning of linear and nonlinear model families for iEEG. 14
9 Comparing the 'DNN (MLP)' model with linear models in fitting simulated data from the Izhikevic model. 15
10 Comparison of 'Liner (dense)' and 'DNN (MLP)' models on the logistic map dynamical system. 16
11 Comparing 'DNN (MLP)' model with a linear activation function against 'Linear (dense)' and 'Linear (sparse)'. 17
12 Linear vs. nonlinear models of finely-parcellated rsfMRI activity. 18
13 Linear vs. nonlinear models of unparcellated cortical rsfMRI activity. 20
14 Linear vs. nonlinear models of unparcellated subcortical rsfMRI activity. 21
15 Linear vs. nonlinear models of minimally pre-processed rsfMRI activity. 22
16 Test-retest validation of model comparisons for rsfMRI data. 23
17 The channel-wise R^2 distribution of the zero model for iEEG data with different subsampling ratios and the corresponding sampling frequency. 24
18 Linear versus nonlinear models of 5-fold subsampled rsiEEG activity. 25
19 Linear versus nonlinear models of 25-fold subsampled rsiEEG activity. 26
20 k-step ahead prediction of rsfMRI data. 27
21 k-step ahead prediction of rsiEEG data. 28
22 Model comparisons on iEEG data without channel removal. 29

1 Supplementary Note 1: Effect of Bandpass Filtering on System Identification and the Detection of Nonlinearity

A standard step in the preprocessing of resting state fMRI time series is bandpass filtering, typically over the range $[0.01, 0.08]$ Hz [1], to reduce the contribution of non-neuronal sources on the signal and improve the SNR. In this work, however, we purposefully avoided this step. In the following, we discuss in detail the role and effects of pre-filtering in this rather unconventional context of system identification and, in particular, detection of nonlinear dynamics.

1. First, band-pass filtering (or any linear filtering for this matter) has no effect on the fitting or evaluation of linear models. The reason, in short, is the commuting property of linear systems. More specifically, any linear system including all of the ones used in this work can be written in the impulse-response form [2]

$$\mathbf{y}(t) = \mathbf{G}(q)\mathbf{e}(t),$$

where $\mathbf{G}$ is the transfer matrix

$$\mathbf{G}(q) = \begin{bmatrix} G_{11}(q) & G_{12}(q) & \cdots \\ G_{21}(q) & G_{22}(q) & \cdots \\ \vdots & \vdots & \ddots \end{bmatrix}$$

such that for any i and j

$$G_{ij}(q) = g_{ij}(0) + g_{ij}(1)q^{-1} + g_{ij}(2)q^{-2} + \cdots$$

Here, $g_{ij}(t)$ is the impulse response from the the jth input $e_j(t)$ to the ith output $y_i(t)$, and q is the standard shift operator such that $q^{-1}s(t) = s(t-1)$ for any signal $s(t)$ [3]. Recall that $\mathbf{y}(t)$ is the BOLD time series without band-pass filtering, as used in the main text, and let

$$F(q) = f(0) + f(1)q^{-1} + f(2)q^{-2} + \cdots$$

be any linear filter, including the bandpass filter used in common preprocessing pipelines. Assume, without loss of generality, that $f(0) \neq 0$ (an almost identical argument can be given if $f(0)$ or any number of the first terms in $F(q)$ are zero, simply by factoring out enough powers of q^{-1}). The output of the filter is

$$\mathbf{z}(t) = F(q)\mathbf{y}(t) = F(q)\mathbf{G}(q)\mathbf{e}(t).$$

It then immediately follows from the prediction error framework [3] that the one step ahead prediction error of $\mathbf{z}(t)$ is given by

$$\begin{aligned} \mathbf{z}(t) - \hat{\mathbf{z}}(t|t-1) &= f(0)\mathbf{G}^{-1}(q)F^{-1}(q)\mathbf{z}(t) \\ &= f(0)\mathbf{G}^{-1}(q)\mathbf{y}(t) \\ &= f(0)\big[\mathbf{y}(t) - \hat{\mathbf{y}}(t|t-1)\big]. \end{aligned}$$

In other words, the prediction error of $\mathbf{z}(t)$ is identical to the prediction error of $\mathbf{y}(t)$, except for a constant factor equal to the instantaneous gain of the filter. Therefore, not only is fitting a model by minimizing the prediction error of $\mathbf{z}(t)$ identical to fitting a model by minimizing the prediction error of $\mathbf{y}(t)$, but the cross-validated R^2 (up to a fixed constant) and residual whiteness of these models are also identical.

This argument clearly fails for nonlinear systems. Indeed, fitting a nonlinear model on $\mathbf{z}(t)$ can result in a different model with different R^2 and residual whiteness than the same model fit on $\mathbf{y}(t)$. The critical point, however, is that the linear filter $F(q)$ cannot generate nonlinearity, while it can certainly weaken and even eliminate it. We explain these two points in more detail next.

2. Assume, first, that the dynamics of $\mathbf{y}(t)$ are truly linear, as seems to be the case from our analysis in the main text. Then, the relationship between $\Delta\mathbf{y}(t)$ and $\mathbf{y}(t-1)$ (as random vectors) is linear. By definition, if the relationship between two random vectors $\mathbf{u}$ and $\mathbf{v}$ is linear, they can be written in the form

$$\begin{bmatrix}\mathbf{u}\\ \mathbf{v}\end{bmatrix} = \begin{bmatrix}\mathbf{A}_{11} & \mathbf{A}_{12}\\ \mathbf{A}_{21} & \mathbf{A}_{22}\end{bmatrix}\begin{bmatrix}\mathbf{e}_1\\ \mathbf{e}_2\end{bmatrix} \tag{S1}$$

where $\mathbf{A}$ is an appropriate matrix and $\mathbf{e}_1$ and $\mathbf{e}_2$ are independent. Now let $\mathbf{u}_F$ be the result of applying a linear filter $F(q)$ to (the samples of) $\mathbf{u}$. In other words, if N is the order of an (arbitrarily accurate) FIR approximation of $F(q)$,

$$\mathbf{u}_F = \begin{bmatrix}\mathbf{u}_1 & \mathbf{u}_2 & \cdots & \mathbf{u}_N\end{bmatrix}\begin{bmatrix}f(0)\\ f(1)\\ \vdots\\ f(N-1)\end{bmatrix} = \begin{bmatrix}\mathbf{u}_1 & \mathbf{u}_2 & \cdots & \mathbf{u}_N\end{bmatrix}\mathbf{f} \tag{S2}$$

where $f(t)$ is the impulse response of $F(q)$ and $\mathbf{u}_1, \mathbf{u}_2, \ldots, \mathbf{u}_N$ are identically distributed (but not necessarily independent) samples of $\mathbf{u}$. From Eq. (S1),

$$\begin{bmatrix}\mathbf{u}_1 & \mathbf{u}_2 & \cdots & \mathbf{u}_N\end{bmatrix} = \begin{bmatrix}\mathbf{A}_{11} & \mathbf{A}_{12}\end{bmatrix}\begin{bmatrix}\mathbf{e}_{1,1} & \mathbf{e}_{1,2} & \cdots & \mathbf{e}_{1,N}\\ \mathbf{e}_{2,1} & \mathbf{e}_{2,2} & \cdots & \mathbf{e}_{2,N}\end{bmatrix} \tag{S3}$$

where $\mathbf{e}_{1,1}, \ldots, \mathbf{e}_{1,N}$ are identically distributed (but not necessarily independent) samples of $\mathbf{e}_1$, similarly for $\mathbf{e}_2$. Note that each $\mathbf{e}_{1,t}$ is still independent from each $\mathbf{e}_{2,s}$ by definition. Substituting Eq. (S3) into Eq. (S2) thus gives

$$\begin{aligned}\mathbf{u}_F &= \begin{bmatrix}\mathbf{A}_{11} & \mathbf{A}_{12}\end{bmatrix}\begin{bmatrix}\mathbf{e}_{1,1} & \mathbf{e}_{1,2} & \cdots & \mathbf{e}_{1,N}\\ \mathbf{e}_{2,1} & \mathbf{e}_{2,2} & \cdots & \mathbf{e}_{2,N}\end{bmatrix}\mathbf{f}\\ &= \begin{bmatrix}\mathbf{A}_{11} & \mathbf{A}_{12}\end{bmatrix}\begin{bmatrix}\mathbf{e}_{1,F}\\ \mathbf{e}_{2,F}\end{bmatrix}\end{aligned}$$

where $\mathbf{e}_{1,F}$ and $\mathbf{e}_{2,F}$ are filtered versions of $\mathbf{e}_1$ and $\mathbf{e}_2$ and, still, independent of each other. Following the same steps for $\mathbf{v}$, we get

$$\begin{bmatrix}\mathbf{u}_F\\ \mathbf{v}_F\end{bmatrix} = \begin{bmatrix}\mathbf{A}_{11} & \mathbf{A}_{12}\\ \mathbf{A}_{21} & \mathbf{A}_{22}\end{bmatrix}\begin{bmatrix}\mathbf{e}_{1,F}\\ \mathbf{e}_{2,F}\end{bmatrix}$$

showing that $\mathbf{u}_F$ and $\mathbf{v}_F$ are still linearly related after filtering. This is indeed expected since a linear filter cannot generate nonlinear dependence between two signals that are originally linearly related.

3. Now assume, in contrast, that the dynamics of $\mathbf{y}(t)$ is in fact nonlinear and we filter $\mathbf{y}(t)$ to get $\mathbf{z}(t) = F(q)\mathbf{y}(t)$. The best that can happen, as far as detecting nonlinearity is concerned, is that the dynamics of $\mathbf{z}(t)$ remain nonlinear. However, it is possible that $F(q)$ weakens or completely averages out the nonlinearities in $\mathbf{y}(t)$, as we saw in the main text. In fact, the common bandpass filter over $[0.01, 0.08]$ Hz (compared to a Nyquist frequency of $1/2\text{TR} \simeq 0.7$ Hz) is strongly lowpass and involves significant averaging over time.

In conclusion, while bandpass filtering has no effect on linear models and preserves linearity of time series, it can well weaken/eliminate any nonlinearity in the time series. Therefore, regardless of how much "cleaner" bandpass-filtered data might be, finding no nonlinearity before bandpass filtering, as pursued in the main text, is a stronger statement than the same finding would be if obtained after bandpass filtering.

2 Supplementary Table 1: List of linear and nonlinear families of models

Supplementary Table 1 | List of linear and nonlinear families of models. The marks † and ‡ indicate, respectively, that a method is used only for fMRI or iEEG. See Methods for a description of each model.

Label	Title	Equation	Hyper-parameters
Linear (dense)	Linear models with states at the BOLD/LFP level	$\mathbf{y}(t) - \mathbf{y}(t-1) = \mathbf{W}\mathbf{y}(t-1) + \mathbf{e}(t)$	None
Linear (sparse)			$\lambda = 0.95$ (fMRI)<br>$\lambda = 1.35$ (iEEG)
Linear (pairwise)†		$y_i(t) - y_i(t-1) = w_{ij}y_j(t-1) + e_i(t), i, j = 1, \dots, n$	None
AR-2 (sparse)†	Linear autoregressive models	$\mathbf{y}(t) - \mathbf{y}(t-1) = \mathbf{W}\mathbf{y}(t-1) + \mathbf{D}_2\mathbf{y}(t-2) + \mathbf{D}_3\mathbf{y}(t-3) + \cdots + \mathbf{D}_d\mathbf{y}(t-d) + \mathbf{e}(t)$	$d = 2, \lambda = 0.95$, diagonal $\mathbf{D}_2$
VAR-2 (sparse)†			$d = 2, \lambda = 0.9$
AR-3 (sparse)†			$d{=}3, \lambda{=}0.5$, diagonal $\mathbf{D}_2, \mathbf{D}_3$
VAR-3 (sparse)†			$d = 3, \lambda = 0.35$
AR-100 (sparse)‡			$d = 112, \lambda = 1.35$
AR-100 (scalar)‡			$d = 112$
Linear w/ HRF†	Linear models with states at the neural level	$\mathbf{x}(t) - \mathbf{x}(t-1) = \mathbf{W}\mathbf{x}(t-1) + \mathcal{G}_1(q)\hat{\mathbf{e}}_1(t)$<br>$\mathbf{y}(t) = \mathcal{H}(q)\mathbf{x}(t) + \mathcal{G}_2(q)\hat{\mathbf{e}}_2(t)$<br>$\mathcal{H}(q) = \sum_{p=1}^{n_h} \mathrm{diag}(\mathbf{H}_{:,p})q^{-p}$<br>$\mathcal{F}_1(q) = \mathbf{I} - \mathcal{G}_1^{-1}(q) = \sum_{p=1}^{n_\phi} \mathrm{diag}(\mathbf{\Phi}_{:,p})q^{-p}$<br>$\mathcal{F}_2(q) = \mathbf{I} - \mathcal{G}_2^{-1}(q) = \sum_{p=1}^{n_\psi} \mathrm{diag}(\mathbf{\Psi}_{:,p})q^{-p}$	$n_h = n_\phi = n_\psi = 5, \lambda = 11$
Subspace	Linear models with abstract data-driven states	$\mathbf{x}(t) - \mathbf{x}(t-1) = \mathbf{W}\mathbf{x}(t-1) + \mathbf{e}_1(t)$<br>$\mathbf{y}(t) = \mathbf{C}\mathbf{x}(t) + \mathbf{e}_2(t)$<br>$\mathrm{Cov}\left(\begin{bmatrix}\mathbf{e}_1(t)\\ \mathbf{e}_2(t)\end{bmatrix}\right) = \begin{bmatrix}\mathbf{Q} & \mathbf{M}\\ \mathbf{M}^T & \mathbf{R}\end{bmatrix}$	$s = 1, r = 3, n_x = 25$ (fMRI)<br>$s = 10, r = 69, n_x = 445$ (iEEG)
NMM	Nonlinear neural mass models	$\mathbf{y}(t) - \mathbf{y}(t-1) = (\mathbf{W}\psi_{\boldsymbol{\alpha}}(\mathbf{y}(t-1)) - \mathbf{D}\mathbf{y}(t-1))\Delta_T + \mathbf{e}(t)$	MINDy default (fMRI)<br>$\lambda_1 = \lambda_2 = 0.2, \lambda_3 = 0.7$,<br>$\lambda_4 = 0.5$ (iEEG)
NMM w/ HRF†		$\mathbf{x}(t) - \mathbf{x}(t-1) = (\mathbf{W}\psi_{\boldsymbol{\alpha}}(\mathbf{x}(t-1)) - \mathbf{D}\mathbf{x}(t-1))\Delta_T + \mathbf{e}_1(t)$<br>$\mathbf{y}(t) = \mathcal{H}(q)\mathbf{x}(t) + \mathbf{e}_2(t)$	MINDy default
DNN (MLP)	Nonlinear models via multi-layer perceptron deep neural networks	$\mathbf{y}(t) - \mathbf{y}(t-1) = f(\mathbf{y}(t-1), \dots, \mathbf{y}(t-d)) + \mathbf{e}(t)$	$d = 1, D = 6, W = 2$ (fMRI)<br>$d = 4, D = 3, W = 29$ (iEEG)
DNN (CNN)	Nonlinear models via convolutional deep neural networks	$\mathbf{y}(t) - \mathbf{y}(t-1) = f(\mathbf{y}(t-1), \dots, \mathbf{y}(t-d)) + \mathbf{e}(t)$	$d = 17, D = 2, l_{\mathrm{filt}} = 7$,<br>$n_{\mathrm{filt}} = 11, n_{\mathrm{pool}} = 4$,<br>$p_{\mathrm{drop}} = 0.4$ (fMRI)<br>$d = 13, D = 2, l_{\mathrm{filt}} = 5$,<br>$n_{\mathrm{filt}} = 10, n_{\mathrm{pool}} = 2$,<br>$p_{\mathrm{drop}} = 0.51$ (iEEG)
LSTM (IIR)	Nonlinear models via long short-term memory recurrent neural networks	$\mathbf{y}(t) - \mathbf{y}(t-1) = f(\mathbf{y}(t-1), \dots, \mathbf{y}(0)) + \mathbf{e}(t)$	$W = 12$ (fMRI)<br>$W = 7$ (iEEG)
LSTM (FIR)		$\mathbf{y}(t) - \mathbf{y}(t-1) = f(\mathbf{y}(t-1), \dots, \mathbf{y}(t-d)) + \mathbf{e}(t)$	$d = 1, W = 16$ (fMRI)<br>$d = 32, W = 2$ (iEEG)
Manifold	Nonlinear manifold-based models	$\mathbf{y}(t) - \mathbf{y}(t-1) = f(\mathbf{y}(t-1), \dots, \mathbf{y}(t-d)) + \mathbf{e}(t)$	$d = 1, h = 830$ (fMRI)<br>$d = 7, h = 1.3 \times 10^4$ (iEEG)
MMSE (pairwise)†	Nonlinear minimum mean squared error models (optimal)	$y_i(t) - y_i(t-1) = \mathrm{E}[y_i(t) - y_i(t-1) \mid y_j(t-1)], i, j = 1, \dots, n$	$N = 280, \beta = 0.156$
MMSE (scalar)‡		$y_i(t) - y_i(t-1) = \mathrm{E}[y_i(t) - y_i(t-1) \mid y_i(t-1), \dots, y_i(t-d)], i = 1, \dots, n$	$d = 9, N = 309, \beta = 0.007$
Zero	Zero model	$\mathbf{y}(t) - \mathbf{y}(t-1) = \mathbf{e}(t)$	None

3 Supplementary Figures

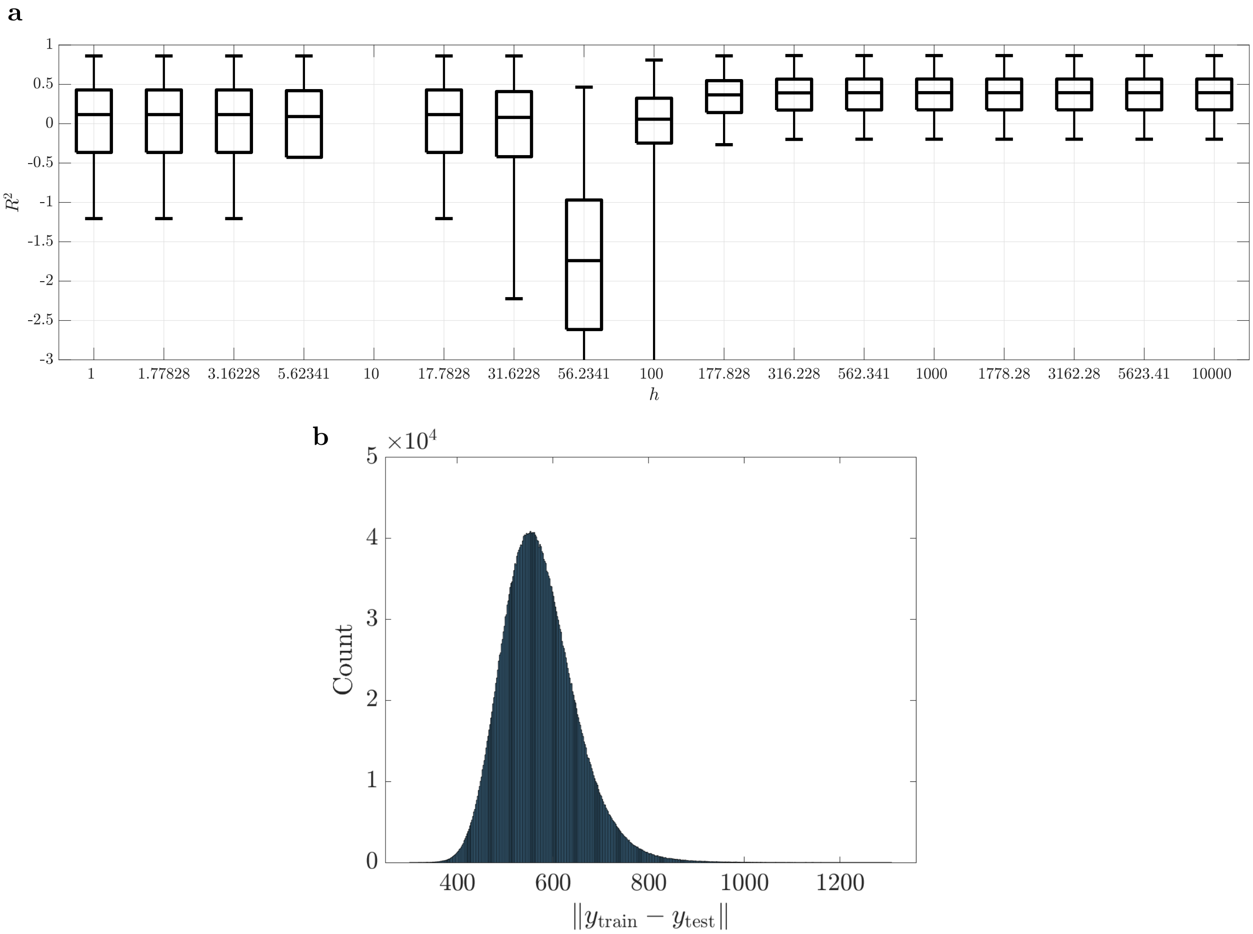

Supplementary Fig. 1 | Effect of window size h on the accuracy of the manifold-based locally linear ('Manifold') model in fMRI data. (a) Boxplot of the R^2 distribution as a function of h, combined across the 116 brain regions of 70 randomly selected subjects (10% of all subjects to reduce computational cost). The R^2 values were not computable ('NaN') for $h = 10$ due to limited machine precision. The model is equivalent to the zero model for the three leftmost boxplots as no training point falls within the Gaussian-weighted neighborhood of any test points. As h is increased to $h \sim 10$, few training data points start to fall within the neighborhood window of some of the test points, but are far enough that their Gaussian weights fall below machine precision, leading to missing ('NaN') predicted values and, hence, R^2. As h is further increased, more training points fall within the neighborhood of each test point, but are few enough to lead to poor R^2, until h is increased enough to reach the globally linear regime. (c) The distribution of the Euclidean distance between all pairs of training and test points for a randomly selected subject (subject 103818) to aid in understanding the trends that are apparent in panels (a) and (b). In particular, note that $h = 10^4$ (and even smaller values) clearly lead to a globally linear model as almost all of the training-test pairs of points have distances less than $h/10$.

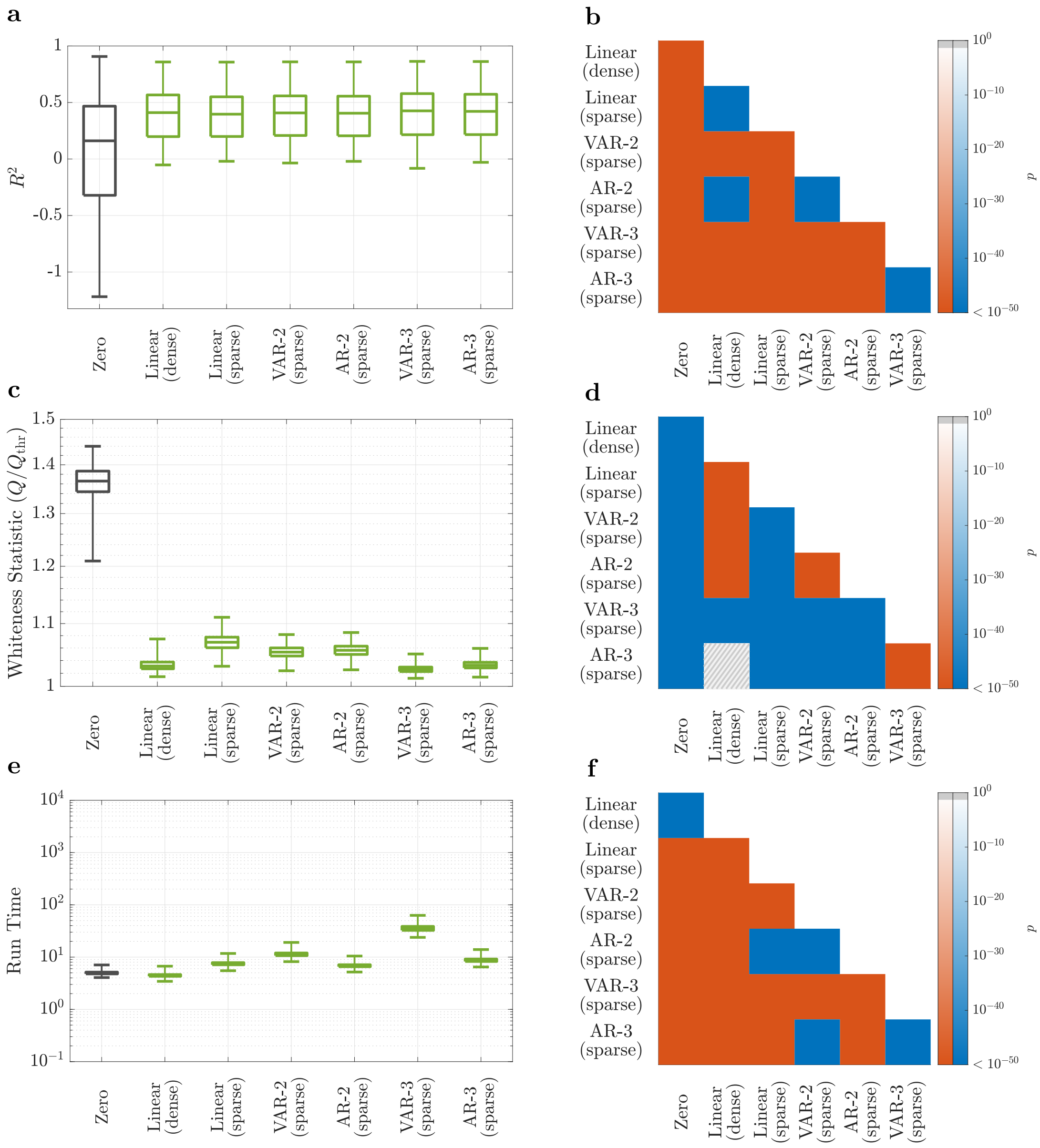

Supplementary Fig. 2 | The effects of the number of lags and sparsity patterns on the prediction accuracy and computational complexity of linear AR models of rsfMRI. Panels parallel those in **Fig. 2** in the main text and the descriptions of method acronyms are given in **Table 1** therein. Generally, the number of lags and sparsity patterns have little effect on the prediction accuracy of linear AR models for rsfMRI data (in contrast to rsiEEG data, as explained in the main text), as seen from panel (a). The estimates of statistical significance in panel (b) are to a great extent due to the large sample size (700×116). However, increasing the number of regressors, both by increasing the number of AR lags and by allowing for off-diagonal entries of all lags ('VAR' models), does lead to a non-trivial improvement in the whiteness of residuals, even though is often accompanied by non-trivial increases in model complexity and computation time as well. In all box plots, the center line, box limits, and whiskers represent the median, upper and lower quartiles, and the smallest and largest samples, respectively, and sample size $= 81200$.

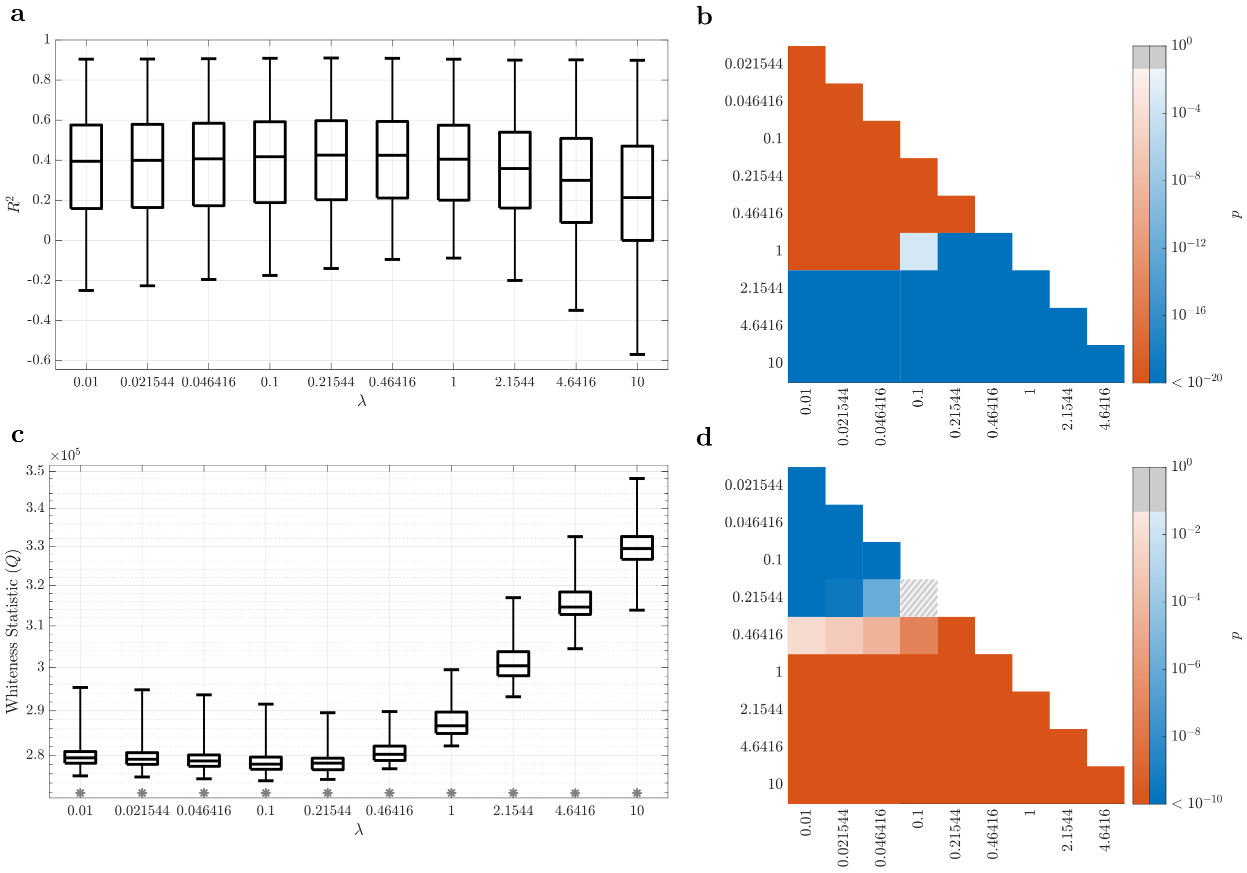

Supplementary Fig. 3 | The effect of the LASSO parameter λ on the accuracy of the 'VAR-3 (sparse)' model. Panels parallel those in **Fig. 2** of the main text. **(a)** The distribution of the cross-validated regional R_i^2, combined across all regions and 10% of subjects (randomly selected), for varying values of λ. **(b)** The p-values of the one-sided Wilcoxon signed rank test performed between all pairs of distributions of R^2 in panel **(a)**. **(c, d)** Similar to panels **(a, b)** but for the Q statistic of the test of whiteness of the residuals.

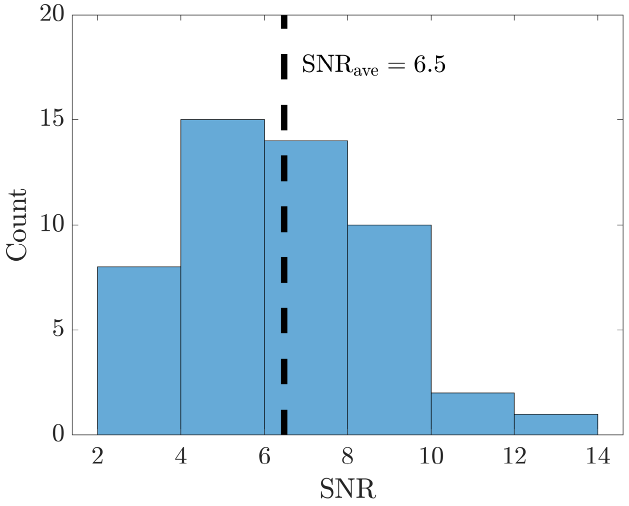

Supplementary Fig. 4 | Histogram of scanner SNR estimates for rsfMRI data. Scanner SNR was estimated for 50 randomly selected rest scans by comparing the average signal powers inside the respective subject's gray matter and outside of their head (see Methods). Due to the conservatism of this method, the resulting SNR estimates are expectedly over-estimated, but yet are not far from the SNR = 1 level that is enough to completely mask nonlinear interactions on its own (**Fig. 4g-h**).

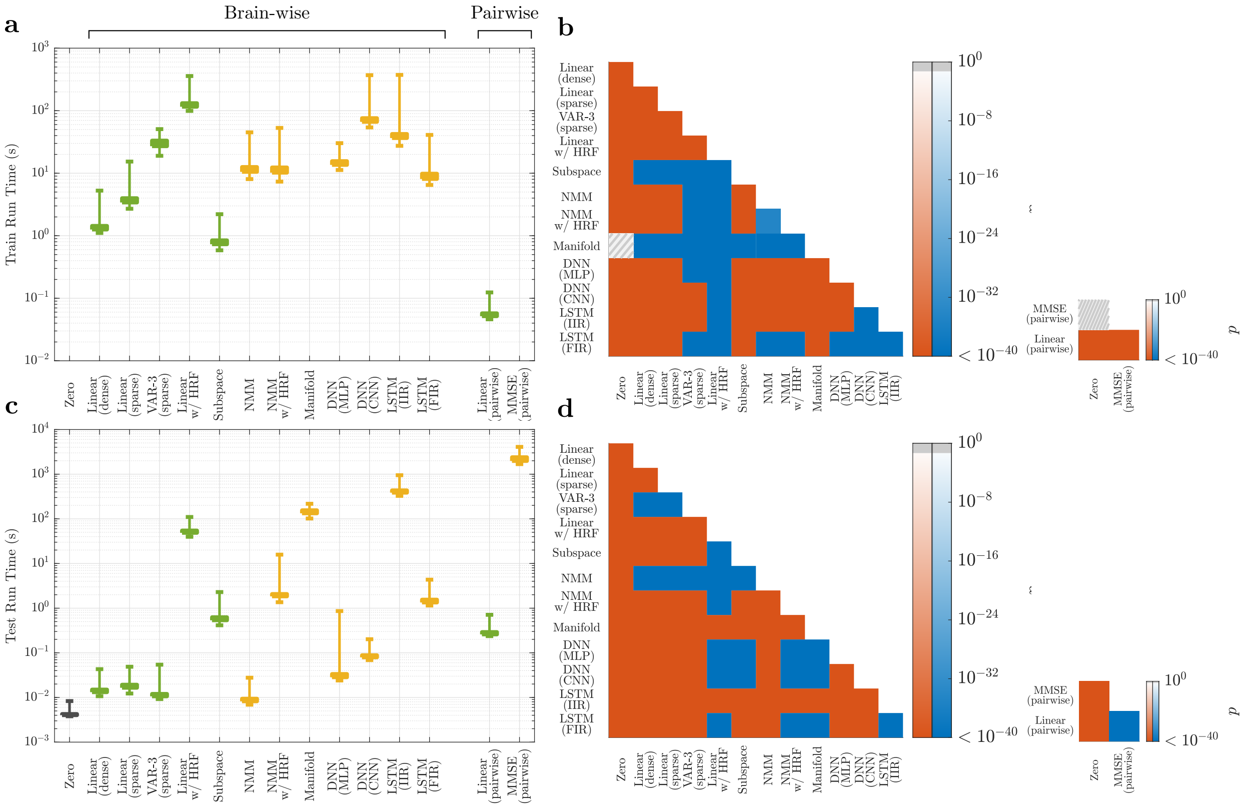

Supplementary Fig. 5 | Separate training and test times for system identification methods of rsfMRI. Details in panels (a,b) and (c,d) parallel those in of **Fig. 2e,f** in the main text. Note that the 'Zero', 'Manifold', and 'MMSE (pairwise)' methods do not have a training time by definition and hence we have set their training times uniformly to zero.

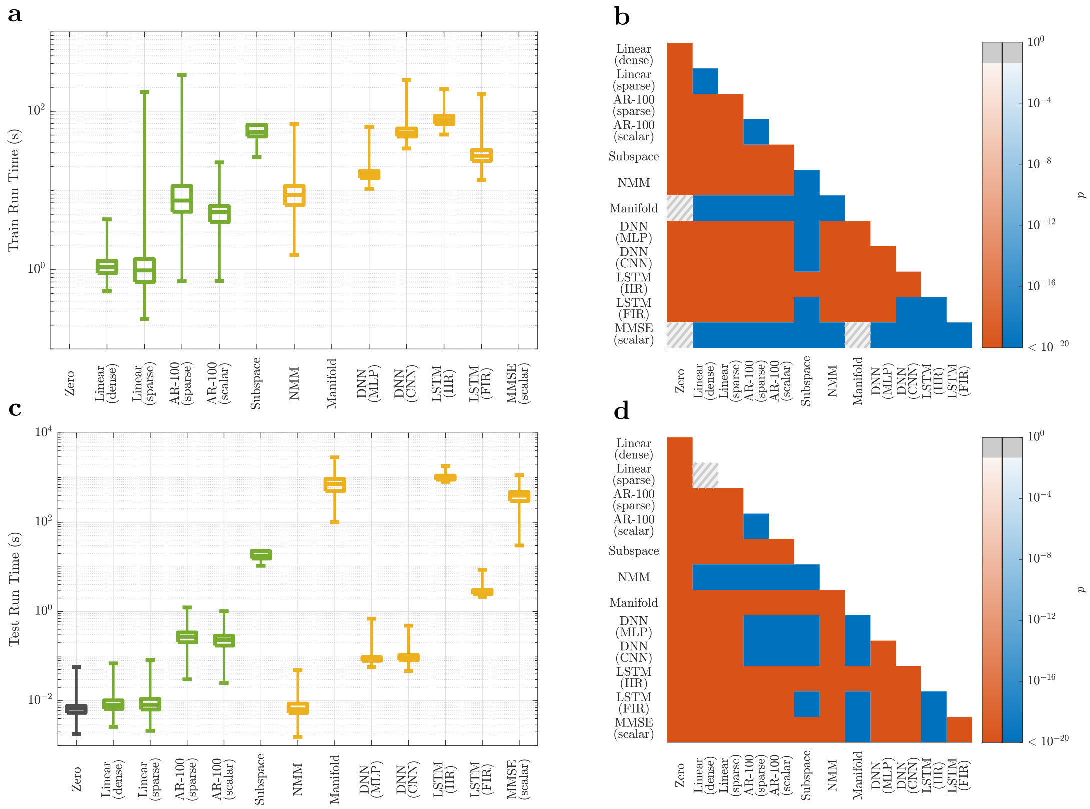

Supplementary Fig. 6 | Separate training and test times for system identification methods of rsiEEG. Details in panels (a,b) and (c,d) parallel those in of **Fig. 3e,f** in the main text. Note that the 'Zero', 'Manifold', and 'MMSE (scalar)' methods do not have a training time by definition and hence we have set their training times uniformly to zero.

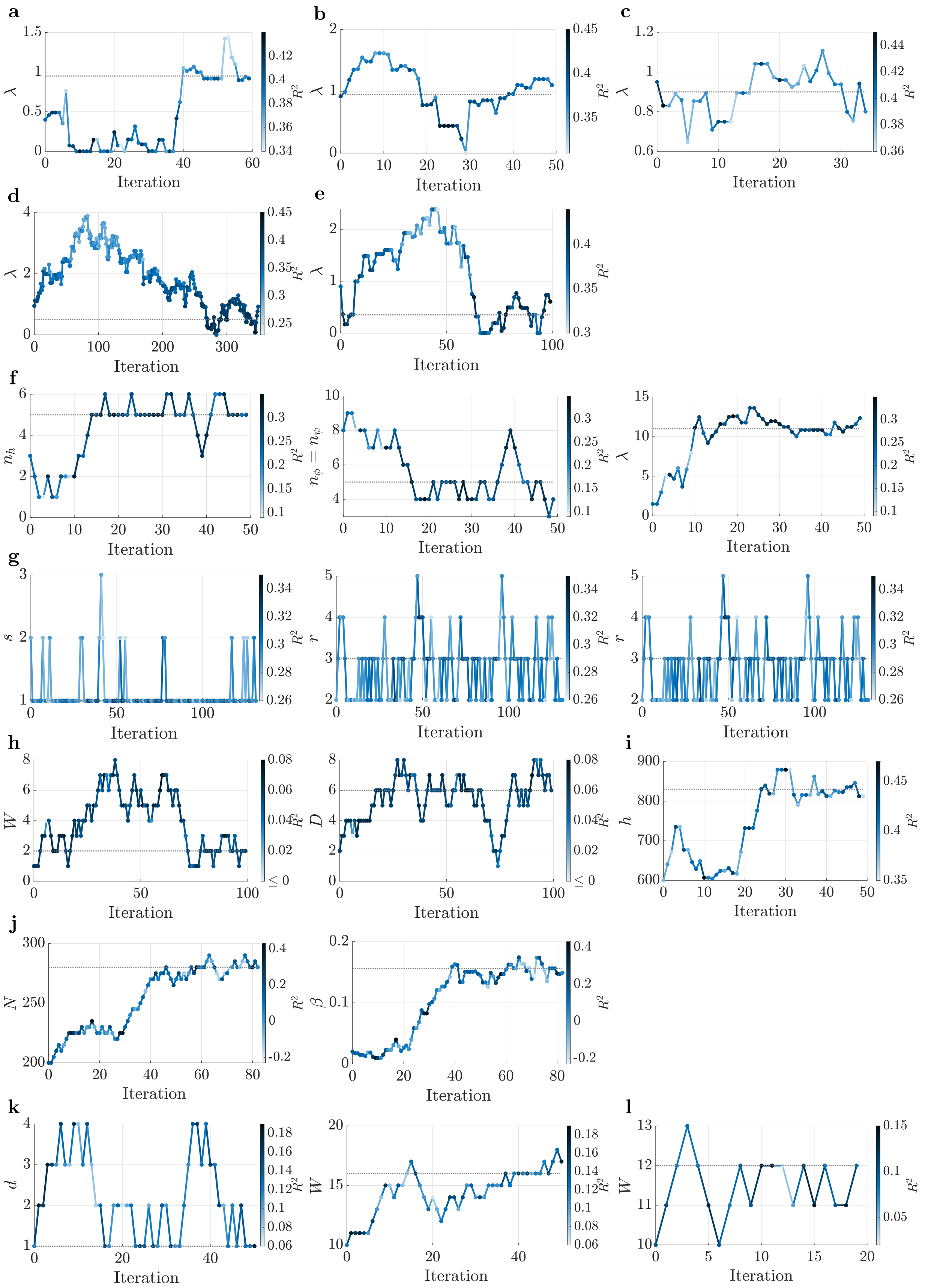

a
b
c
d
e
f
g
h
i
j
k
l
Iteration
λ
R^2
n_h
$n_\phi = n_\psi$
s
r
W
D
h
N
β
d

m

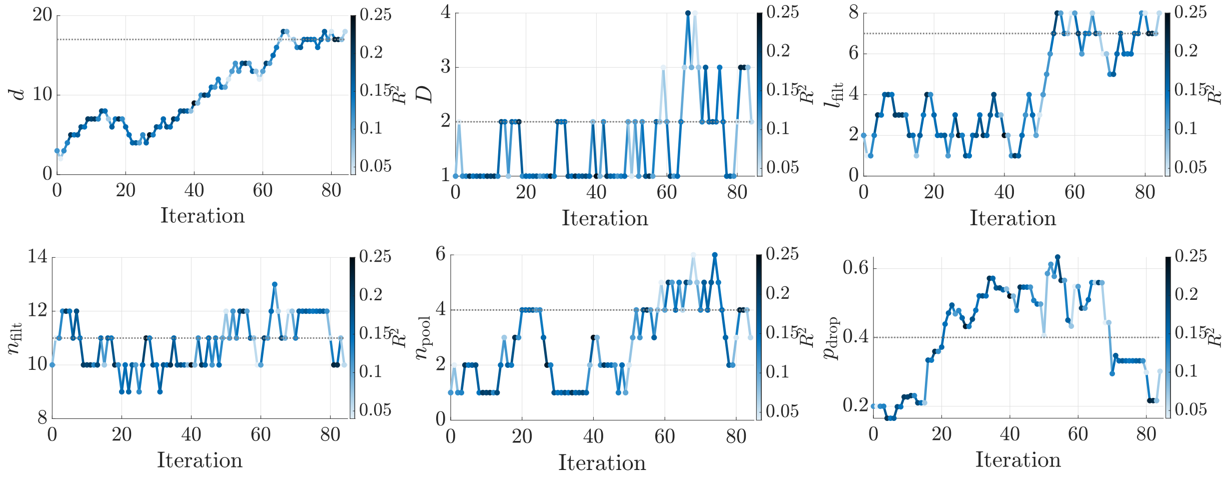

Supplementary Fig. 7 | Hyper-parameter tuning of linear and nonlinear model families for fMRI. For each parametric family of models, its hyper-parameters were simultaneously optimized using stochastic gradient descent (SGD, see Methods) to select the model with the highest cross-validated R^2 within that model family. (a) Linear (sparse); (b) AR-2 (sparse); (c) VAR-2 (sparse); (d) AR-3 (sparse); (e) VAR-3 (sparse); (f) Linear w/ HRF; (g) Subspace; (h) DNN (MLP); (i) Manifold; (j) MMSE (pairwise); (k) LSTM (FIR); (l) LSTM (IIR); (m) DNN (CNN). Each panel shows the evolution of the hyper-parameter(s) of one model family during the SGD iterations, color-coded with the value of R^2 at each iteration, and the hyper-parameter value(s) selected as optimal (dotted gray lines, also given in **Table 1** in the main text). Note that the hyper-parameter(s) are not expected to converge to a fixed (optimal) value, but rather to fluctuate around it due to the stochastic nature of SGD and the natural variability of data segments.

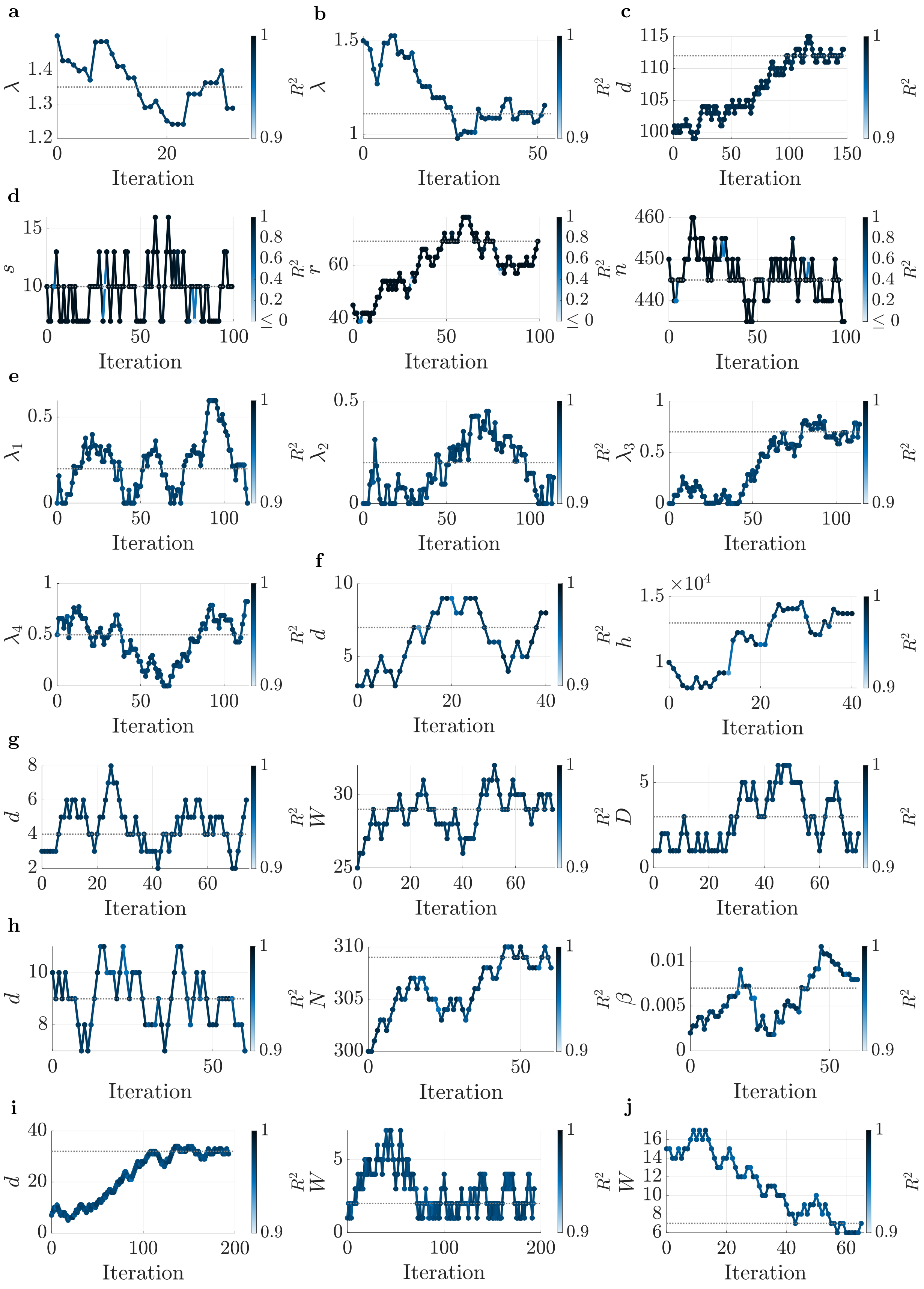
a
b
c
d
e
f
g
h
i
j
Iteration
λ
R^2
d
s
r
n
λ_1
λ_2
λ_3
λ_4
h
$\times 10^4$
W
D
N
β
≤ 0

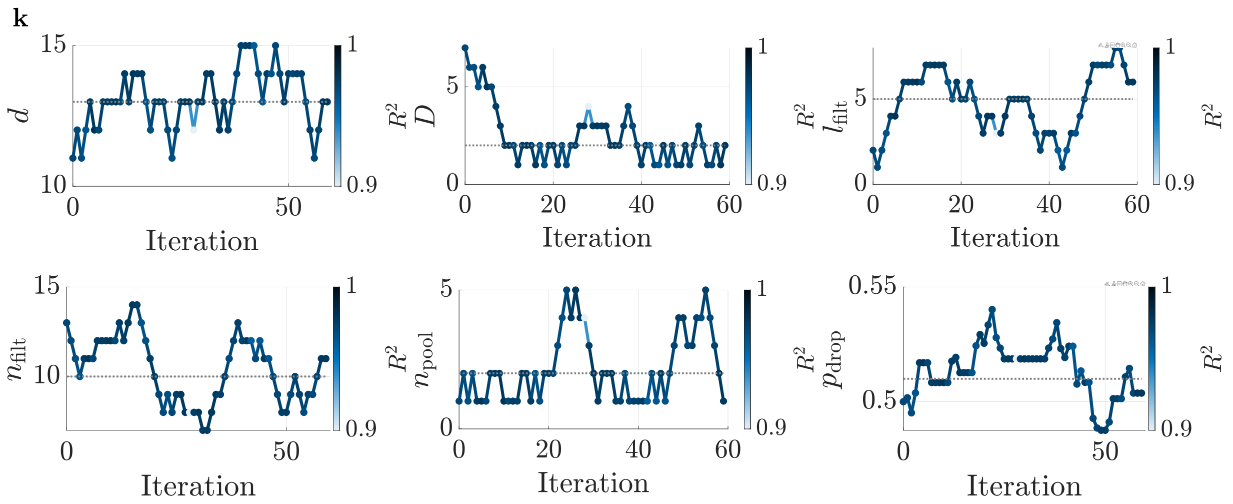

Supplementary Fig. 8 | Hyper-parameter tuning of linear and nonlinear model families for iEEG. (a) Linear (sparse); (b) AR-100 (sparse); (c) AR-100 (scalar); (d) Subspace; (e) NMM; (f) Manifold; (g) DNN; (h) MMSE (scalar); (i) LSTM (FIR); (j) LSTM (IIR); (k) DNN (CNN). Details and interpretations are the same as **Supplementary Fig. 7**.

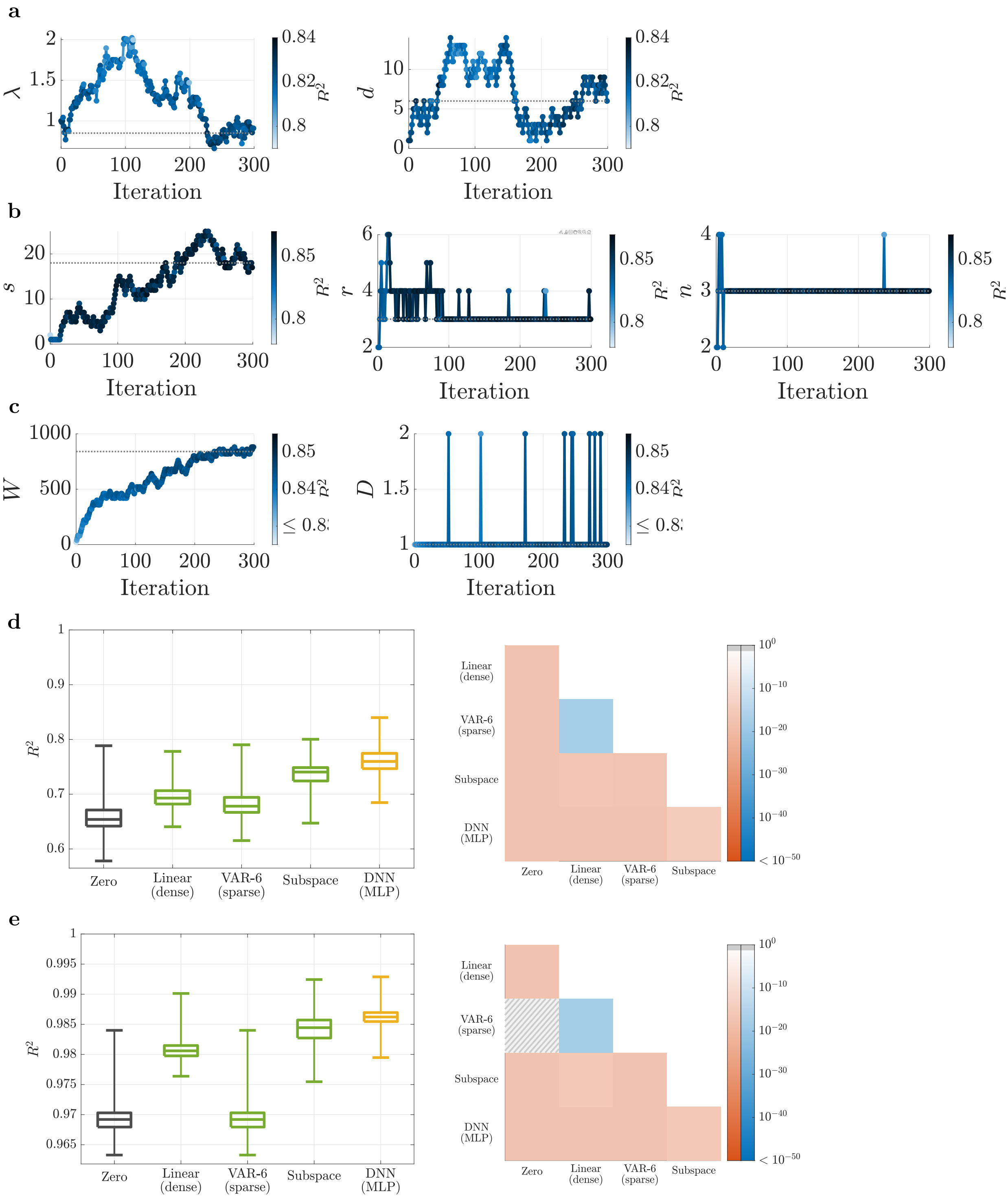

Supplementary Fig. 9 | Comparing the 'DNN (MLP)' model with linear models in fitting simulated data from the Izhikevic model. Panels (a-c) parallel those in **Supplementary Figs. 7-8** and show the resulting hyper-parameter trajectories for (a) AR-d (sparse); (b) Subspace; (c) DNN (MLP) when tuned via SGD. Panels (d,e) parallel those in **Fig. 2a** for (d) $v(t)$ and (e) $u(t)$ outputs of the Izhikevic model. Unlike **Fig. 2a** where we combined across all output channels, we here kept them separate due to the distinct form and role of their dynamics.

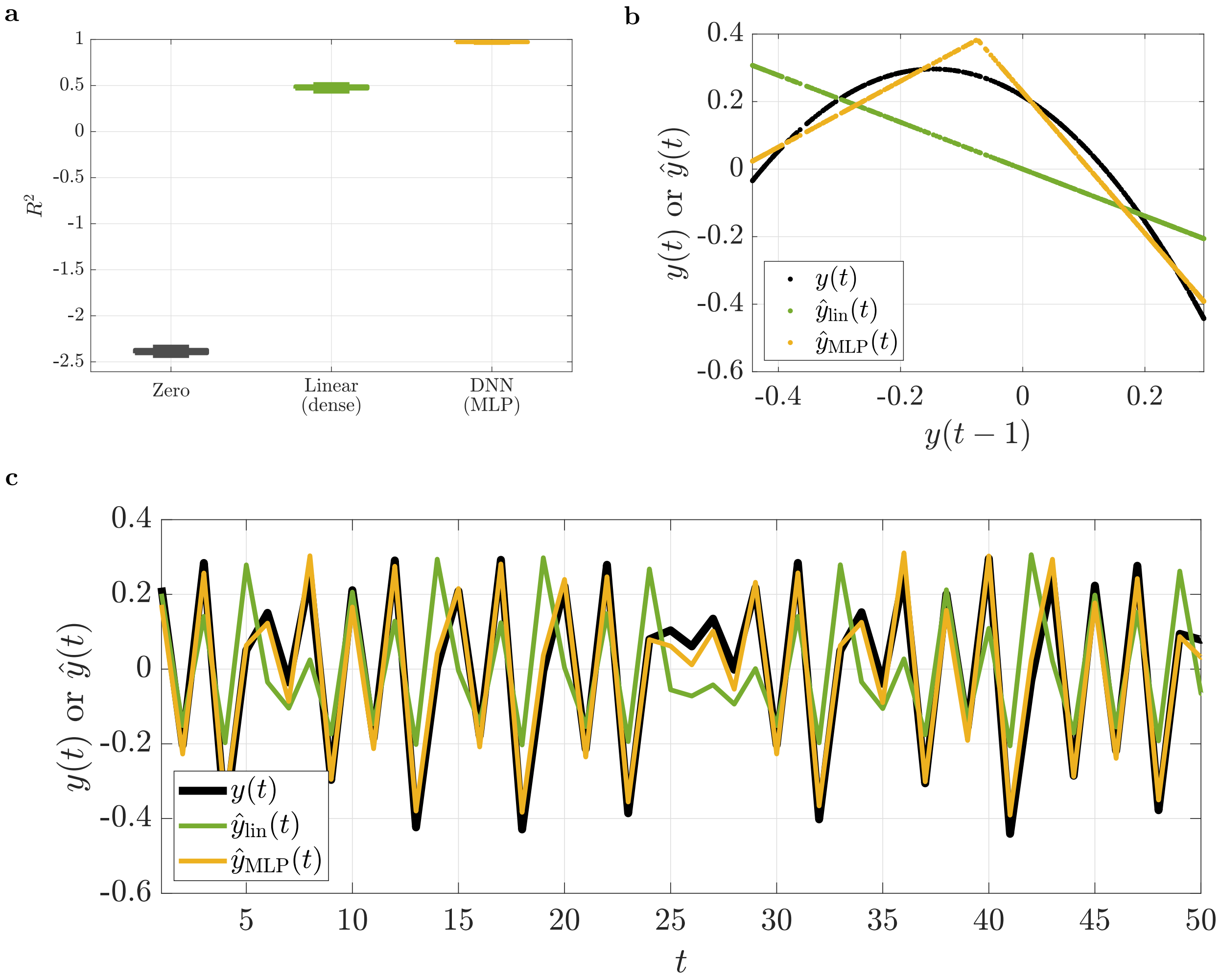

Supplementary Fig. 10 | Comparison of 'Liner (dense)' and 'DNN (MLP)' models on the logistic map dynamical system. System dynamics are given by $y(t+1) = ry(t)[1 - y(t)], r = 3.7707$. **(a)** Box plots of cross-validated one-step-ahead prediction R^2. The neural network model achieves near perfect R^2 even with 10 hidden units while the linear model achieves $R^2 \simeq 0.5$. **(b)** The approximations that each model provides to the nonlinear function $y \mapsto ry(1-y)$. **(c)** The cross-validated one-step-ahead predictions of each model for the first 50 samples of a random run.

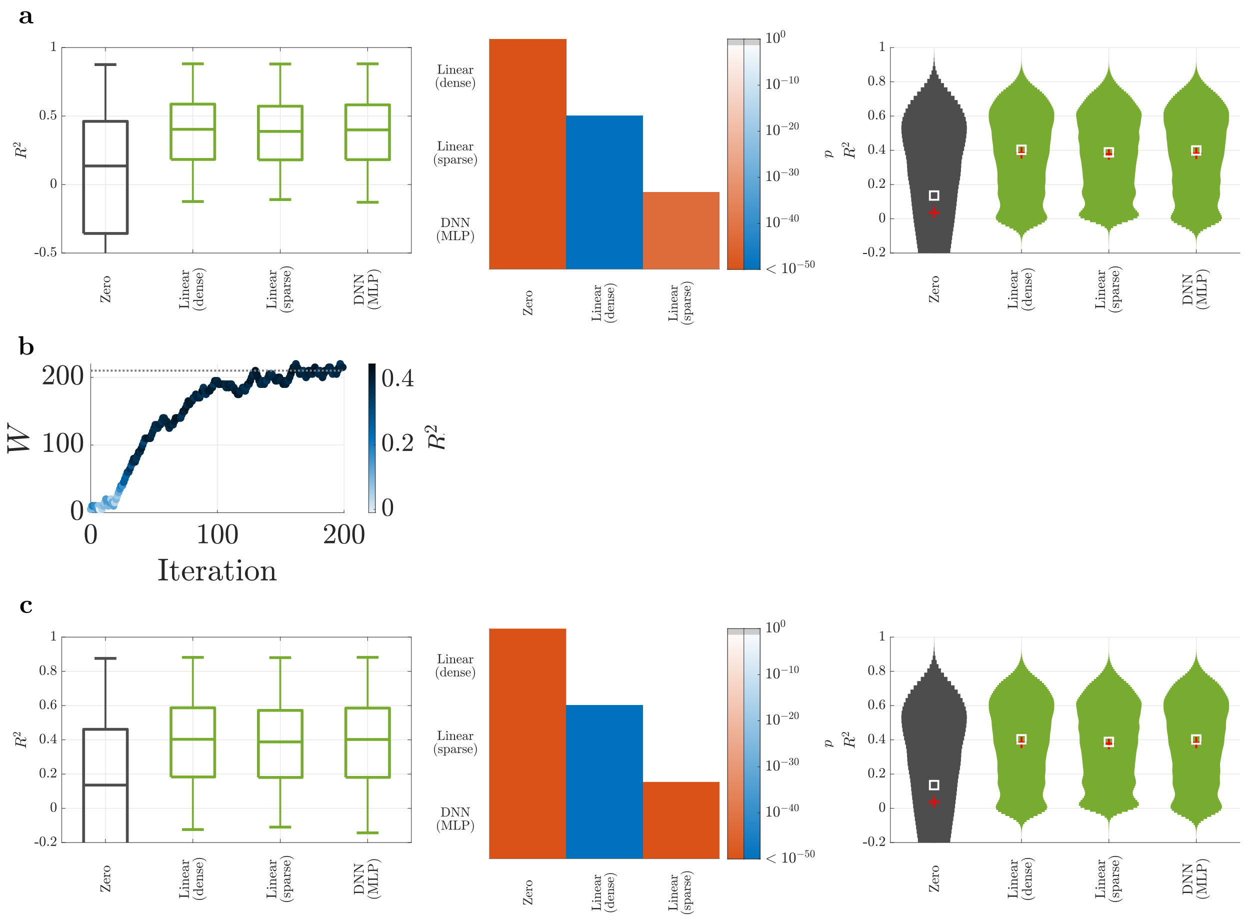

Supplementary Fig. 11 | Comparing 'DNN (MLP)' model with a linear activation function against 'Linear (dense)' and 'Linear (sparse)'. (a) The comparison results for the 'DNN (MLP)' model with a linear activation function and hidden depth of 0. The network therefore only consists of input and output layers and a fully connected layer in between. The right panel displays the same information as the left panel except for using violin plots. Red crosses and green squares show means and medians, respectively. (b) Hyper-parameter tuning for a 'DNN (MLP)' model with a linear activation function and hidden depth of 1. W denotes the width of the hidden layer. As with hyper-parameter tunings in the main text, 100 subjects whose data is not used in subsequent model comparisons are used for hyper-parameter tuning to avoid potential over-fitting to hyper-parameters. (c) Similar to (a) for but for a 'DNN (MLP)' model with a hidden depth of 1.

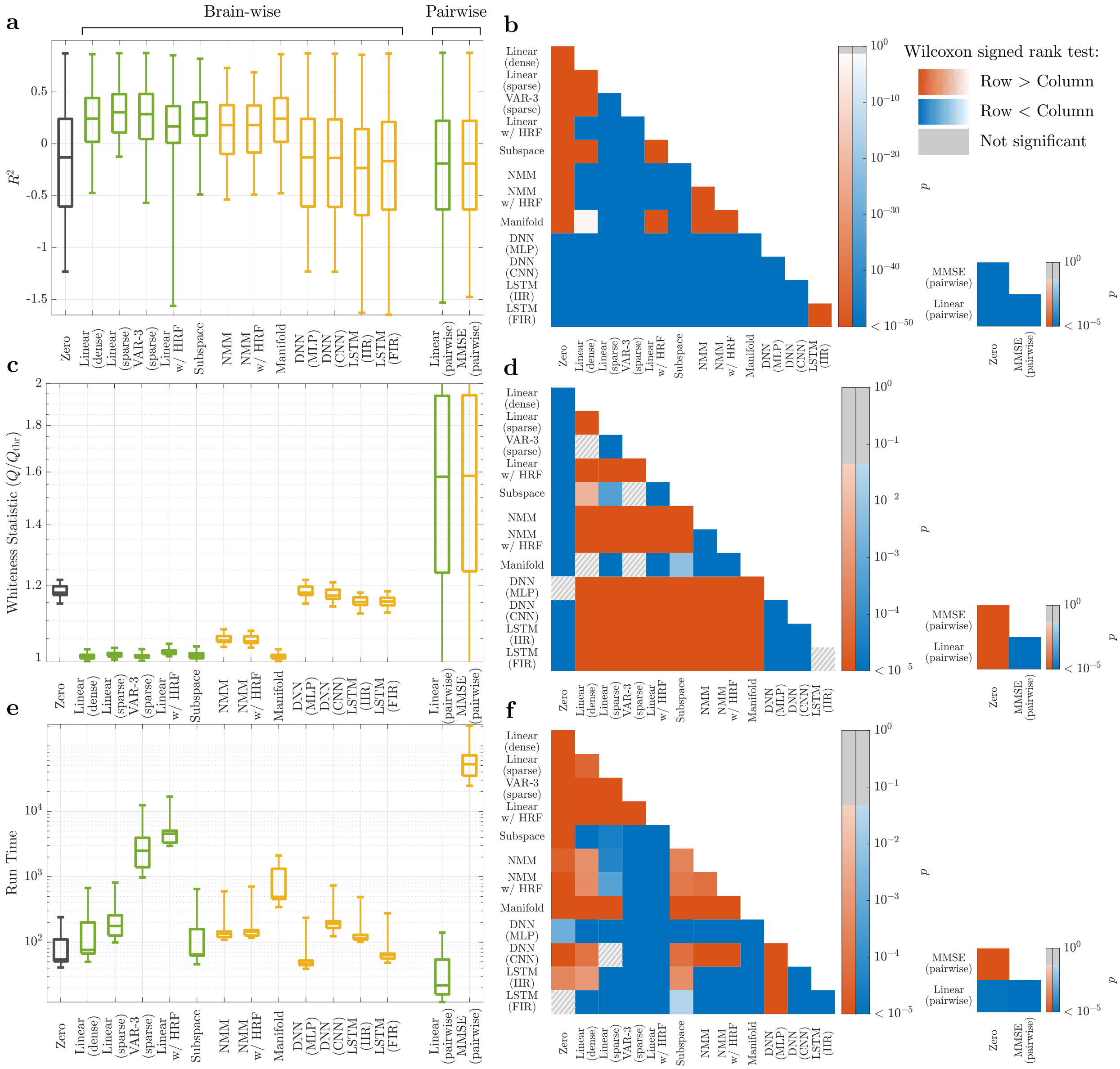

Supplementary Fig. 12 | Linear vs. nonlinear models of finely-parcellated rsfMRI activity. 400 cortical parcels (Schaefer 400x17 [4]) and 50 subcortical ones (Melbourne Scale III [5]) were used. Panels and details parallel those in **Fig. 2** in the main text, except that only data from 32 randomly selected subjects and a single-fold cross-validation has been used to reduce computational complexity. The half-session used as the test for each subject has been selected at random and the remaining 7 half-sessions have been used for training, as in the main text. The model with the highest R^2 is now the 'Linear (sparse)' even though 'VAR-3 (sparse)' still has whiter residuals.

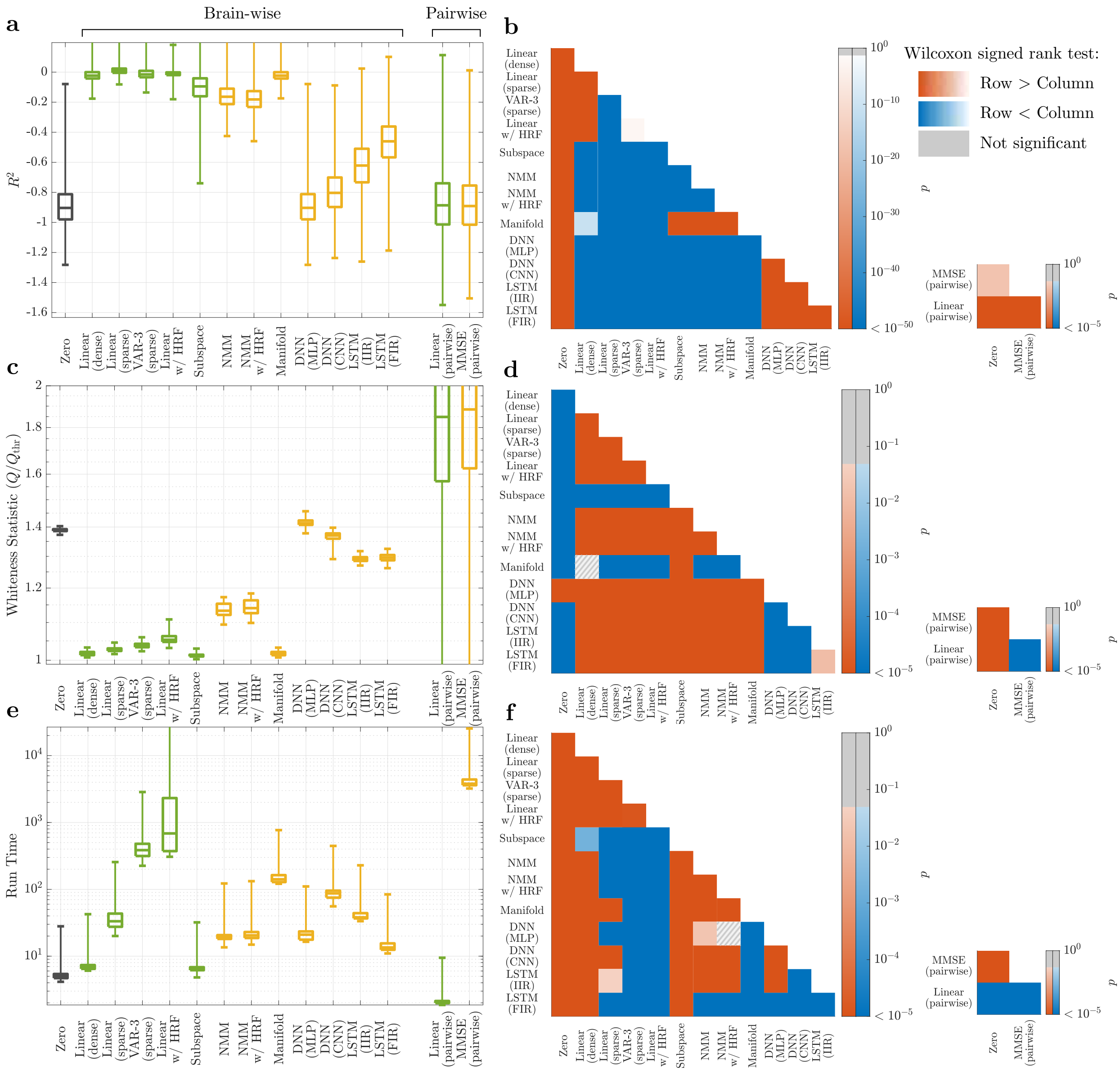
a
Brain-wise
Pairwise
R²
0
-0.2
-0.4
-0.6
-0.8
-1
-1.2
-1.4
-1.6
Zero
Linear (dense)
Linear (sparse)
VAR-3 (sparse)
Linear w/ HRF
Subspace
NMM
NMM w/ HRF
Manifold
DNN (MLP)
DNN (CNN)
LSTM (IIR)
LSTM (FIR)
Linear (pairwise)
MMSE (pairwise)
b
Wilcoxon signed rank test:
Row > Column
Row < Column
Not significant
p
c
Whiteness Statistic (Q/Q_thr)
2
1.8
1.6
1.4
1.2
1
d
e
Run Time
f

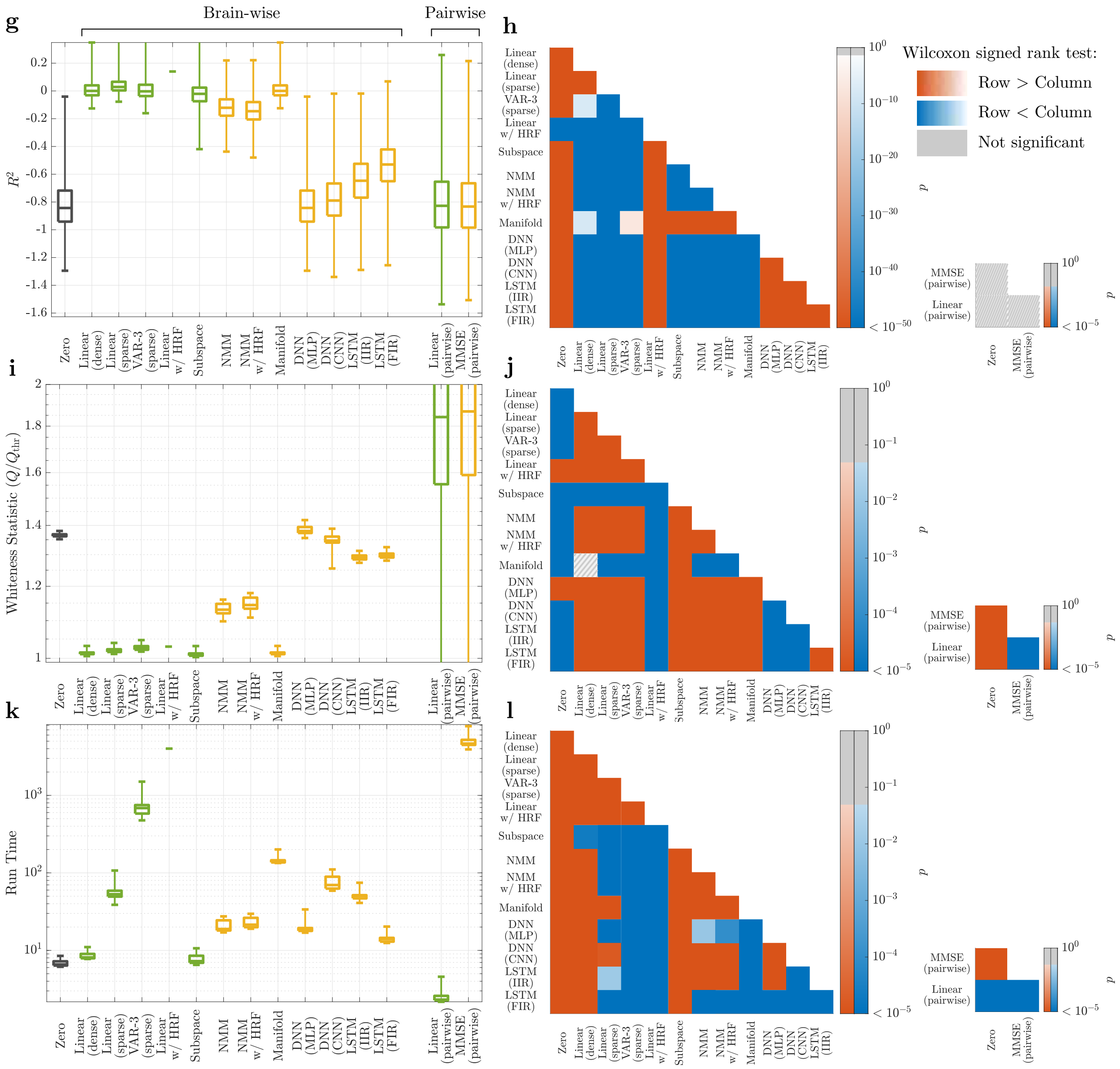

Supplementary Fig. 13 | Linear vs. nonlinear models of unparcellated cortical rsfMRI activity. To reduce computational complexity and be able to fit and validate all model families, we considered only vertices taken from two randomly selected cortical parcels: **(a-f)** a left somato-motor area '17Networks_LH_SomMotB_S2' consisting of 152 vertices, and **(g-l)** a right precuneus/posterior cingulate cortex area '17Networks_RH_DefaultA_pCunPCC_1' consisting of 177 vertices [4]). Panels and details parallel those in **Supplementary Fig. 12**.

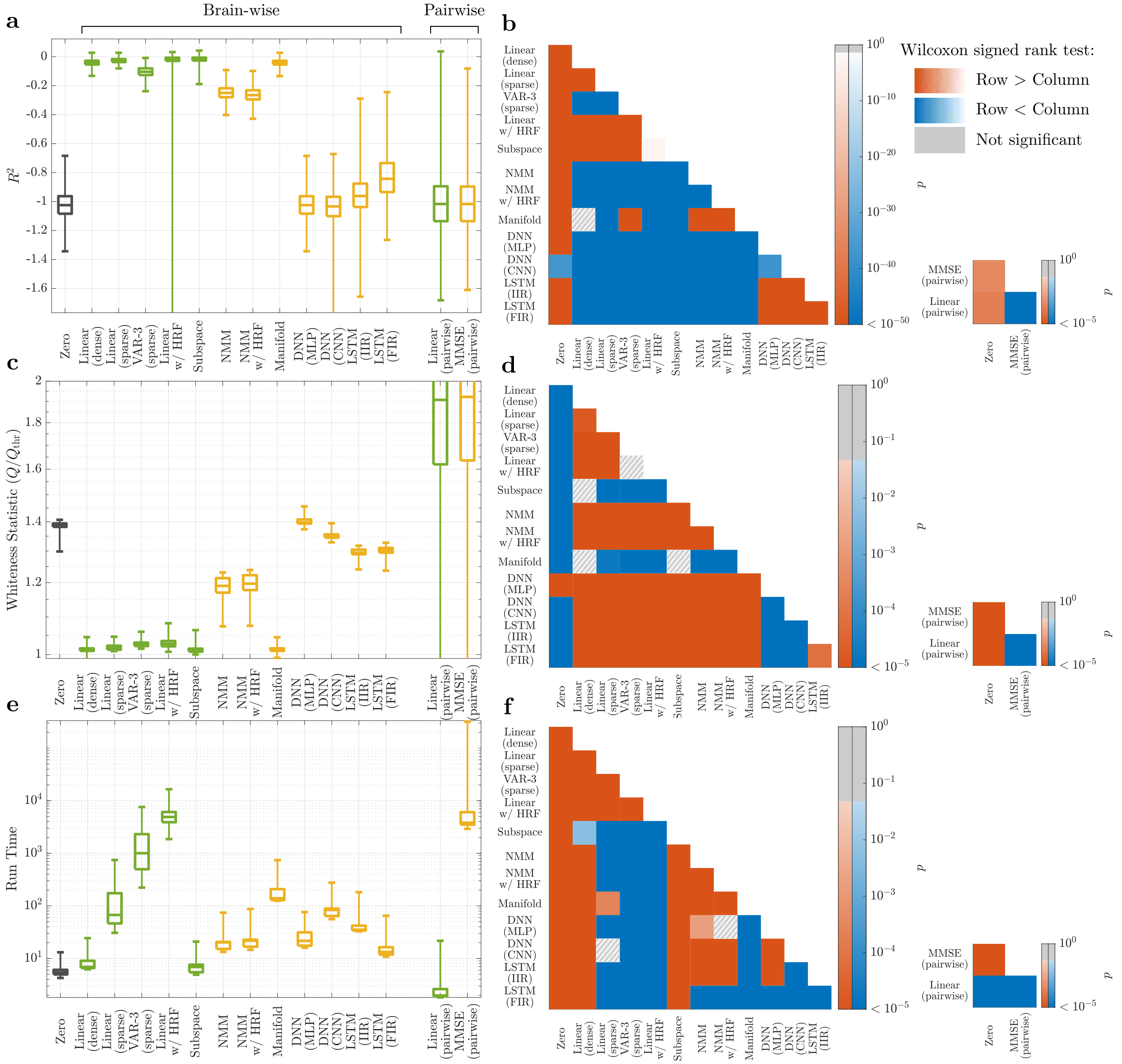

Supplementary Fig. 14 | Linear vs. nonlinear models of unparcellated subcortical rsfMRI activity. To reduce computational complexity and be able to fit and validate all model families, we considered only voxels from one randomly selected subcortical parcel (left dorsoanterior caudate, 'CAU-DA-lh' [5], consisting of 154 voxels). Panels and details parallel those in **Supplementary Fig. 12**.

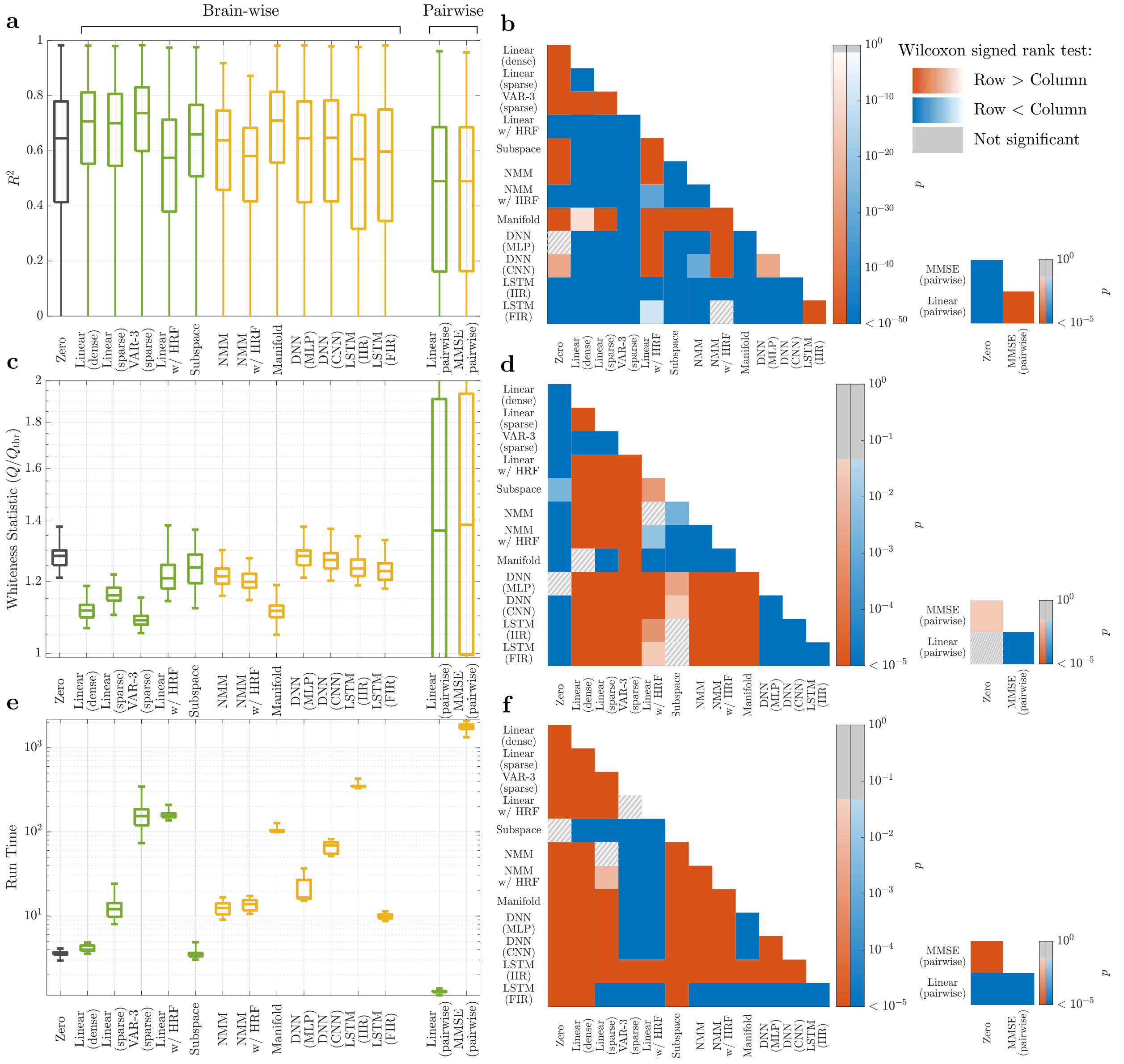

Supplementary Fig. 15 | Linear vs. nonlinear models of minimally pre-processed rsfMRI activity. Data from HCP minimally preprocessed data without ICA-FIX denoising has been used in order to ensure that the observed linearity has not stemmed from ICA-FIX. Panels parallel those in **Fig. 2** in the main text, except that data from a random selection of 10% (70) of subjects is used to reduce computational complexity.

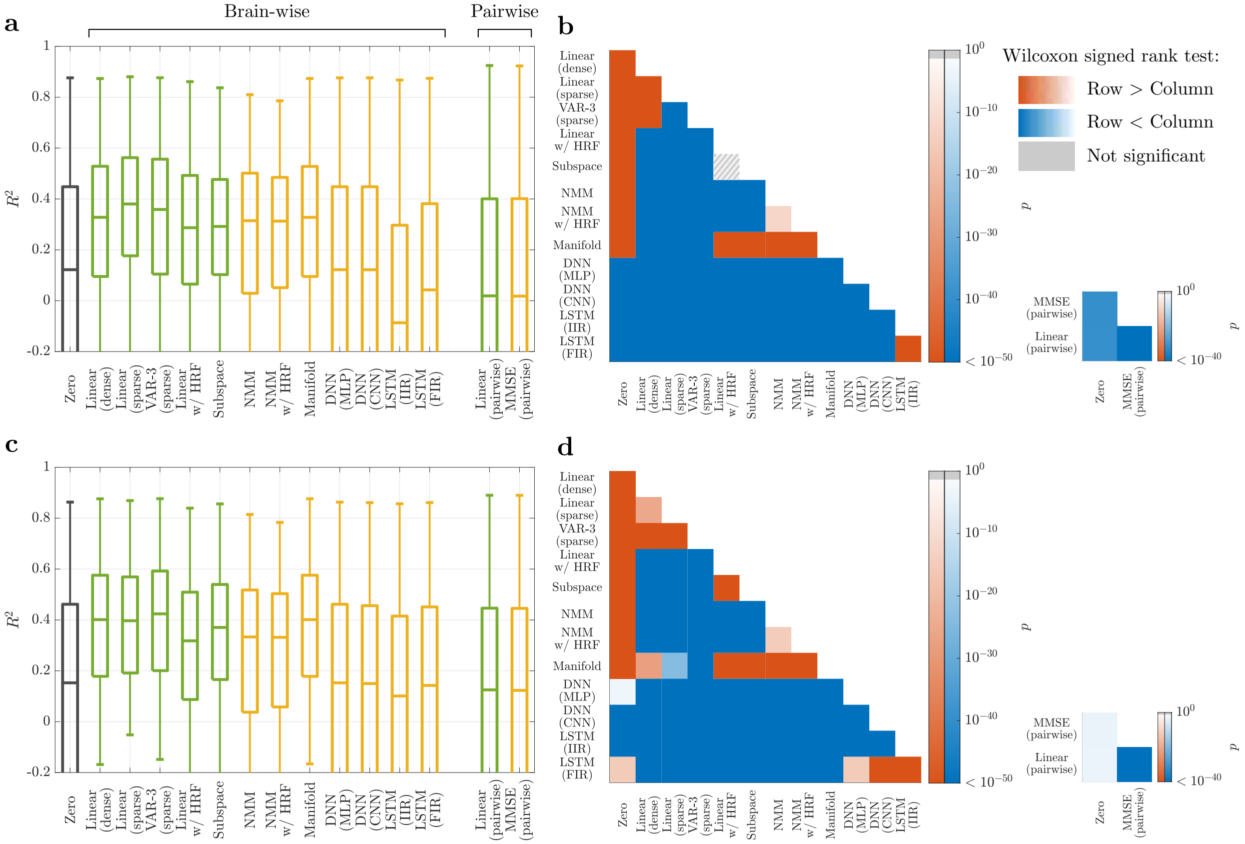

Supplementary Fig. 16 | Test-retest validation of model comparisons for rsfMRI data. Panels in each row parallel those in **Fig. 2a,b** in the main text. Panels (a,b) show the results of a session-wise approach, where the data from a single resting state session is used for both training (75%) and test (25%). Panels (c,d) show the results of a leave-one-session-out approach where 3 resting state sessions (chosen at random) from a subject are used for training and the last session from the same subject is used for test. Data from a random selection of 10% (70) of subjects is used to reduce computational complexity.

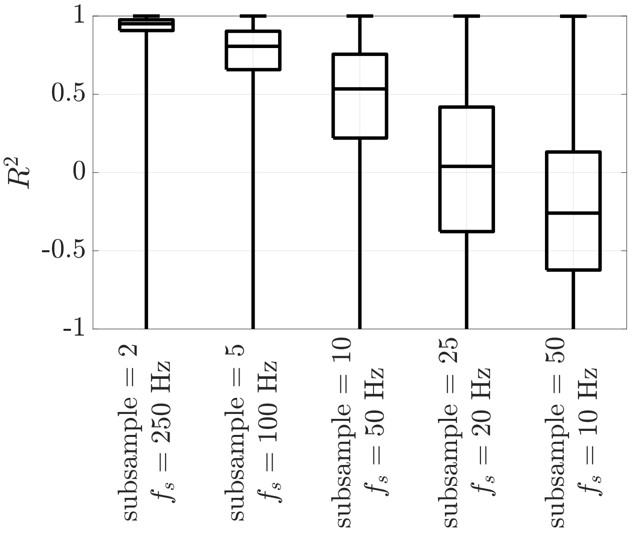

Supplementary Fig. 17 | The channel-wise R^2 distribution of the zero model for iEEG data with different subsampling ratios and the corresponding sampling frequency. As expected, higher subsampling results in less smooth time series, which in turn results in lower 'Zero' R^2, but also allows for using data from longer time intervals in model fitting and validation with the same amount of memory.

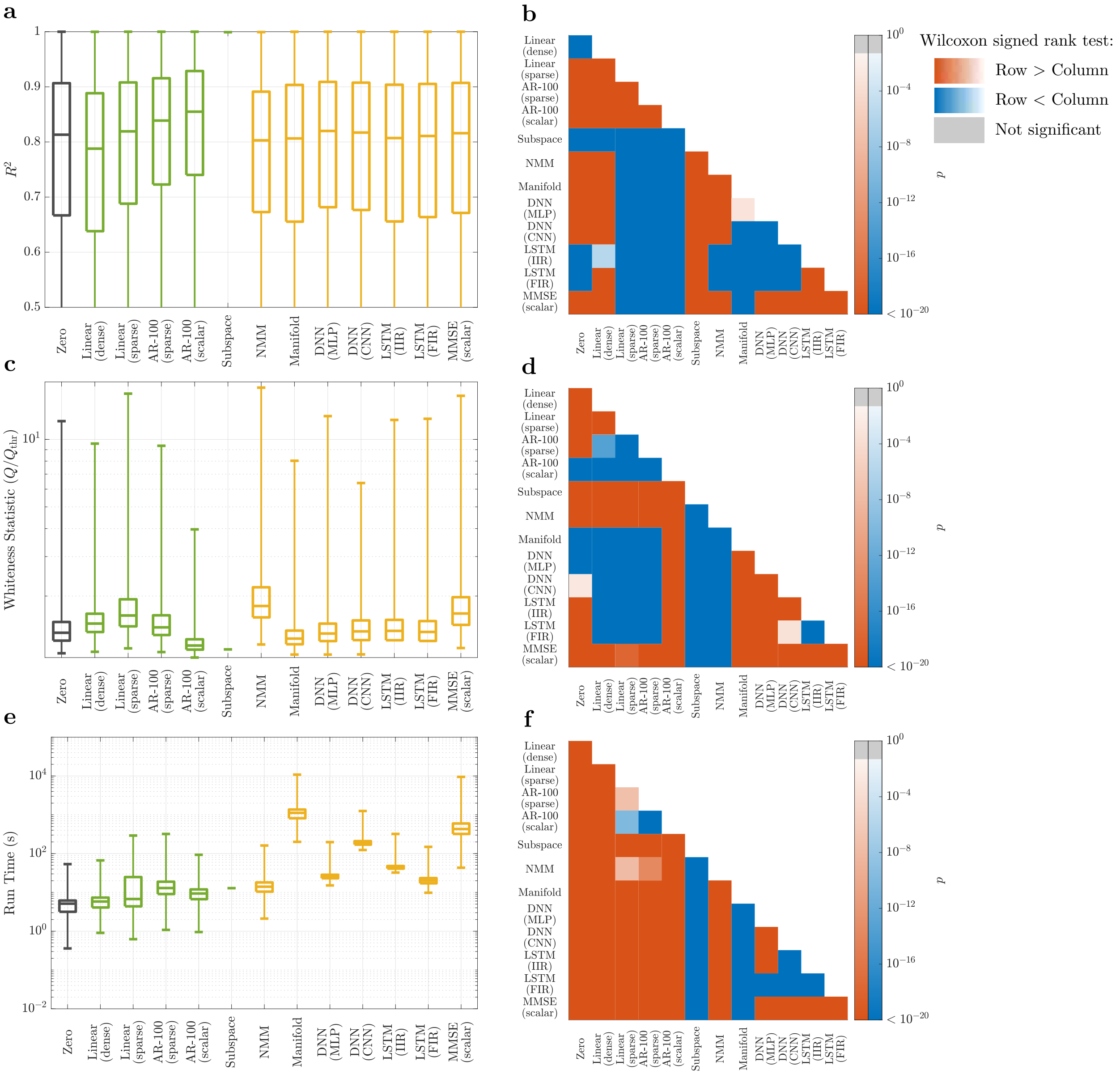

Supplementary Fig. 18 | Linear versus nonlinear models of 5-fold subsampled rsiEEG activity. Panels parallel those in **Fig. 3** in the main text. In panel (a), the box plot for the subspace method only has the top line (100th percentile) because for more than 75% of data segments the subspace method was unable to complete (either hung indefinitely or caused MATLAB to crash). For such cases we assign $R^2 = -\infty, Q = +\infty$, run time $= +\infty$ for the subspace method, causing its boxplot to miss the third quartile and anything below that. A similar situation holds in panels (c) and (e).

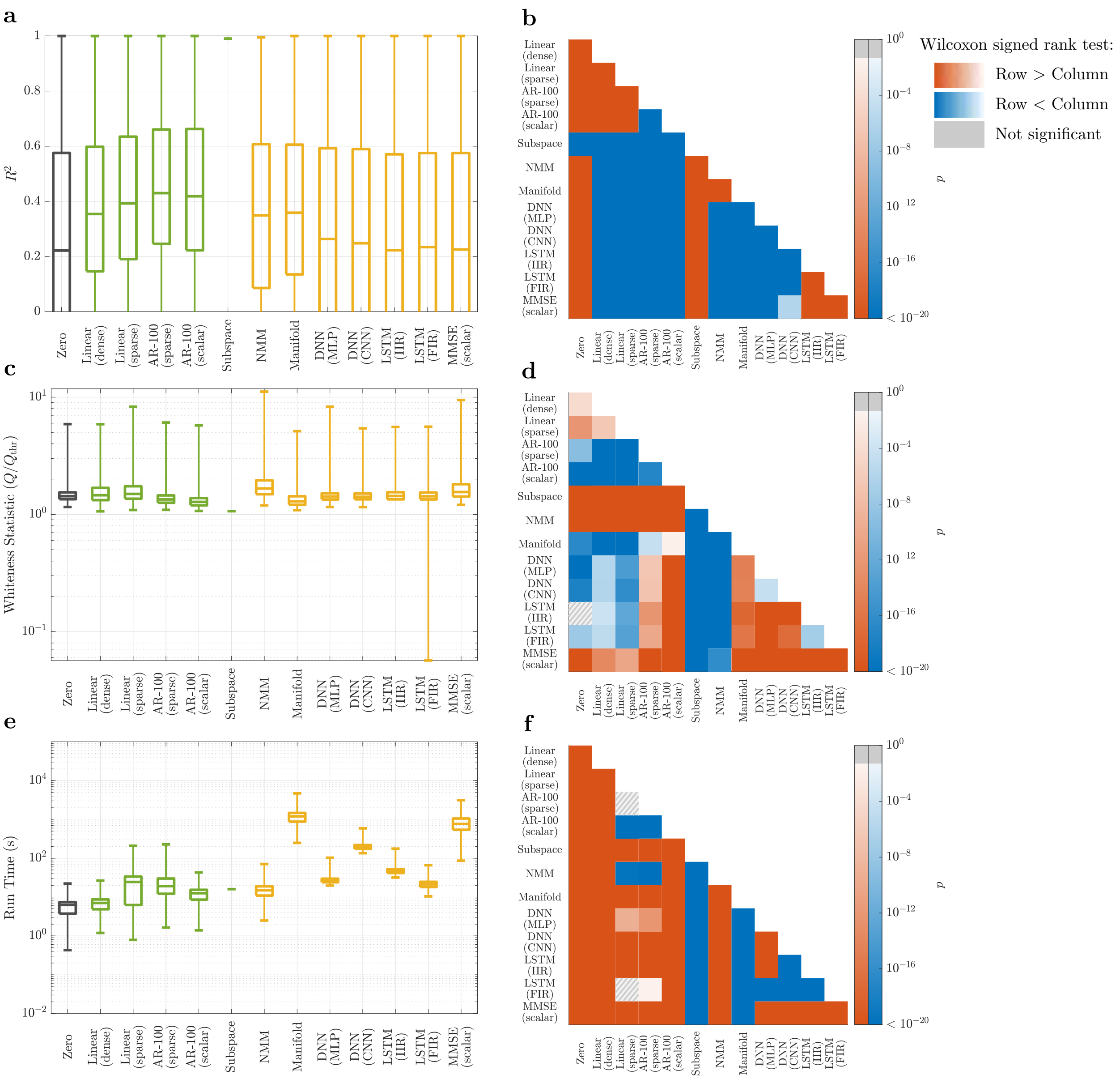

Supplementary Fig. 19 | Linear versus nonlinear models of 25-fold subsampled rsiEEG activity. Panels and details parallel those in **Supplementary Fig. 18**. Note that here the 'AR-100 (sparse)' (which includes network interactions) has the highest R^2 distribution, even though the 'AR-100 (scalar)' model still has the whitest residuals.

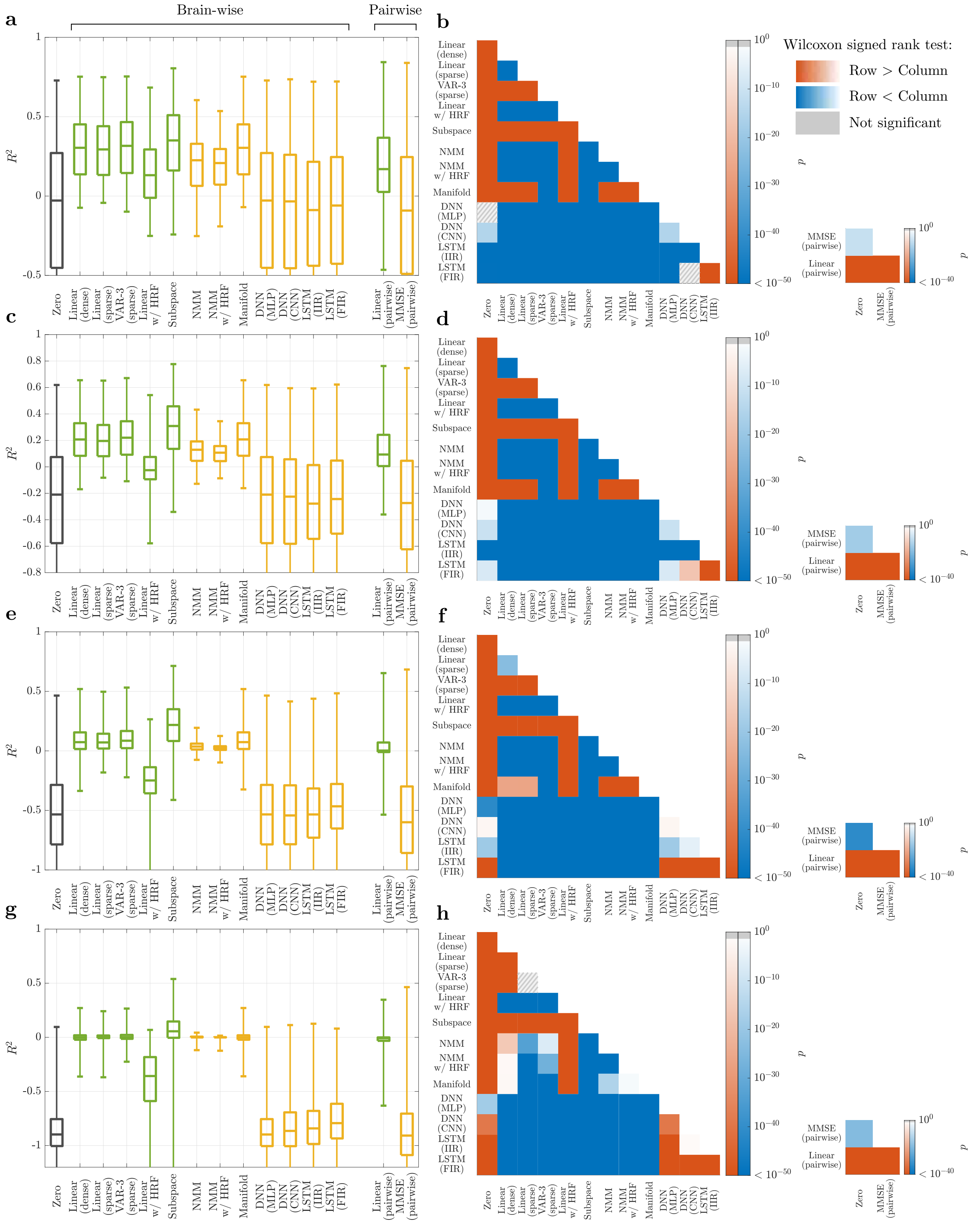

Supplementary Fig. 20 | k-step ahead prediction of rsfMRI data. Panels in each row parallel those in **Fig. 2a,b** in the main text for (a,b) $k = 2$, (c,d) $k = 3$, (e,f) $k = 5$, (g,h) $k = 10$. Data from a random selection of 10% (70) of the subjects is used to reduce computational complexity.

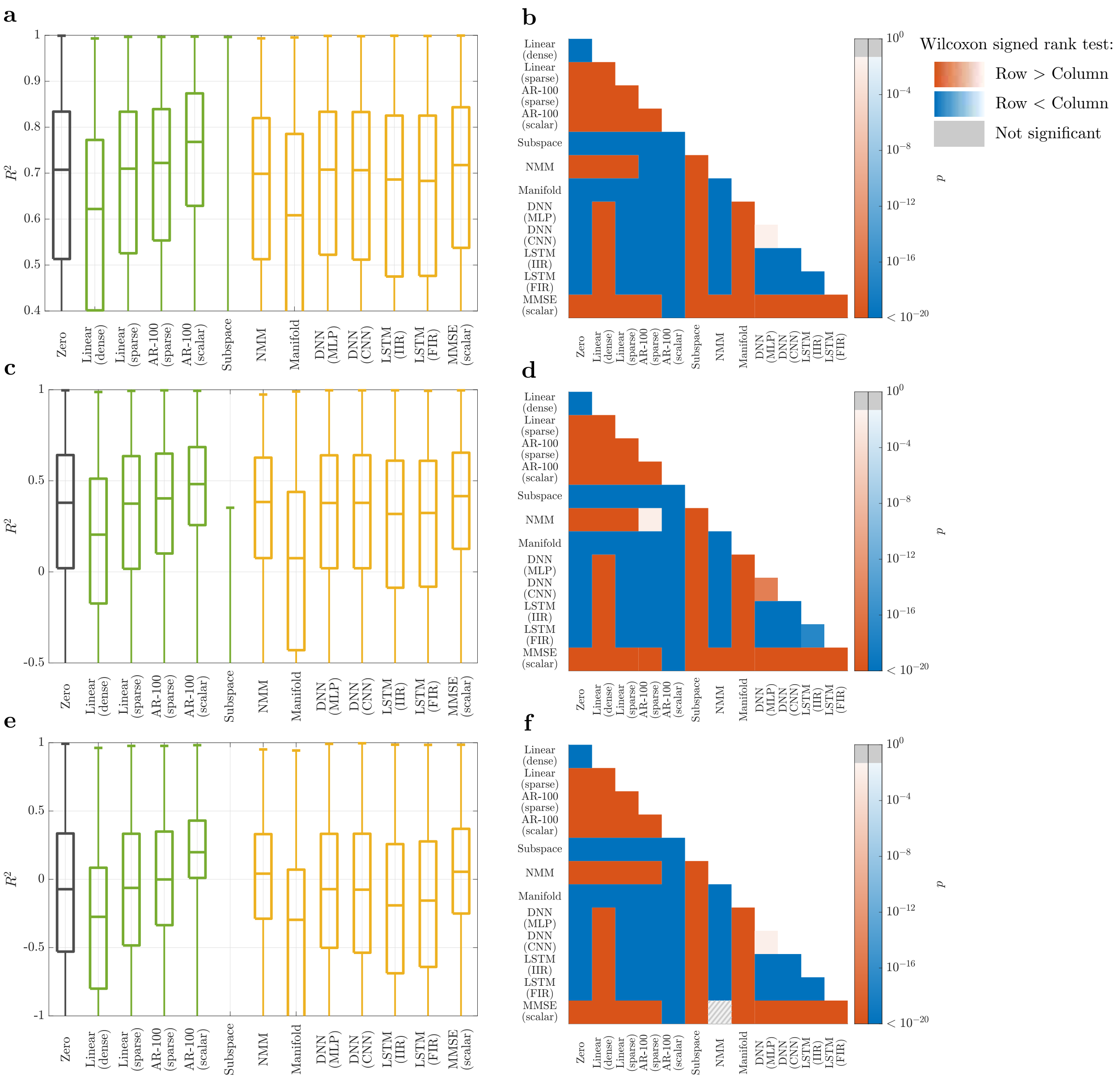

Supplementary Fig. 21 | k-step ahead prediction of rsiEEG data. Panels in each row parallel those in **Fig. 3a,b** in the main text for (a,b) $k = 5$, (c,d) $k = 10$, (e,f) $k = 20$. Data from a random selection of 84 iEEG segments (1% of the total data used in the main text) is used to reduce computational complexity.

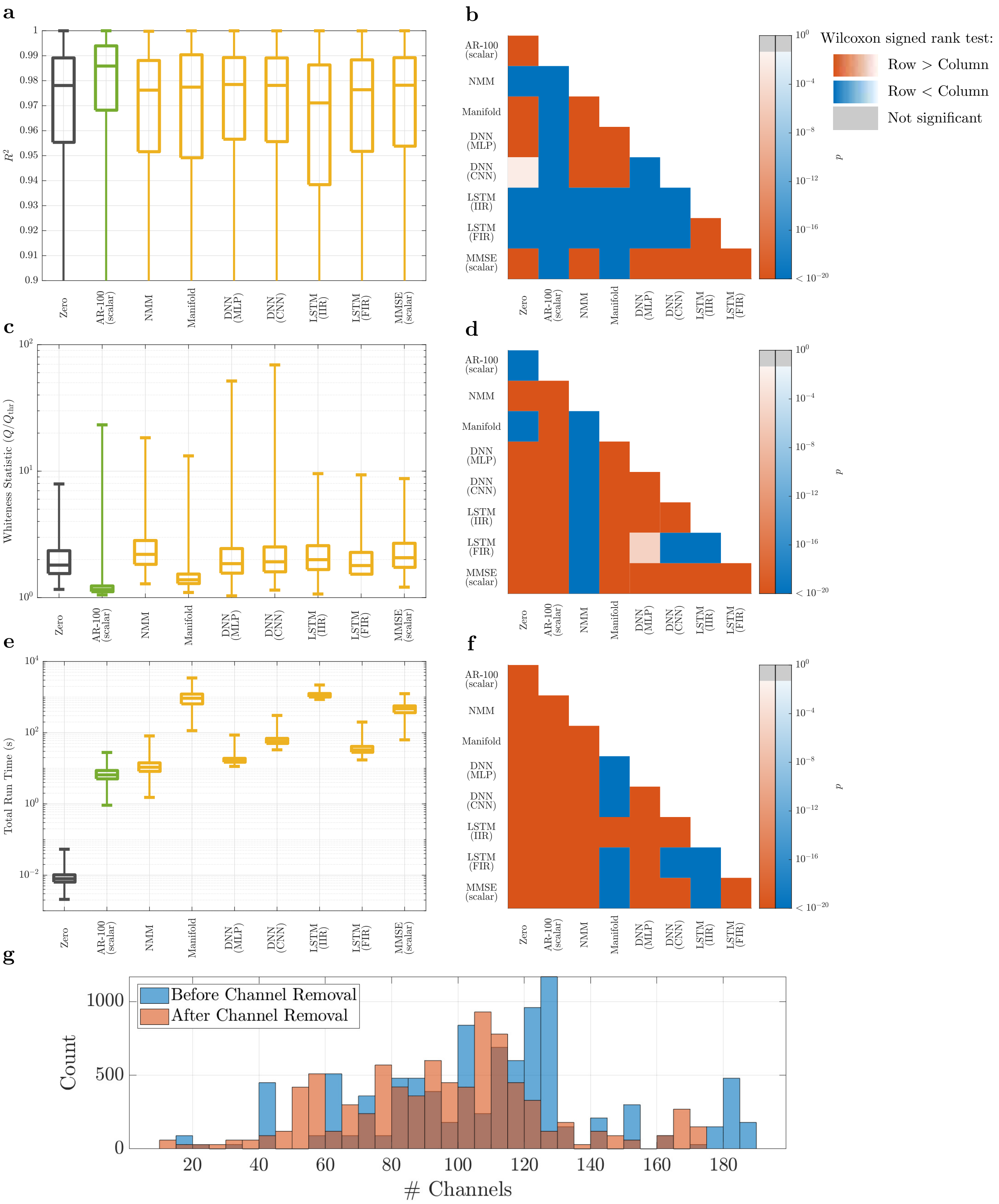

Supplementary Fig. 22 | Model comparisons on iEEG data without channel removal. Panels (a-f) parallel those in **Fig. 3** in the main text, except that here no channels are removed due to being noisy as done in the main text and explained under Methods and we only compared the top-performing linear model ('AR-100 (scalar)') with nonlinear models. The result echos the results obtained in the main text and ensures that channel removal (although performed according to standard practices and highly advisable from a data quality perspective) had not confounded the superiority of the linear models. (g) The distributions of channel counts across all subjects before and after the removal of noisy channels. The two distributions have medians 110.5 and 98 and means 108.8 and 95.4, respectively.

Supplementary References

[1] Ciric, R. *et al.* Mitigating head motion artifact in functional connectivity mri. *Nature protocols* **13**, 2801–2826 (2018).

[2] Oppenheim, A. V., Willsky, A. S. & Nawab, S. H. *Signals and Systems* Prentice-Hall signal processing series (Prentice Hall, 1997). URL `https://books.google.com/books?id=LwQqAQAAMAAJ`.

[3] Ljung, L. System identification: theory for the user. *PTR Prentice Hall, Upper Saddle River, NJ* 1–14 (1999).

[4] Schaefer, A. *et al.* Local-global parcellation of the human cerebral cortex from intrinsic functional connectivity mri. *Cerebral Cortex* **28**, 3095–3114 (2018).

[5] Tian, Y., Margulies, D. S., Breakspear, M. & Zalesky, A. Hierarchical organization of the human subcortex unveiled with functional connectivity gradients. *bioRxiv* (2020).
