# Supplementary material for: Macroscopic resting-state brain dynamics are best described by linear models
Source: Nat Biomed Eng. 2023 Dec 11;8(1):68–84. doi: 10.1038/s41551-023-01117-y (PMC11357987; doi:10.1038/s41551-023-01117-y)
Supplement: Supplementary file 2 — Reporting Summary [file 41551_2023_1117_MOESM2_ESM.pdf]

Corresponding author(s): Dr. Danielle S. Bassett

Last updated by author(s): Aug 16, 2023

Reporting Summary

Nature Portfolio wishes to improve the reproducibility of the work that we publish. This form provides structure for consistency and transparency in reporting. For further information on Nature Portfolio policies, see our Editorial Policies and the Editorial Policy Checklist.

Statistics

For all statistical analyses, confirm that the following items are present in the figure legend, table legend, main text, or Methods section.

n/a	Confirmed	
☐	☒	The exact sample size (*n*) for each experimental group/condition, given as a discrete number and unit of measurement
☒	☐	A statement on whether measurements were taken from distinct samples or whether the same sample was measured repeatedly
☐	☒	The statistical test(s) used AND whether they are one- or two-sided<br>*Only common tests should be described solely by name; describe more complex techniques in the Methods section.*
☒	☐	A description of all covariates tested
☐	☒	A description of any assumptions or corrections, such as tests of normality and adjustment for multiple comparisons
☒	☐	A full description of the statistical parameters including central tendency (e.g. means) or other basic estimates (e.g. regression coefficient) AND variation (e.g. standard deviation) or associated estimates of uncertainty (e.g. confidence intervals)
☐	☒	For null hypothesis testing, the test statistic (e.g. *F*, *t*, *r*) with confidence intervals, effect sizes, degrees of freedom and *P* value noted<br>*Give P values as exact values whenever suitable.*
☒	☐	For Bayesian analysis, information on the choice of priors and Markov chain Monte Carlo settings
☒	☐	For hierarchical and complex designs, identification of the appropriate level for tests and full reporting of outcomes
☒	☐	Estimates of effect sizes (e.g. Cohen's *d*, Pearson's *r*), indicating how they were calculated

Our web collection on statistics for biologists contains articles on many of the points above.

Software and code

Policy information about availability of computer code

Data collection	No software was used.
Data analysis	Custom MATLAB software, executed in MATLAB R2018a, was used for data pre-processing and for all analyses in the manuscript. All the MATLAB code used for pre-processing and data analysis are publicly available on Github at https://github.com/enozari/rest-system-id. A few publicly available MATLAB packages were also used within this custom code, as described in the README file in the Github repository.

For manuscripts utilizing custom algorithms or software that are central to the research but not yet described in published literature, software must be made available to editors and reviewers. We strongly encourage code deposition in a community repository (e.g. GitHub). See the Nature Portfolio guidelines for submitting code & software for further information.

Data

Policy information about availability of data

All manuscripts must include a data availability statement. This statement should provide the following information, where applicable:

- Accession codes, unique identifiers, or web links for publicly available datasets
- A description of any restrictions on data availability
- For clinical datasets or third party data, please ensure that the statement adheres to our policy

The fMRI and iEEG data supporting the findings of this study are publicly available, respectively, from the HCP S1200 Release at https://

www.humanconnectome.org/study/hcp-young-adult/ document/1200-subjects-data-release, and the RAM Public Data Release at http://memory.psych.upenn.edu/RAM.

Research involving human participants, their data, or biological material

Policy information about studies with human participants or human data. See also policy information about sex, gender (identity/presentation), and sexual orientation and race, ethnicity and racism.

Reporting on sex and gender	Data from all available participants in the HCP and RAM datasets were used regardless of sex and gender. The sex and gender of the participants were not used in any of the analyses, as sex and gender differences were not within the scope of this project; however, sex and gender information can be extracted from the respective public datasets. This study analysed the degree to which macroscopic brain dynamics are linear across both sexes and genders.
Reporting on race, ethnicity, or other socially relevant groupings	No socially constructed or socially relevant categorization variables were used. Same analysis routines were applied to all available participants regardless of their race, ethnicity, or other socially relevant groupings.
Population characteristics	No covariate analysis was performed.
Recruitment	We only used data from publicly available and well-cited datasets (HCP and RAM). Participants were recruited as described in the respective dataset descriptions.
Ethics oversight	The HCP experiments were carried out by the WU-Minn consortium and its adherence to ethical standards was approved by the Internal Review Board of the respective institutions. Explicit informed consent was acquired from all participants involved in the study.

Note that full information on the approval of the study protocol must also be provided in the manuscript.

Field-specific reporting

Please select the one below that is the best fit for your research. If you are not sure, read the appropriate sections before making your selection.

☒ Life sciences ☐ Behavioural & social sciences ☐ Ecological, evolutionary & environmental sciences

For a reference copy of the document with all sections, see nature.com/documents/nr-reporting-summary-flat.pdf

Life sciences study design

All studies must disclose on these points even when the disclosure is negative.

Sample size	700 participants in the fMRI analysis, 122 participants in the iEEG analysis. These sample sizes were the maximum available from either dataset, and were far more than statistically needed (as indicated in the comparison p-value tables in Figs. 2,3, where almost all p-values fall below 1e-20 for iEEG and 1e-40 for fMRI).
Data exclusions	fMRI data: we removed participants from further analysis if any of their four resting scans had excessively large head motion, defined by having frames with greater than 0.2 mm frame-wise displacement or a derivative root mean square (DVARS) above 75. Also, participants listed in [Elam, "Hcp data release updates: Known issues and planned fixes", 2020] under ``3T Functional Preprocessing Error of all 3T RL fMRI runs in 25 Subjects" or ``Subjects without Field Maps for Structural scans" were removed.<br><br>iEEG data: For all participants, we rejected noisy channels that were either (i) marked as noisy in the RAM dataset notes, (ii) had a line length greater then three times the mean, (iii) had z-scored kurtosis greater than 1.5, or (iv) had a z-scored power-spectral density dissimilarity measure greater than 1.5. The dissimilarity measure used was the average of one minus the Spearman's rank correlation with all channels.<br><br>All exclusion criteria are minimal and were pre-established.
Replication	Cross-validation was used so that the performance of all included models was tested on data not seen during training.
Randomization	No group allocation was performed or was applicable. All models were applied to all the data segments given the computational nature of the study, so no randomization was performed.
Blinding	No group allocation was performed or was applicable. Blinding was therefore not applicable.

Reporting for specific materials, systems and methods

We require information from authors about some types of materials, experimental systems and methods used in many studies. Here, indicate whether each material, system or method listed is relevant to your study. If you are not sure if a list item applies to your research, read the appropriate section before selecting a response.

Materials & experimental systems

n/a	Involved in the study
☒	☐ Antibodies
☒	☐ Eukaryotic cell lines
☒	☐ Palaeontology and archaeology
☒	☐ Animals and other organisms
☒	☐ Clinical data
☒	☐ Dual use research of concern
☒	☐ Plants

Methods

n/a	Involved in the study
☒	☐ ChIP-seq
☒	☐ Flow cytometry
☐	☒ MRI-based neuroimaging

Magnetic resonance imaging

Experimental design

Design type

Resting state

Design specifications

Four resting-state scans, of length 14.4 minutes each, were acquired from each participant (2 RL and 2 LR).

Behavioral performance measures

No behavioural performance measures were used.

Acquisition

Imaging type(s)

Functional

Field strength

3

Sequence & imaging parameters

TR = 720 ms, TE = 33.1 ms, flip angle = 52 deg, FOV = 208x108 mm, matrix = 104x90, slice thickness = 2.0 mm, number of slices = 72 (2.0 mm isotropic), multi factor band = 8, and echo spacing = 0.58 ms.

Area of acquisition

Whole brain

Diffusion MRI ☐ Used ☒ Not used

Preprocessing

Preprocessing software

Freesurfer, FSL, Connectome Workbench

Normalization

Brains were normalized to fslr32k via the MSM-All registration

Normalization template

FSLR32K

Noise and artifact removal

CompCor, with five principal components from the ventricles and white matter masks, was used to regress out nuisance signals from the time series. In addition, the 12 detrended motion estimates provided by the Human Connectome Project were regressed out from the regional time series and the mean global signal was removed. No bandpass filtering was performed (see Supplementary Note 1). Also, participants listed in [53] under "3T Func- tional Preprocessing Error of all 3T RL fMRI runs in 25 Sub jects" or "Sub jects without Field Maps for Structural scans" were removed, leaving a total of 700 participants that were used for all the analyses. We parcellated the brain into 100 cortical [54] and 16 subcortical [55] regions.

Volume censoring

We removed participants from further analysis if any of their four resting scans had excessively large head motion, defined by having frames with greater than 0.2 mm frame-wise displacement or a derivative root mean square (DVARS) above 75.

Statistical modeling & inference

Model type and settings

All models used in this study were predictive dynamical system models. Different linear and nonlinear families of models were used. Details of each model family are provided in Methods.

Effect(s) tested

The nonlinearity of brain dynamics was tested using model comparisons between linear and nonlinear families of models. No ANOVA or factorial designs were used.

Specify type of analysis: ☒ Whole brain ☐ ROI-based ☐ Both

Statistic type for inference

(See Eklund et al. 2016)

fMRI data were primarily used at the parcel-level, using 100 cortical regions (Schaefer 100x7 atlas [61]) and 16 subcortical ones (Melbourne Scale I atlas [62]). Limited voxel-level analysis was also performed, as shown in Supplementary Figs. 12–14.

Correction	Multiple comparisons between pairs of models were corrected for using false discovery rate.

Models & analysis

n/a	Involved in the study
☐	☒ Functional and/or effective connectivity
☒	☐ Graph analysis
☐	☒ Multivariate modeling or predictive analysis

Functional and/or effective connectivity	Dynamical system modelling, including autoregressive models.
Multivariate modeling and predictive analysis	Dynamical system modelling using the prediction-error framework.
